# Isolation and Characterization of an Organobismuth Dihydride

**DOI:** 10.1021/jacs.5c09023

**Published:** 2025-08-05

**Authors:** Satoshi Kurumada, Nils Nöthling, Yue Pang, Nijito Mukai, Markus Leutzsch, Richard Goddard, Josep Cornella

**Affiliations:** † 28314 Max-Planck-Institut für Kohlenforschung, Kaiser-Wilhelm-Platz 1, 45470, Mülheim an der Ruhr, Germany

## Abstract

We report the synthesis, isolation, and structural characterization of an elusive organobismuth­(III) dihydride (Ar*–Bi­(III)–H_2_, **1**). The complex features a bulky and rigid ^*t*^Bu-M^s^Fluind ligand that permits complete spectroscopic characterization and SC-XRD. The compound is thermally unstable and decomposes to quantitatively form H_2_ and Ar*–Bi­(I) in a chemoselective intramolecular process. In addition to H_2_ formation, the presence of a Bi–H bond is supported by comparative spectroscopy (NMR and IR) with its deuterated analogue **1**-*d*_2_.

## Graphical abstract

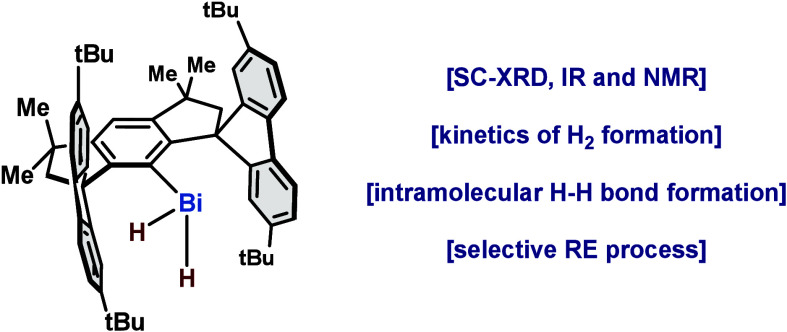

Recently, the field of bismuth redox catalysis has emerged as an orthogonal alternative to light main group and transition metal catalysis,^1–18^ thus offering a plethora of redox manifolds that can be harnessed in various contexts of synthesis. An in-depth analysis of the reported catalytic protocols to date reveals that a large portion invokes the presence of organobismuth hydrides as putative intermediates; for example, in transfer hydrogenations,^4,18^ reduction of nitrous oxide^7^ or azides,^16^ amide reductions,^10^ hydrodefluorinations,^8^ dehydrogenative O–Si coupling^19^ or HER (hydrogen evolution reactions),^20^ among others. Due to the high reactivity of the Bi–H bond, these intermediates are highly fleeting and have posed severe difficulties when attempting their isolation and characterization. Indeed, almost all evidence gathered so far has been largely restricted to their identification via HRMS or in solution, using NMR at low-temperature (Figure 1A).^4,8^ In addition to the relevance in catalytic strategies, bismuth hydrides have also spurred the minds of synthetic organometallic chemists for many years due to the intriguing Bi–H bond,^21–25^ as it represents a hydride species originating from the bond between two elements with dramatic differences in radius.^26–28^ Bi–H species are prone to rapid decomposition, generating H_2_ and generally unknown Bi byproducts. For example, BiH_3_ has been synthesized and its structure studied *in silico* and gas phase, before decomposing to Bi metal and H_2_.^21^ On the other hand, organobismuth monohydrides have been synthesized and studied in solution—both diaryl^22^ and dialkyl^23,24^ —and used in various organometallic endeavors.^25^ Power et al. reported the first and only thermally stable diorganylbismuth hydride, whose structure was confirmed by both SC-XRD (single crystal X-ray diffraction) and IR, and later NMR.^22^ The use of bulky Ar ligands provided the necessary steric congestion to stabilize the Bi–H bond. It was shown that upon heating, this mono hydride undergoes formation of dibismuthene (Ar–Bi=Bi–Ar)^22,29,30^ and H_2_. This and other bismuth hydrides have been shown to undergo hydrobismuthation to alkenes, alkynes and C=N bonds.^31^ Albeit the formation of H_2_ from Bi­(III) halides is a common strategy to access bismuthinidenes,^32^ not much is known about the putative organobismuth dihydrides.^33^ Since the main decomposition pathway is the formation of H_2_ and a low-valent “RBi”, it is not surprising that kinetic stabilization of two hydrides attached to Bi­(III), inasmuch as RBiH_2_, would pose severe difficulties. Whereas MeBiH_2_ has indeed been prepared and used in several inorganic recipes,^32^ the structure of organobismuth dihydrides still remains elusive. In this communication, we report the synthesis and characterization of the unique organobismuth­(III) dihydride (**1**), bearing a bulky and rigid ^*t*^Bu-M^s^Fluind backbone (Ar*, Figure 1B). The compound is stable below −20 °C, and was characterized by NMR, IR and SC-XRD. This molecule extrudes H_2_ above 0 °C and permits the study of the reductive elimination of dihydrogen from Bi­(III) to Bi­(I). Differently than in Power’s monohydride,^22^ kinetic isotope effect (KIE) and intermolecular experiments with the corresponding deuterated analogue suggest this process to be intramolecular and H_2_-selective, thus avoiding reductive demetalation of the C–Bi bond.

**Figure 1:**
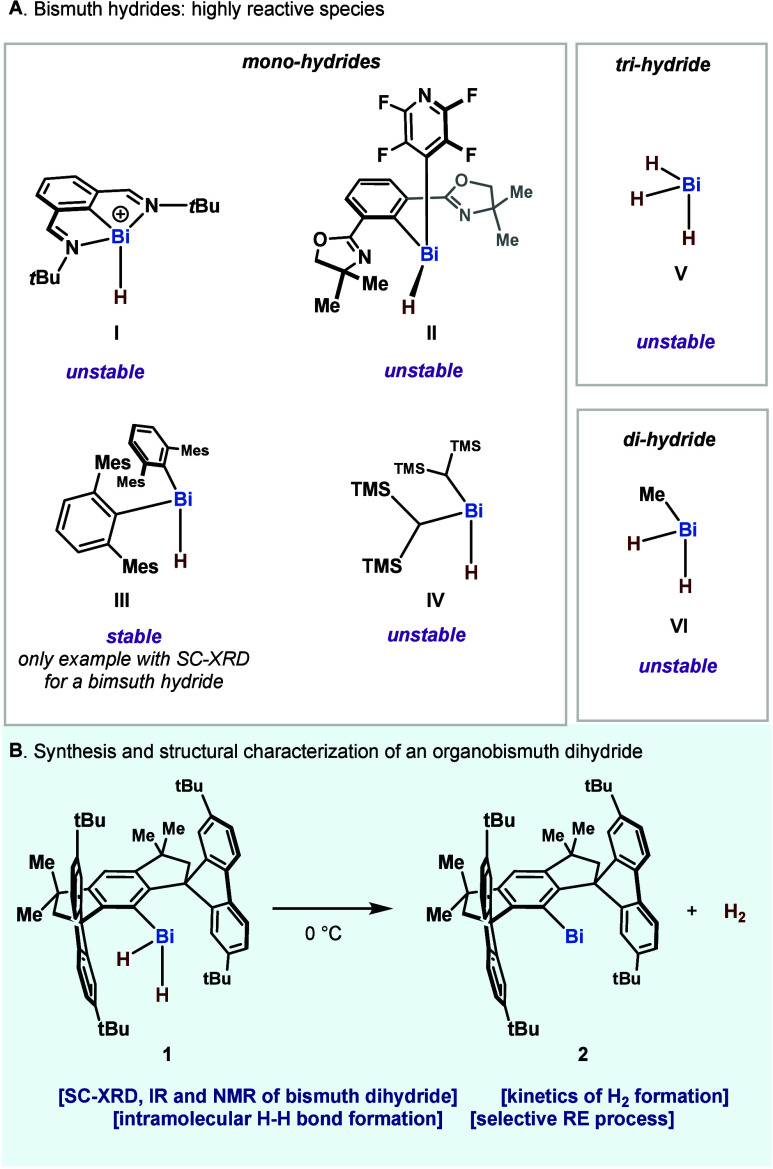
Bismuth hydrides: state-of-the-art.

For some years, our group has been interested in the chemistry and reactivity of low-valent Bi­(I) compounds, as a means to unlock catalytic redox processes spanning from small molecule activation to organic synthesis.^4,7–9,11–16,18,30,34–37^ Within this context, we reported the synthesis of the first stable monocoordinated organobismuthinidene featuring a bulky and rigid ^*t*^Bu-M^s^Fluind backbone (**2**).^30^ During the investigation of its chemical reactivity, we realized that **2** remained reluctant to H_2_ cleavage under 1 atm at 25–60 °C, as judged by the absence of bismuth dihydride formation. This reluctance contrasts with the rapid H_2_ cleavage of the analogous Ar*–Sb­(I)^38^ or a similarly bulky Ar–N­(I),^39,40^ which rapidly react with H_2_ to form the stable Ar*–SbH_2_ and Ar–NH_2_ respectively.

The targeted organobismuth dihydride was synthesized as illustrated in Figure 2A. The arylbismuth dibromide **3** reacted with an excess LiAlH_4_ at −40 °C generating ^*t*^Bu-M^s^Fluind-BiH_2_ **1** as an off-white powder. Maintaining the temperature <−40 °C was crucial to prevent decomposition of **1** (*vide infra*). Compound **1** could also be obtained using a neutral aluminum hydride, DIBAL-H in toluene or THF. From this latter reaction, colorless crystals suitable for SC-XRD analysis were obtained by layering pentane over the toluene solution of **1**. The solid state structure of **1** is shown in Figure 2B and 2C. In late refinement cycles, traces of positive residual electron density were found at a distance of about 1.8 Å from the central Bi atom. Based on their relative position to the heavy atom, these were assumed to be the positions of the two H atoms. Their coordinates had to be fixed during refinement in order to achieve a convergence. Due to the significant difference in scattering power between Bi and H, the reliability of such assignments is limited. The obtained structure exhibited a monomeric form, similar to our previously reported monosubstituted Bi­(I) **2**,^24^ but with some distinct structural features. The Bi–C bond (2.265(1) Å) was slightly shorter than that in **1** (2.2783(9) Å), indicating differences in the electronic and steric environments. The presence of Bi–H bonds is evident however in various geometrical observations. For example, the increased distance between the fluorene moiety of the ligand—measured between the centroids of the flanking six-membered rings—is 6.960 Å and 7.096 Å in **1**, while it shortens to 6.777 Å and 6.762 in **2**. In addition, structure **1** is isomorphous to the Ar*Sb–H_2_, recently reported by our group, which shows a similar distortion of the ligand.^38^ The presence of hydrogen atoms was also confirmed by NMR spectroscopy. The ^1^H NMR spectrum of **1** showed a broad signal at 8.05 ppm, attributed to the Bi–H_2_. The pronounced broadening at 20 °C (w_1/2,293 K_ = 22.2 Hz) sharpens upon cooling (w_1/2,223 K_ = 5.5 Hz). This behavior contrasts with the Ar*Sb–H_2_ complex, which displays a sharp doublet (^5^ *J*_HH_ = 0.4 Hz; w_1/2,298 K_ = 0.4 Hz) at 1.24 ppm. We therefore attribute the broadening to the large quadrupole moment of the ^209^Bi nucleus (100%, I = 9/2) and the low local symmetry at the bismuth center. The Bi–H ^1^H NMR chemical shift falls between the two reported values for a bismuth hydride: the highly deshielded (2,6-diMesPh)_2_Bi–H (19.6 ppm) reported by Power^22^ and the highly shielded (TMS_2_CH)_2_Bi–H from Breunig (3.24 ppm).^23^ To further confirm the Bi–H signal, the deuterated analogue **1**-*d*__2__ was synthesized from **3** and LiAlD_4_, and the disappearance of the 8.04 ppm signal was observed by ^1^H NMR (Figure 2D, middle). Also, the ^2^H NMR spectrum of **1**-*d*__2__ confirmed the deuterated nature of the signal (Figure 2D, bottom). The ^1^H NMR signals of the ligand scaffold remained sharp from 298 to 193 K. This is in contrast to **3**, which showed a broadening of these signals at low temperatures. This indicates a faster rotation of the hydrides around the Bi–C bond compared to the bulkier halides. All of the ^1^H and ^13^C chemical shifts are observed in the diamagnetic region. Compared to **2**, the ^13^C_ipso_–Bi chemical shift in **1** (125.7 ppm) lies in the normal diamagnetic range, whereas the corresponding carbon in the triplet bismuthinidene **2** resonates at a very uncommon frequency (−203.3 ppm). A similarly pronounced difference is observed for the proton at C4 (7.43 ppm in **1** vs −1.04 ppm in **2**). To assess whether the broad Bi–*H*_*2*_ signals originate from a Bi­(III)-dihydride rather than a putative singlet Bi­(I)–dihydrogen complex, we determined the *T*_1_ relaxation time.^41^ In the case of dihydrogen complexes, the protons normally display *T*_1_ values below 100 ms,^42^ whereas dihydride complexes have similar relaxation times to other protons in the complex. The *T*_1_ relaxation time of Bi–*H*_2_ was 1.32 s at 223 K, thus strongly suggesting that **1** is a dihydride. Further confirmation of the Bi–H bonding was provided by IR spectroscopy. A Bi–H stretching absorption was observed at 1717 cm^–1^, which was in good agreement with the reported bismuth monohydride^22^ and calculated bismuth trihydride.^43^ In contrast, the IR spectrum of **1**-*d*__2__ showed no absorption at this frequency. Instead, an increasing intensity of the absorption at around 1250 cm^–1^ was observed. We think that Bi–D stretching absorption would be observed in this region due to the overlap with other signals derived from the ^*t*^Bu-M^s^Fluind ligand. The calculated Bi–D stretching absorption of **1**-*d*_2_ was 1224.56 and 1225.53 cm^–1^, in the range of previously reported values.

**Figure 2:**
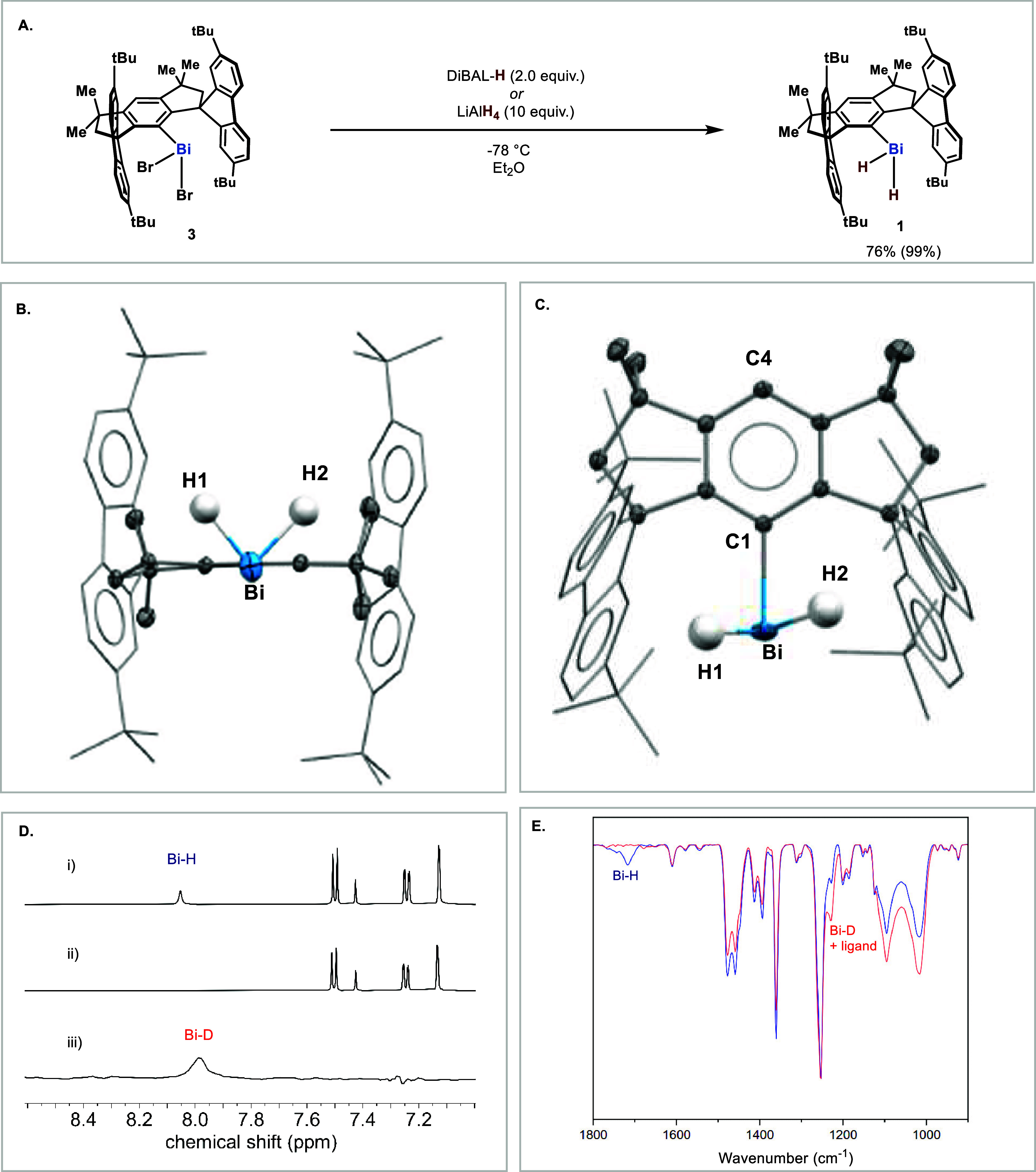
(A) Synthesis of organobismuth dihydride **1**. Isolated yield; in brackets, ^1^H NMR yield using 1,2,4,5-tetramethylbenzene as internal standard. (B) Molecular structure of **1** (front view) with thermal ellipsoids at 50% probability and all hydrogen atoms except Bi bounded hydrogen are omitted for clarity. (C) Molecular structure of **1** (top view) with thermal ellipsoids at 50% probability, and all hydrogen atoms except Bi bounded hydrogen are omitted for clarity. (D) Snippet of the NMR data: (i) ^1^H NMR spectrum of **1**, (ii) ^1^H NMR spectrum of **1**-*d*_2_, (iii) ^2^H NMR spectrum of **1**-*d*_2_. (E) IR spectra of **1** (blue line) and **1**-*d*_2_ (red line).

Heating a THF solution of **1** led to the formation of the corresponding Bi­(I) **2** through reductive H–H coupling (Figure 3A). H_2_ evolution is clearly observed by ^1^H NMR. VT-NMR analysis of **1** revealed that the elimination process begins at 0 °C. At least two plausible mechanisms—intermolecular or intramolecular coupling—are considered for the H–H bond formation. Reported dialkyl bismuth monohydrides decompose through H_2_ evolution,^23^ and the resulting dialkyl bismuth moieties combine to form dibismuthane. Such homocoupling of a potential dialkyl bismuth radical suggests that an intermolecular mechanism is being operative for dialkyl bismuth hydrides. For the highly sterically encumbered diaryl bismuth hydrides, however, such an intermolecular pathway is not feasible. Instead, an intramolecular C–H coupling occurs, producing Ar–H and dibismuthene—the dimerization product of the potential bismuthinidene intermediate.^22^ To determine the mechanism operating in the reductive elimination from **1**, H–D scrambling tests were performed. When **1** and **1**-*d*__2__ were mixed in a 1:1 ratio at −50 °C (223 K), no evidence for scrambling of the Bi–H and Bi–D was observed. This mixture was then allowed to convert to Bi­(I) over 1 h at 25 °C in THF. The ^1^H NMR spectrum of the mixture confirmed the presence of H_2_ and HD albeit in >50:1 ratio (Figure 3B). This suggests that intermolecular H–D exchange did not occur and points to an intramolecular process. To further investigate the reaction mechanism, the decomposition of **1** and **1**-*d*_2_ was independently monitored by ^1^H NMR spectroscopy, and the KIE was measured. The decay of **1** and **1**-*d*__2__ followed pseudo-first-order kinetics, with rate constants *k*_H_ = (2.98 ± 0.01) × 10^–1^ h^–1^ and *k*_D_ = (3.79 ± 0.01) × 10^–2^ h^–1^. A large normal KIE of *k*_H_/*k*_D_ = 7.9 ± 0.1 was measured from two independent reactions with **1** and **1**-*d*_2_ (Figure 3C). Although interpretations of large KIEs have been previously discussed for organometallic complexes,^44^ the origin of such a large value is still unclear at present. Quantitative data on the activation parameters could be obtained from an Eyring plot analysis, from which a negative entropy of activation (Δ*S* ^‡^ = −12.4 ± 1.1 cal K^–1^ mol^–1^) is apparent. An enthalpy barrier of Δ*H* ^‡^ = 18.9 ± 0.3 kcal mol^–1^ and an activation energy of Δ*G* ^‡^ = 22.6 ± 0.6 kcal mol^–1^ at 25 °C were also obtained. It should be noted that an activation energy of +23.7 kcal mol^–1^ was obtained when computing the intramolecular H–H bond formation leading to **1** (Figure S41).

**Figure 3:**
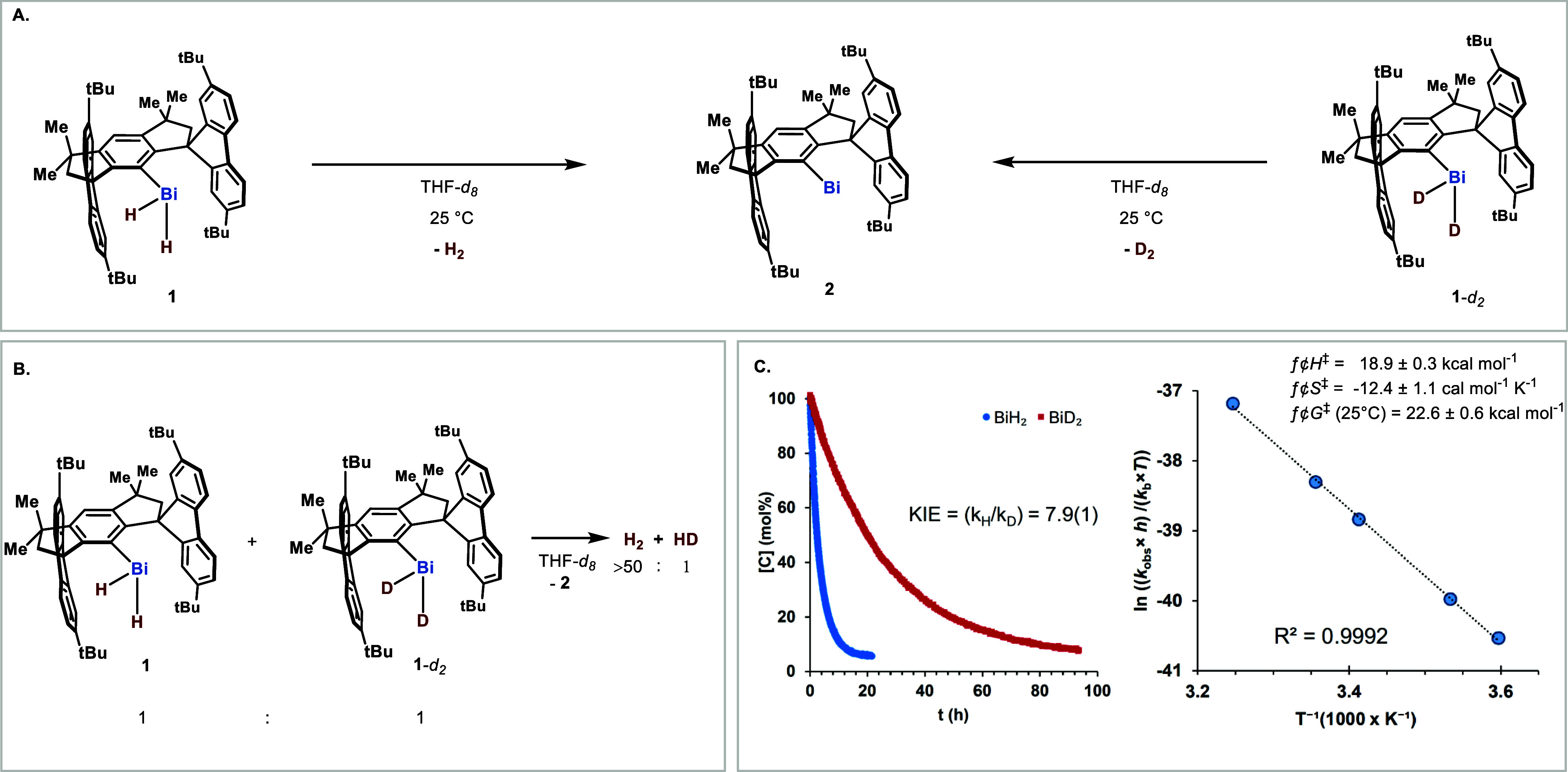
(A) Reductive H–H or D–D coupling was performed from **1** or **1**-*d*_2_. (B) H–D scrambling experiment. (C) KIE profiles (left) and an Eyring plot for the thermal decomposition of **1** (right).

In summary, we successfully synthesized, isolated, and characterized the first well-defined organobismuth­(III) dihydride complex (**1**). The use of a bulky and rigid ^*t*^Bu-M^s^Fluind ligand enabled comprehensive spectroscopic analysis and X-ray diffraction studies. The complex exhibits thermal instability, undergoing regioselective decomposition to generate H–H and **2**. The presence of a Bi–H bond is supported by a side-by-side NMR and IR study of its deuterated analogue **1**-*d*_2_. The results provided herein represent a significant step forward in the understanding of organobismuth chemistry, and provides key insights for the design of future bismuth-catalyzed redox transformations.

## Supplementary Material



## Notes

Experimental procedures and analytical data (^1^H and ^13^C NMR, HRMS, IR, and theoretical calculations) for new compounds. (PDF)
