# Supplementary material for: Isolation and Characterization of an Organobismuth Dihydride
Source: J Am Chem Soc. 2025 Aug 5;147(33):29636–41. doi: 10.1021/jacs.5c09023 (PMC12371872; doi:10.1021/jacs.5c09023)
Supplement: Supplementary file 1 [file ja5c09023_si_001.pdf]

Supporting Information

Isolation and Characterization of an Organobismuth Dihydride

Satoshi Kurumada, Nils Nöthling, Yue Pang, Nijito Mukai, Markus Leutzsch, Richard Goddard, Josep Cornella[a,] *

[a] Max-Planck-Institut für Kohlenforschung, Kaiser-Wilhelm-Platz 1, Mülheim an der Ruhr, 45470, Germany.

cornella@kofo.mpg.de

Table of Contents

1. General Information S3
2. Synthesis and Characterization Data of Organobismuth Dihydride S4
2.1. Isolation of Organobismuth Dihydride S4
2.2. Crystallization of Organobismuth Dihydride S8
2.3. NMR yield calculation of Organobismuth Dihydride with Internal Standard S8
2.4. T_1 relaxation measurement S10
3. Synthesis and Characterization Data of Organobismuth Dideuteride S11
3.1. Isolation of Organobismuth Dideuteride S11
3.2. NMR yield of Organobismuth Dideuteride S14
4. Investigation of the H–H Coupling S15
4.1. H-D Scrambling test S15
4.2. NMR Reaction Monitoring S16
4.2.1. General Procedure for Data Acquisition and Processing S16
4.2.2 Kinetic Isotope Effect S17
4.2.3. Eyring plot S18
5. Reactivity Investigation of Organobismuth Dihydride S23
5.1. Reaction with N-Bromosuccinimide S23
5.2. Unsuccessful Substrates S24
5.2.1. Attempt to Hydrobismuthation S24
5.2.2. Attempt to Trapping of Hydrogen Radical S24
5.2.3. Attempt to the Reaction with Proton S25
5.2.4. Attempt to the Reaction with Base S25
6. X-ray Crystal Structure Analysis of Organobismuth Dihydride S26
6.1 Single crystal structure analysis of Organobismuth Dihydride S26
6.2. Refinement details S33
6.3. Database search S36
6.4. Geometrical aspects S37
7. Theoretical Study S41
7.1. General information. S41
7.2. Theoretical study for Bi–H stretching S41
7.3. Theoretical study for Bi–D stretching S43
7.4. Theoretical study of dynamics S44
7.5. Coordinates S45

1. General Information

Unless otherwise stated, all manipulations were performed under argon using standard Schlenk line techniques or in an MBraun argon-filled glove box.

Instruments: NMR data were recorded on a Bruker AVIII HD 300 MHz, Bruker AVIII HD 400 MHz, Bruker AVIII 500 MHz or Bruker AVNeo 600 MHz NMR spectrometers. ^{1}H and ^{13}C chemical shifts are reported relative to the solvent residual peaks as an internal reference. For ^{1}H NMR the following residual proton peaks of the deuterated solvents were used: THF-d_8, δ 1.72. For ^{13}C NMR: THF-d_8, δ 67.21. ^{13}C spectra were acquired with broadband ^{1}H decoupling unless mentioned otherwise. IR spectra was acquired at the Technical University of Dortmund in a Cary 630 FTIR spectrometer with Dial Path Technology with solid samples and analyzed by FTIR MicroLab software.

Chemicals: DCM, THF, THF-d_8, toluene, *n*-pentane, diethyl ether were distilled from the proper drying agents and stored over 3 Å or 4 Å molecular sieves under argon prior to use. 3 Å or 4 Å molecular sieves were activated at 200 °C under high vacuum (1×10^{-4} bar) for 3 d. DCM, THF, THF-d_8, toluene, *n*-pentane, diethyl ether were degassed through freeze-pump-thaw cycle. Complex **3** was prepared according to the reported method.[1] Unless otherwise noted, all reagents were obtained from commercial suppliers and used without further purification.

2. Synthesis and Characterization Data of Organobismuth Dihydride

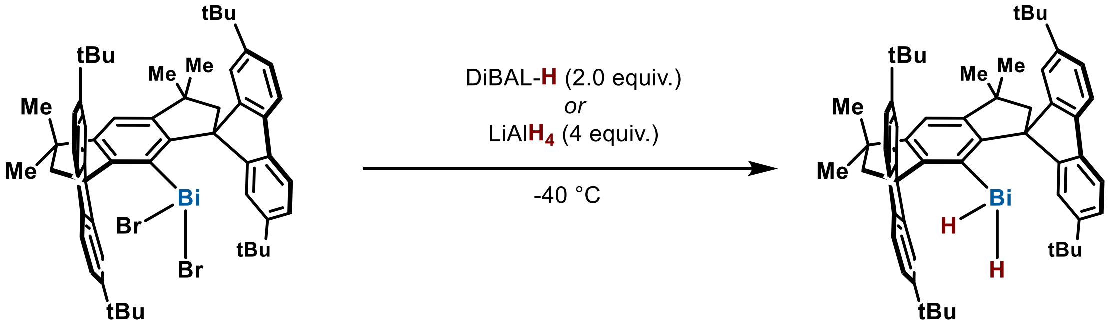

3 1

2.1. Isolation of Organobismuth Dihydride

To a mixture of **3** (55.3 mg, 50 μmol) and $LiAlH_4$ (7.6 mg, 200 μmol), precooled Et_2O (–78 °C, 4 mL) was added. After stirring for 5 min, the yellow color of **3** disappeared. The resulting mixture was then allowed to warm to –40 °C. At this point, the solvent was evaporated in the Schlenk line while keeping the temperature at –40 °C. The resulting mixture was extracted with precooled DCM (–78 °C, 5 mL) and evaporated to dryness, yielding the product as a white powder (36.2 mg, 76%).

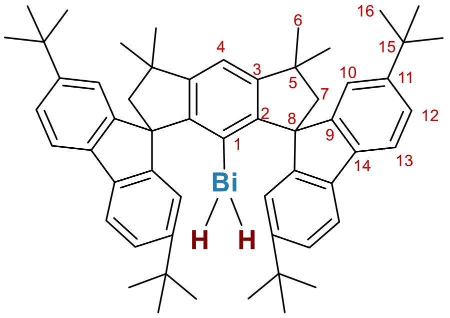

^{1}H NMR (500 MHz, THF-*d*$_8$, 223 K): δ 8.08 (s, 2H, Bi-H), 7.51 (d, *J* = 10 Hz, H-13), 7.43 (s, 1H, H-4), 7.26 (broad, d, *J* = 7.5 Hz, 5.0 Hz, 4H, H-12), 7.14 (broad, s, 2H, H-10), 2.31 (s, 4H, H-8), 1.58 (s, 12H, H-6), 1.22 (s, 36H, H-16).

^{13}C NMR (125 MHz, THF-*d*$_8$, 223 K): δ 155.2 (C-3), 154.7 (C-9), 151.2 (C-2), 150.6 (C-11), 140.0 (C-14), 125.7 (C-1), 124.8 (C-12), 122.1 (C-10), 119.9 (C-13), 117.8 (C-4), 67.9 (C-8), 59.3 (C-7), 43.0 (C-5), 35.5 (C-15), 32.7 (C-6), 32.1 (C-16).

IR: $\tilde{\nu}$ = 1717 cm^{-1} (br, Bi–H). Decay of the absorption peak over time was observed.

M.p.: M.p. is unclear since **1** is unstable even at room temperature.

HRMS (APPI): Calc'd for $C_{56}H_{66}Bi$ [***Bi***-H]$^{+}$ 947.49630; found 947.4953.

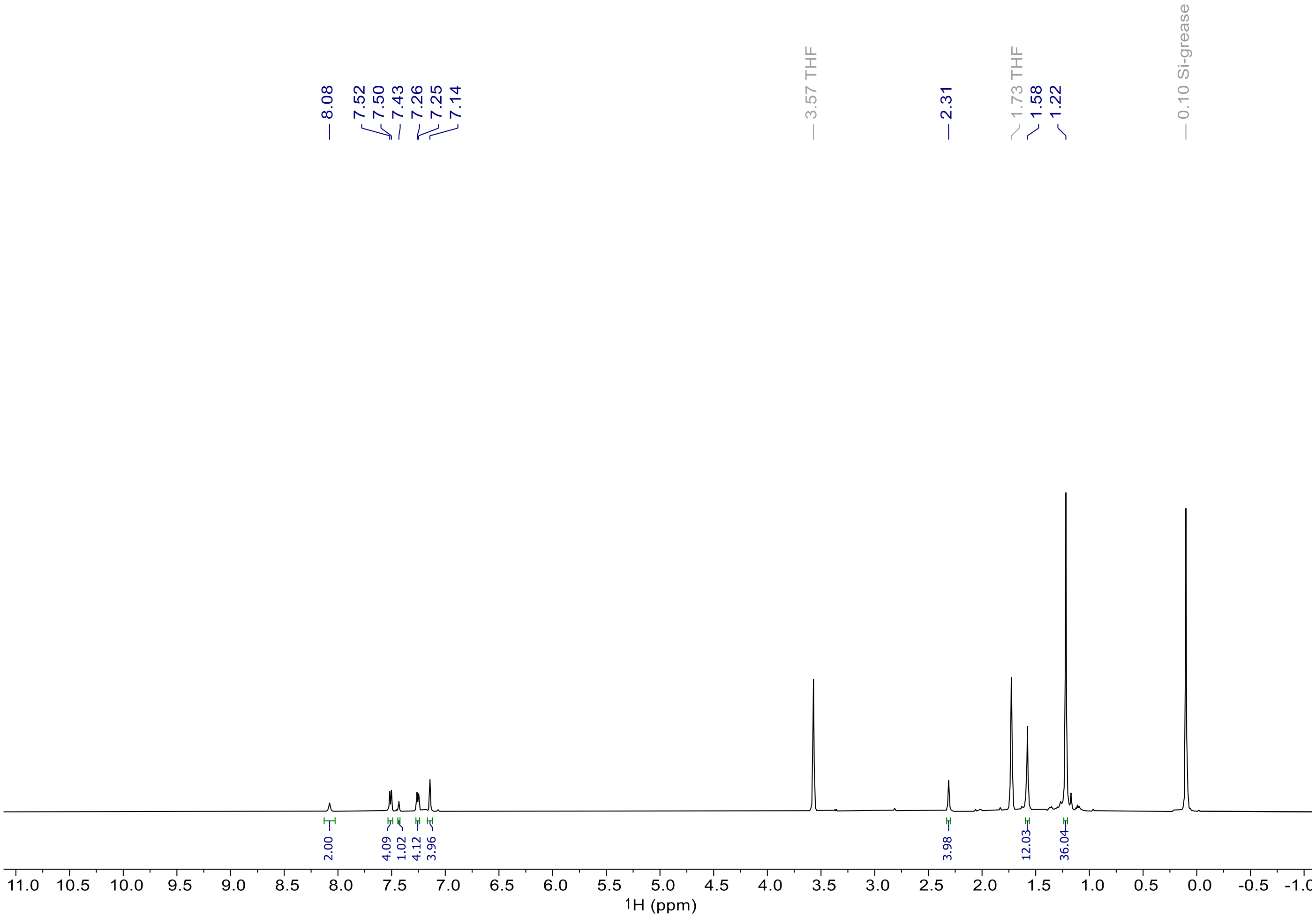

Figure S1. ^{1}H NMR spectrum of **1**, 500 MHz, THF-d_8, 223 K.

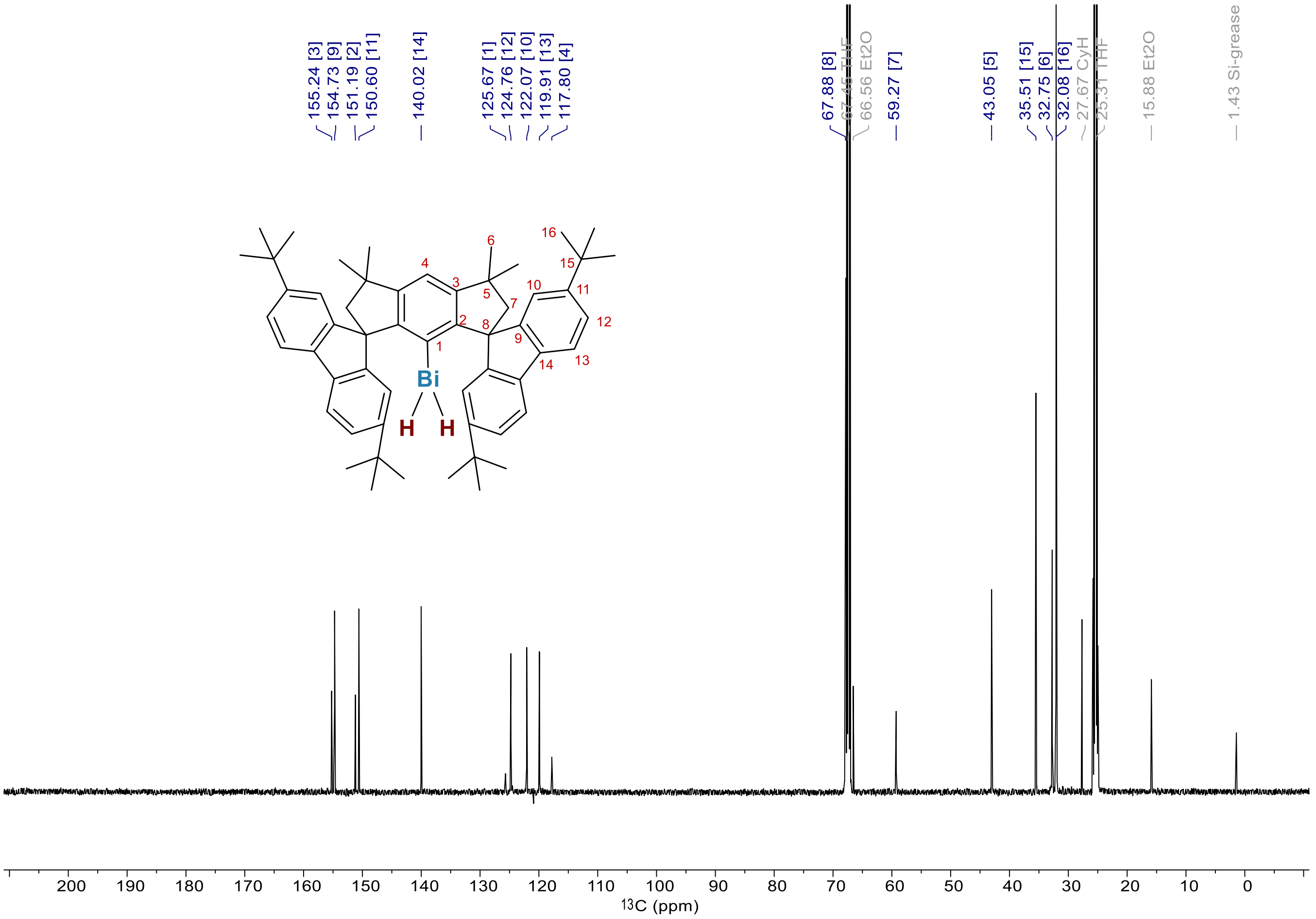

Figure S2. ^{13}C NMR spectrum of **1**, 125 MHZ, THF-d_8, 223 K.

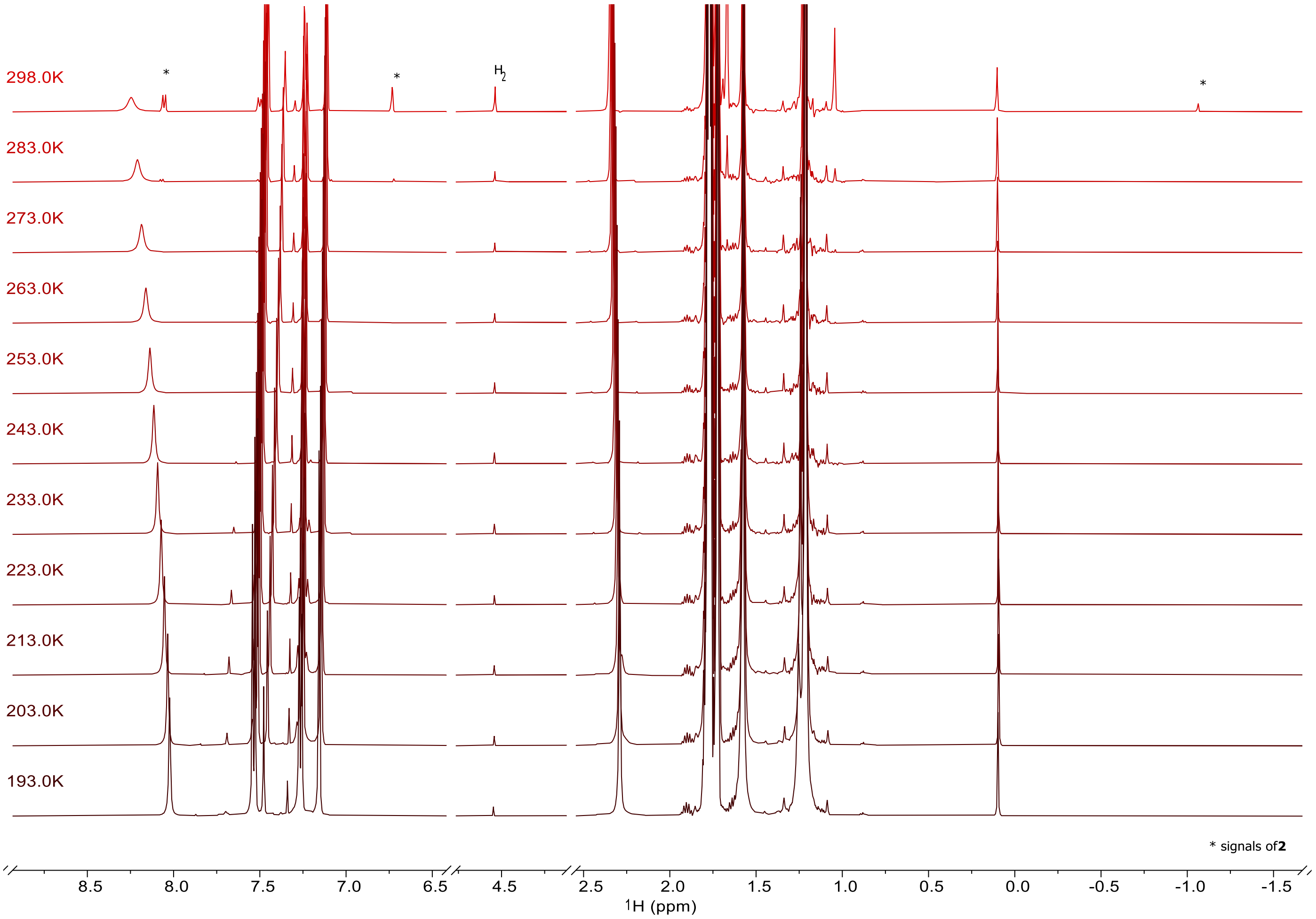

Figure S3. ^1H NMR spectra of **1** in THF-d_8 acquired at variable temperatures ranging from 193 K to 298 K. Above 273 K the formation of **2** and the increase of the H_2 concentration in solution is observable.

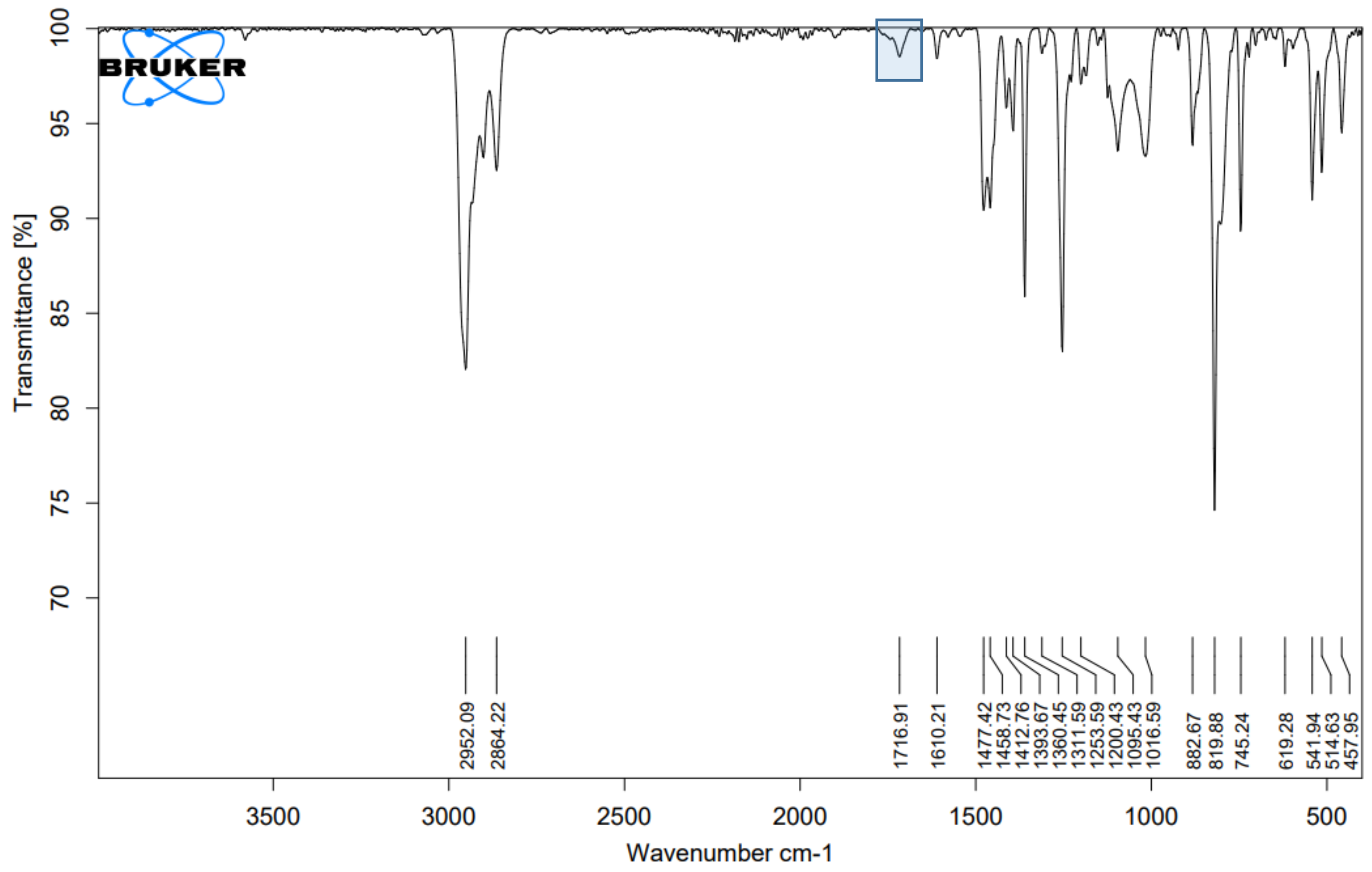

Figure S4. IR spectrum of **1**.

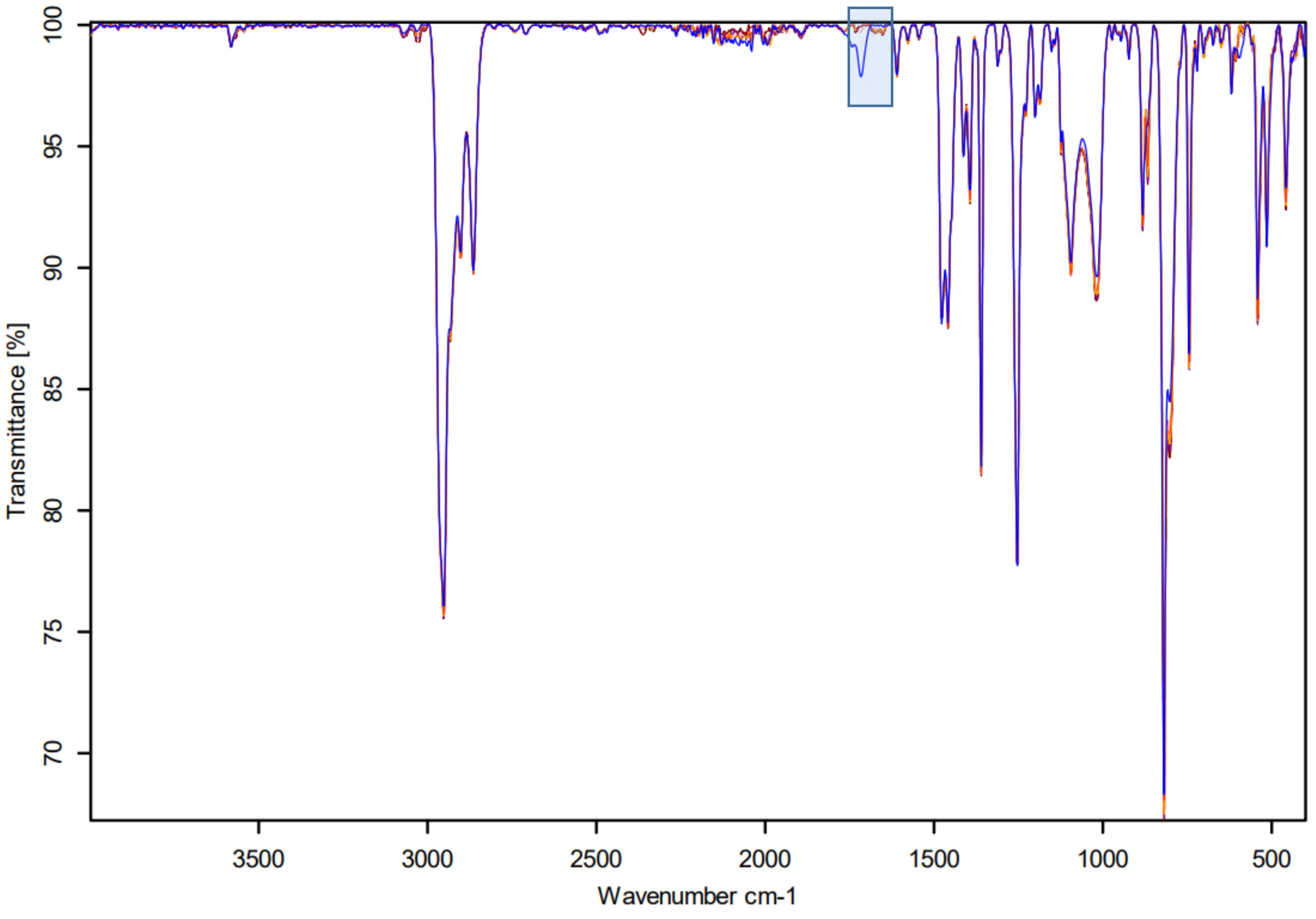

Figure S5. Overlay of IR spectra of **1** at t = 0 h (blue), t = 1 h (orange), t = 2 h (pink), and t = 6 h (red).

2.2. Crystallization of Organobismuth Dihydride

To a cooled toluene (–78 °C, 10 mL) solution of **3** (55.3 mg, 50 μmol), DIBAL-H (1M in hexane, 100 μL, 100 μmol) was slowly added. Then, the mixture was warmed to –40 °C and stirred for 1 h. After this time, 1 mL of pentane was carefully layered on the solution at the same temperature, and let the solution stand until crystals appeared (crystals suitable for a single-crystal X-ray diffraction analysis appeared after approx. 15 h).

2.3. NMR yield calculation of Organobismuth Dihydride with Internal Standard

To a mixture of **3** (11.1 mg, 10 μmol), $LiAlH_4$ (1.6 mg, 40 μmol), and 1,2,4,5-tetramethylbenzene (1.3 mg, 10 μmol, internal standard), precooled THF-*d*8 (–78 °C, 600 μL) was added. After stirring 5 min, the yellow color of **3** disappeared, and the mixture was allowed to warm to –40 °C. The insoluble inorganic salts were removed by filtration under Ar at –40 °C. ^{1}H NMR spectrum of the resulted colorless solution was recorded to calculate NMR yield.

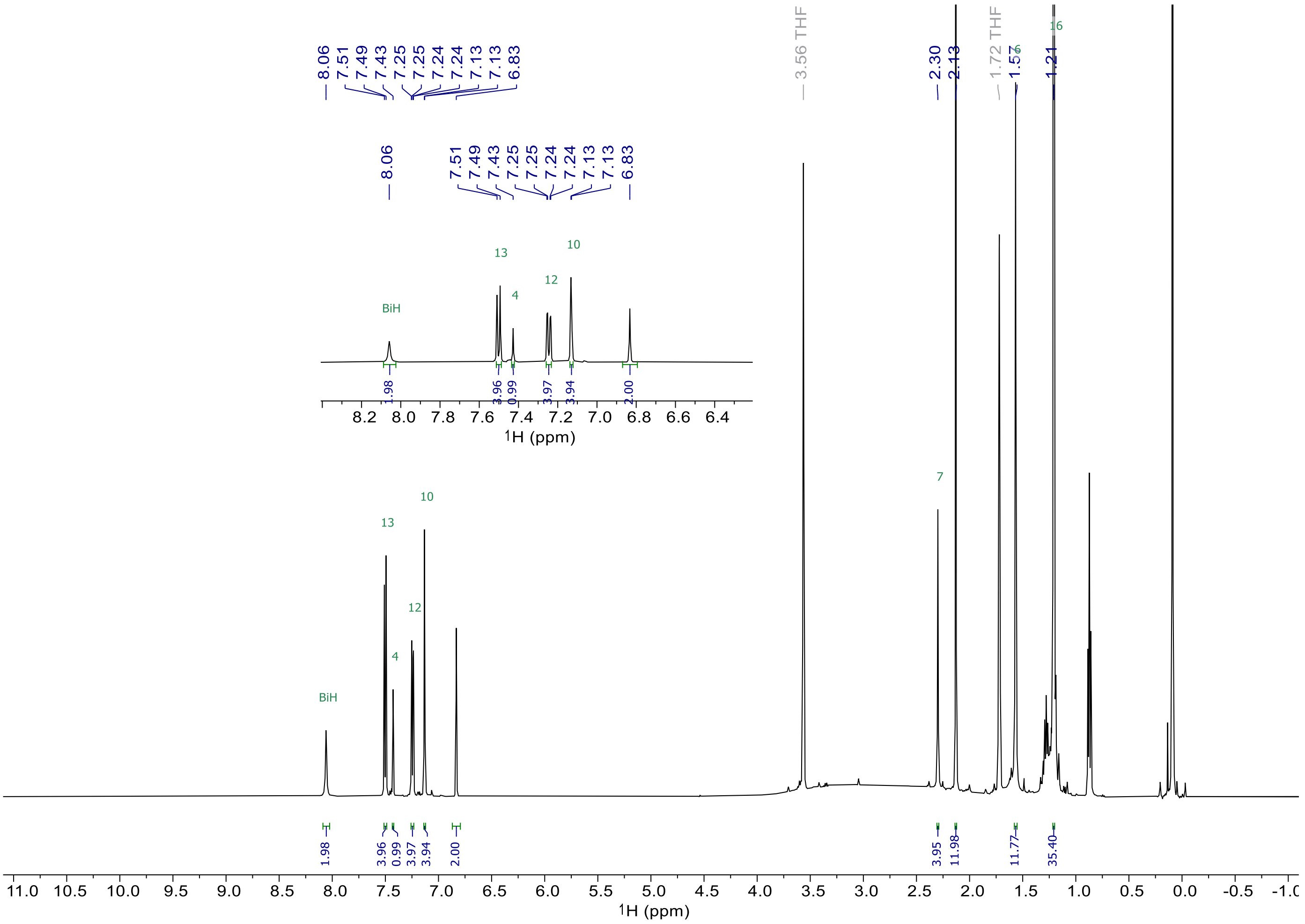

Figure S6. ^{1}H NMR spectrum (500 MHz, THF-*d*8, 223 K) of **1** with 1,2,4,5-tetramethylbenzene as internal standard used for NMR yield determination ($LiAlH_4$)

Note: The NMR yield can also be calculated in the same manner from the reaction of **3** and DIBAL-H.
Example: to a cooled THF-*d*8 (–78 °C, 600 μL) solution of **3** (11.1 mg, 10 μmol), DIBAL-H (1M in hexane, 20 μL, 20 μmol) was slowly added. Then, a ^{1}H NMR spectrum of the resulting colorless solution was recorded to calculate NMR yield.

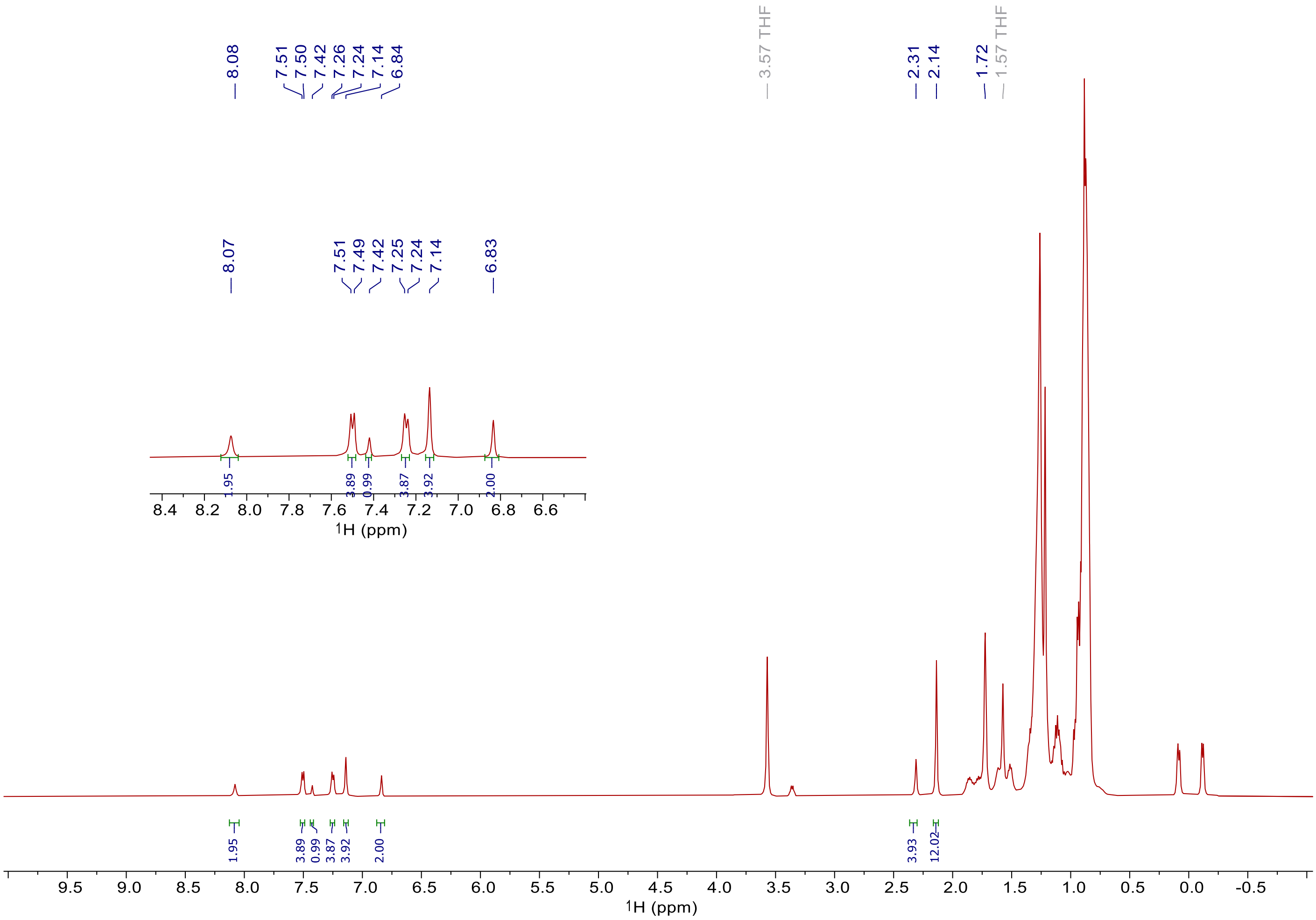

Figure S7. ^{1}H NMR spectrum (500 MHz, THF-d_8, 223K) of **1** with 1,2,4,5-tetramethylbenzene as internal standard used for NMR yield determination (DIBAL-H).

2.4. T_1 relaxation measurement

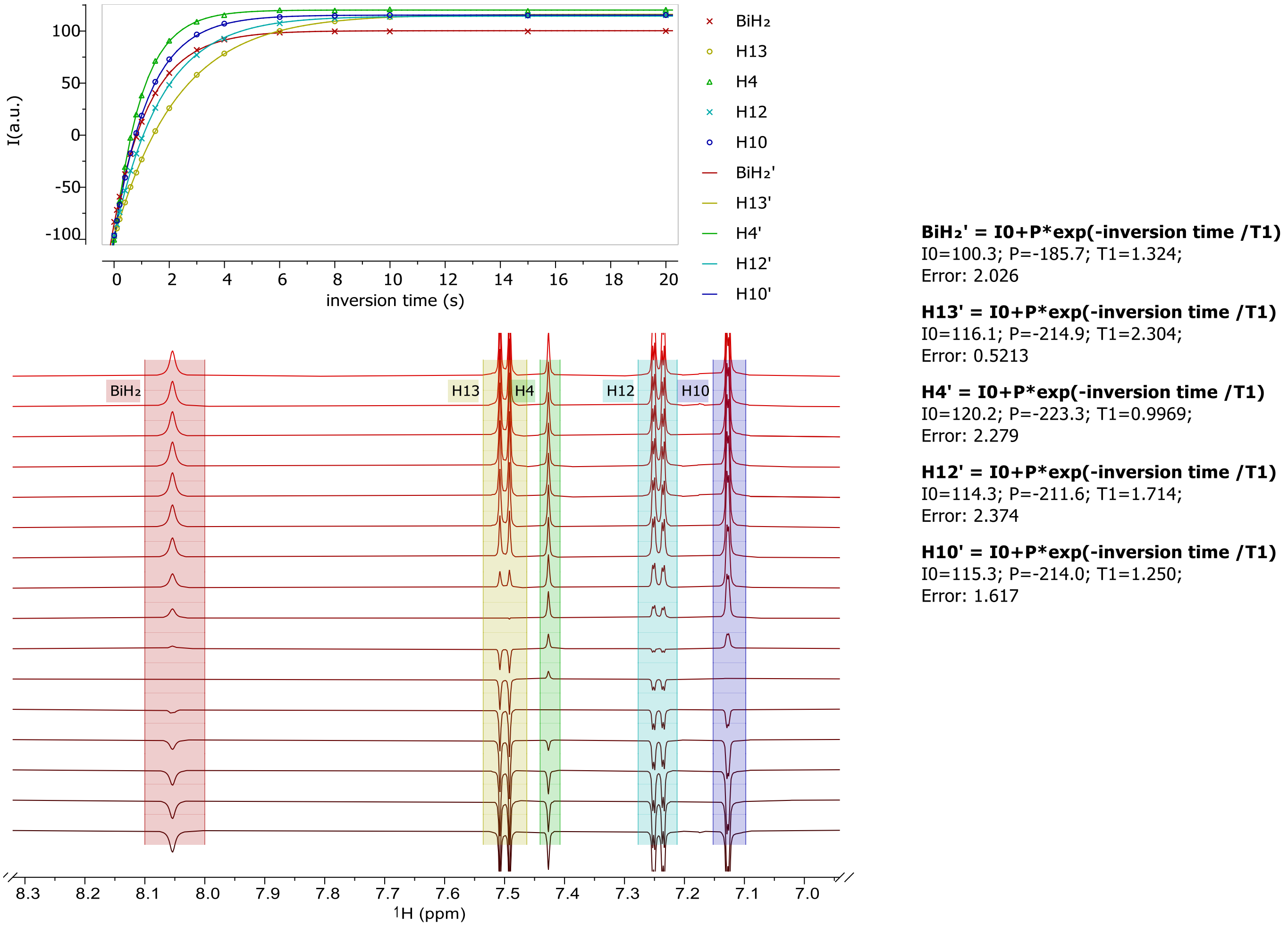

Figure S8. Spin–lattice relaxation time (T_1) analysis of the aromatic signals and the BiH_2 signal of **1** using inversion recovery pulse sequence (Bruker sequence: t1ir; 500 MHz, THF-d_8, 223 K).

3. Synthesis and Characterization Data of Organobismuth Dideuteride

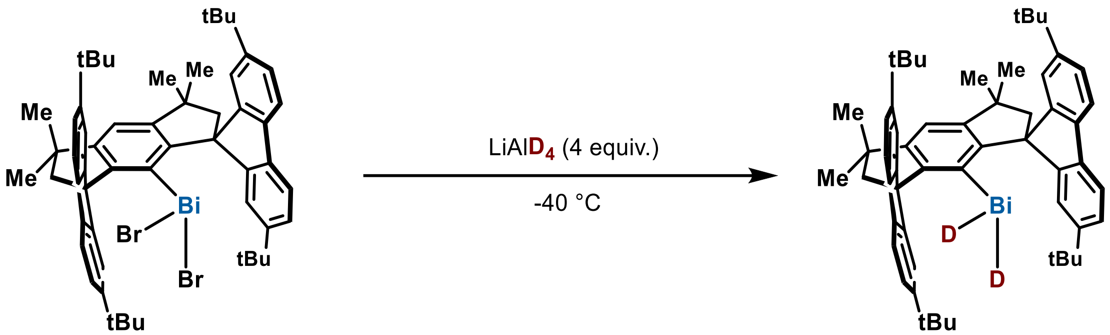

3 **1**-d_2

3.1. Isolation of Organobismuth Dideuteride

To a mixture of **3** (55.3 mg, 50 µmol) and $LiAlD_4$ (8.4 mg, 200 µmol, 98 %D), precooled Et_2O (–78 °C, 5 mL) was added. After stirring the mixture for 5 min, the yellow color of **3** disappeared. The resulting mixture was then allowed to warm to –40 °C. At this point, the solvent was evaporated in the Schlenk line while keeping the temperature at –40 °C. The resulting mixture was extracted with precooled DCM (–78 °C, 5 mL) and evaporated to dryness, yielding **1-*d*₂** as a white powder (38.1 mg, 80%).

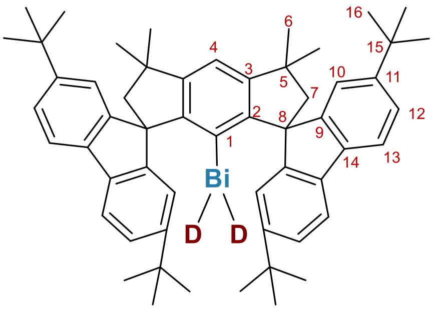

^{1}H NMR (500 MHz, THF-*d*$_8$, 223 K): δ 7.51 (d, *J* = 10 Hz, 4H, H-13), 7.43 (s, 1H, H-4), 7.25 (dd, *J* = 7.5 Hz, 5.0 Hz, 4H, H-12), 7.14 (d, *J* = 2.0 Hz, 4H, H-10), 2.31 (s, 4H, H-8), 1.57 (s, 12H, H-6), 1.21 (s, 36H, H-16).
^{2}H NMR (76.7 MHz, THF-*d*$_8$, 223 K): δ 7.99 (br, Bi–D).
^{13}C NMR (125 MHz, THF-*d*$_8$, 223 K): δ 155.2 (C-3), 154.7 (C-9), 151.2 (C-2), 150.6 (C-11), 140.0 (C-14), 125.7 (C-1), 124.8 (C-12), 122.1 (C-10), 119.9 (C-13), 117.8 (C-4), 67.9 (C-8), 59.3 (C-7), 43.0 (C-5), 35.5 (C-15), 32.7 (C-6), 32.1 (C-16).
IR: $\tilde{\nu}$ = 1254 cm^{-1} (br, Bi–D: signal overlaps with additional ligand bands).
M.p.: M.p. is unclear since **1**-d_2 is unstable even at room temperature.
HRMS (ESI): Calc'd for $C_{56}H_{65}DBi$ [***Bi***-D]$^+$ 948.50258;found 948.50194.

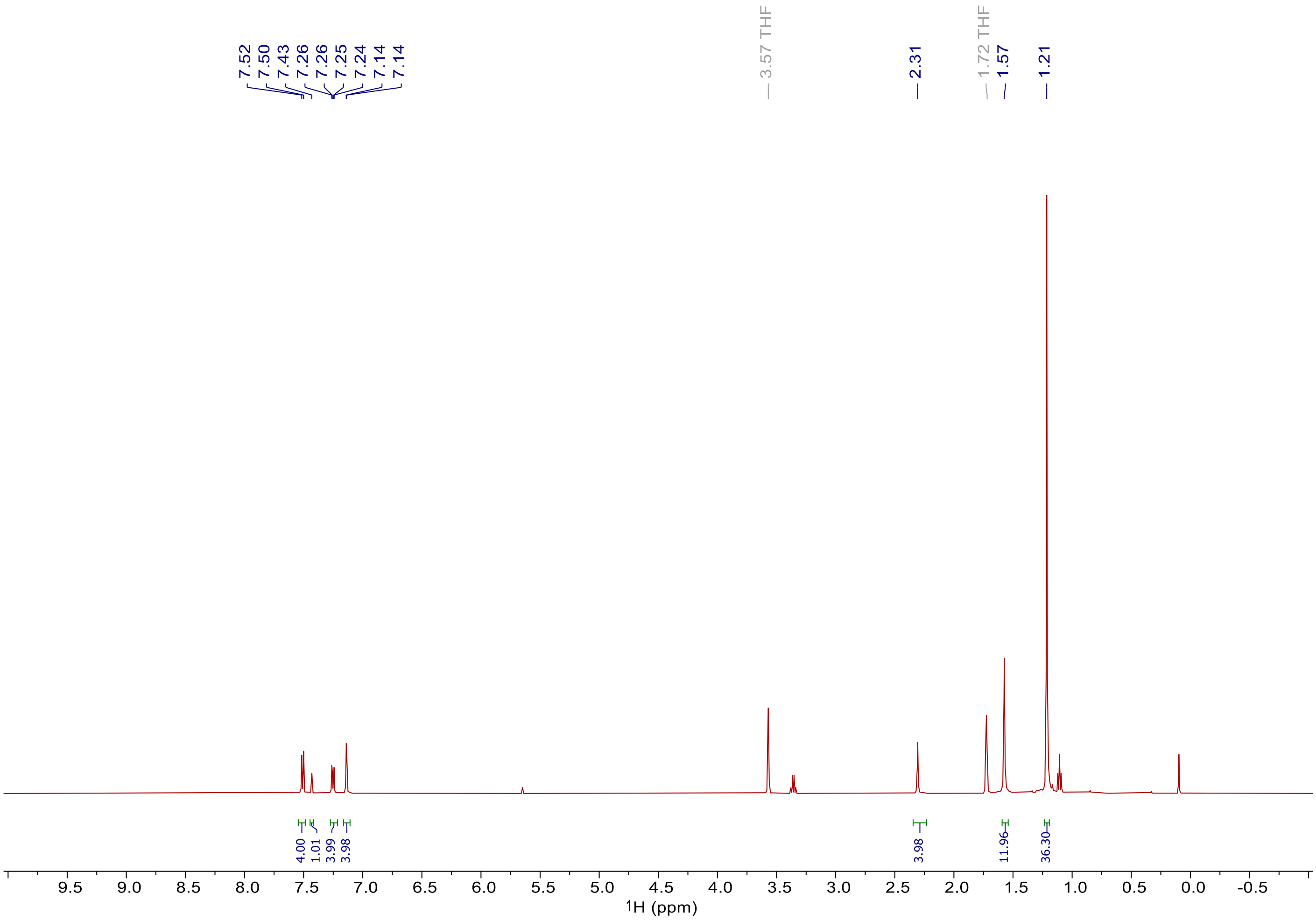

Figure S9. ^{1}H NMR spectrum of **1-*d*$_2$**, 500 MHz, THF-d_8, 223 K.

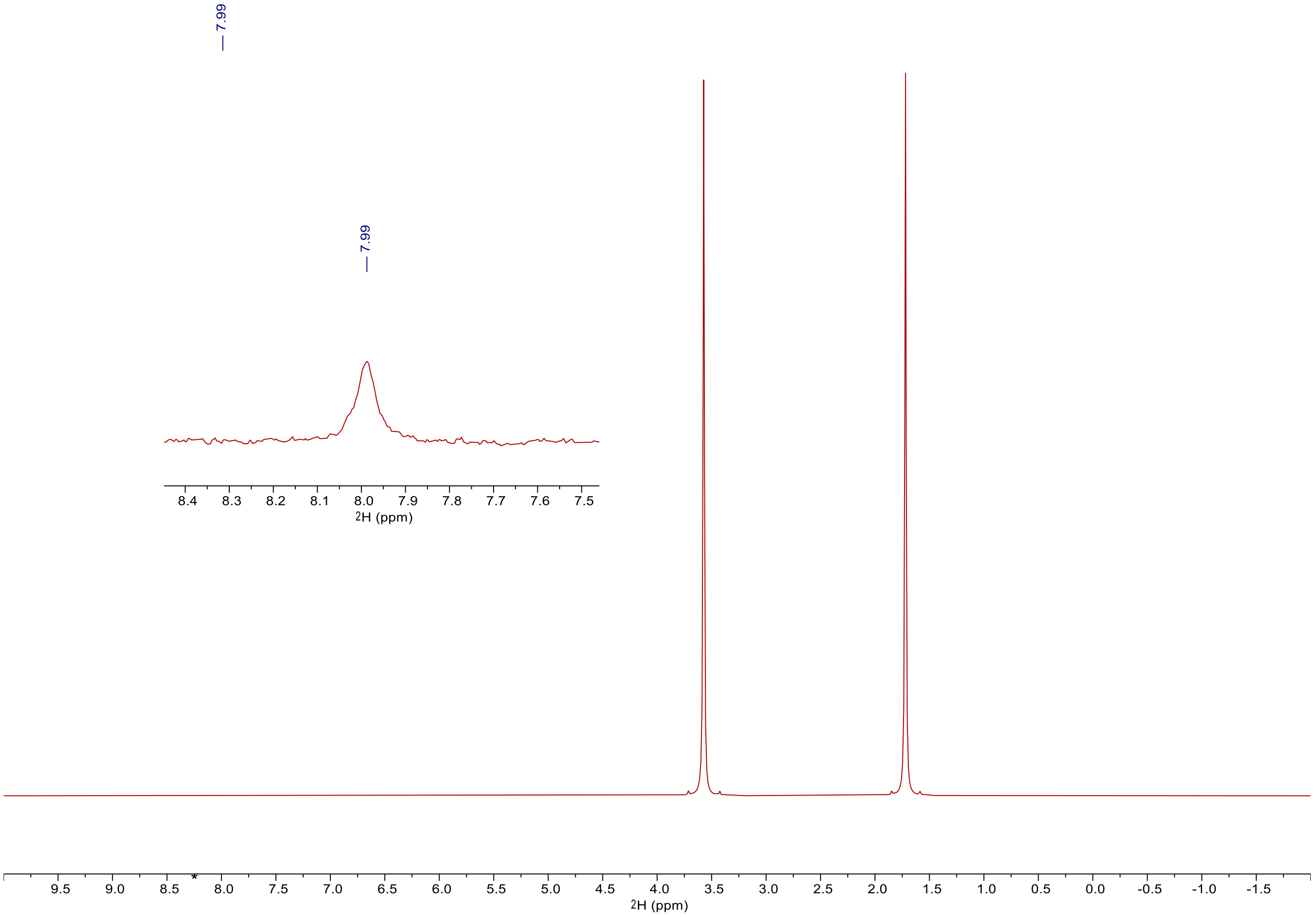

Figure S10. ^{2}H NMR spectrum of **1**-d_2, 76.7 MHz, THF-d_8, 223 K.

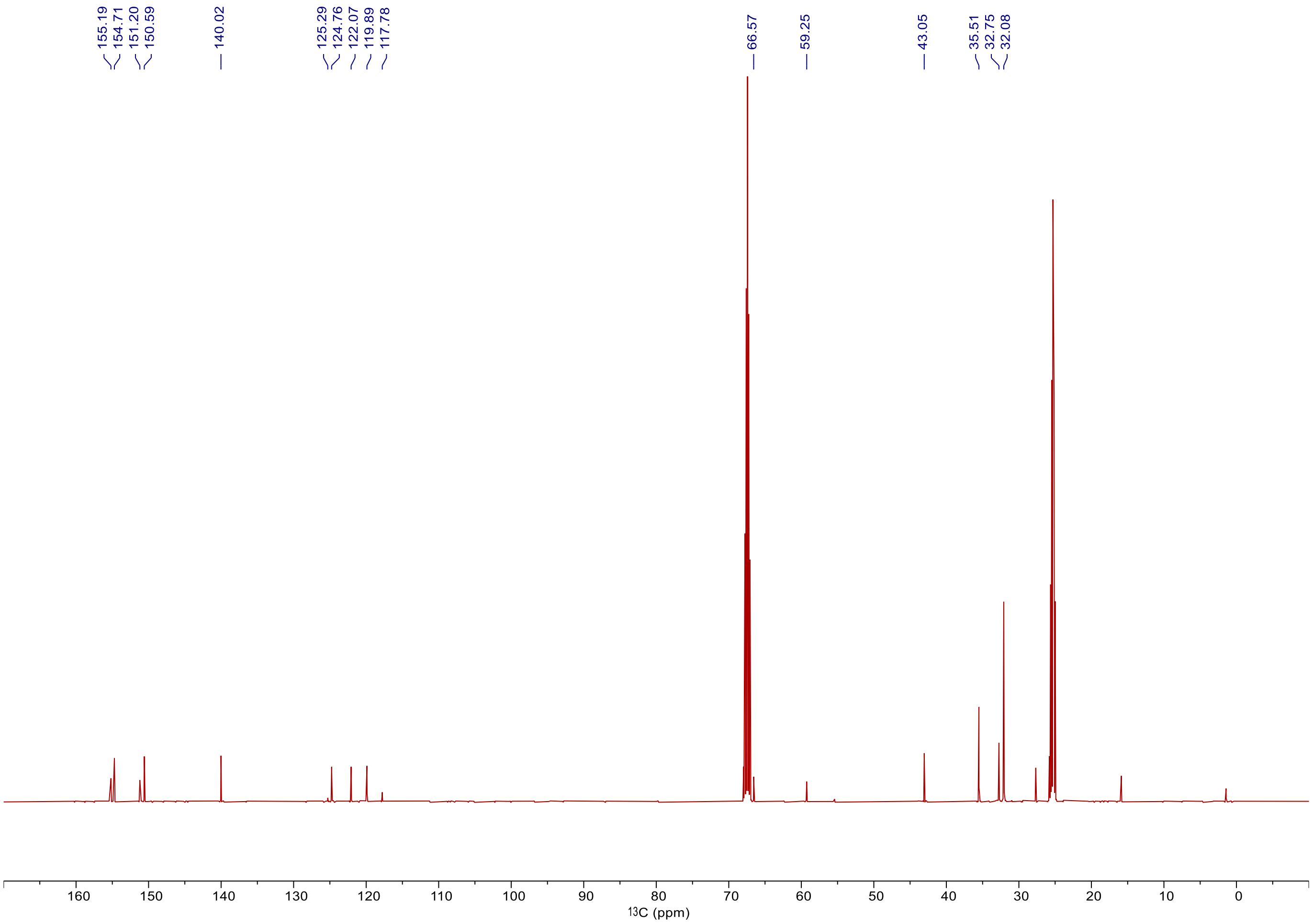

Figure S11. ^{13}C NMR spectrum of **1**-*d*$_2$, 125 MHz, THF-*d*$_8$, 223 K.

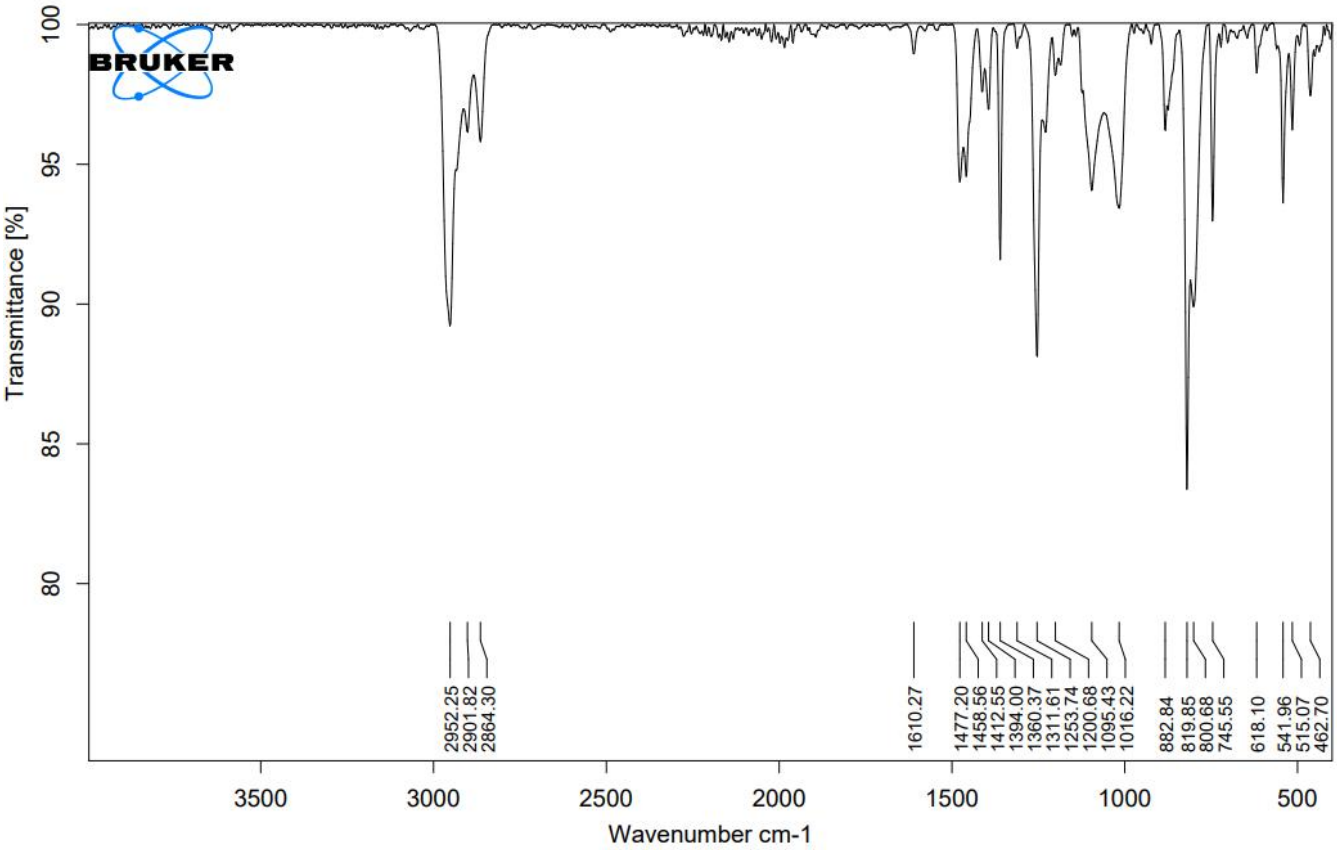

Figure S12. IR spectrum of **1**-*d*$_2$.

3.2. NMR yield of Organobismuth Dideuteride

To a mixture of **3** (11.1 mg, 10 μmol), $LiAlD_4$ (1.7 mg, 40 μmol) and 1,2,4,5-tetramethylbenzene (1.3 mg, 10 μmol, internal standard), precooled THF-d_8 (–78 °C, 600 μL) was added. After stirring the mixture for 5 min, the yellow color of **3** disappeared, and the mixture was allowed to warm to –40 °C. Then, the insoluble inorganic salts were removed by filtration and ^{1}H NMR recorded to calculate the NMR yield.

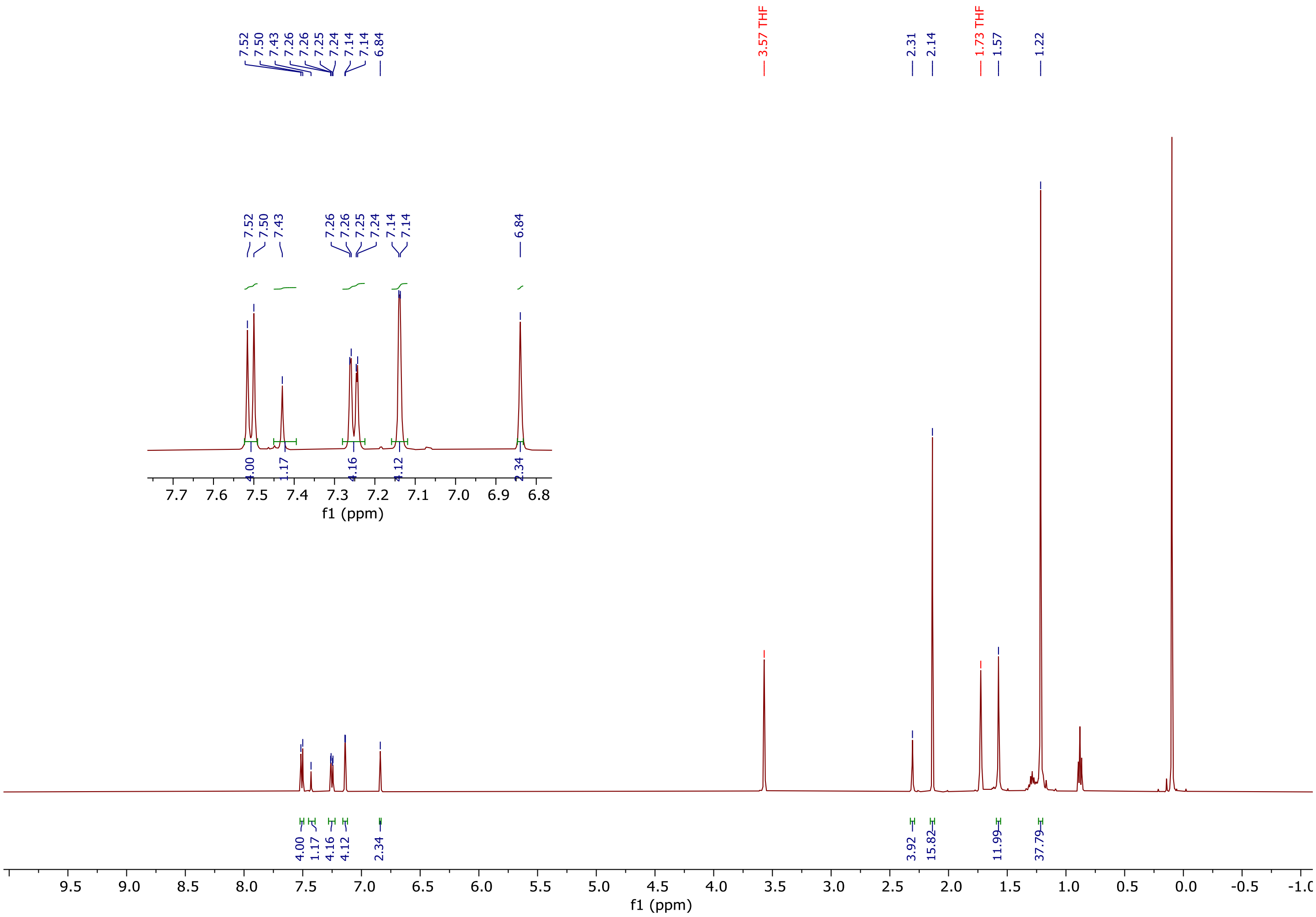

Figure S13. ^{1}H NMR spectrum (500 MHz, THF-d_8, 223 K) of **1**-d_2 with 1,2,4,5-tetramethylbenzene as internal standard used for NMR yield determination.

4. Investigation of the H–H Coupling

4.1. H-D Scrambling test

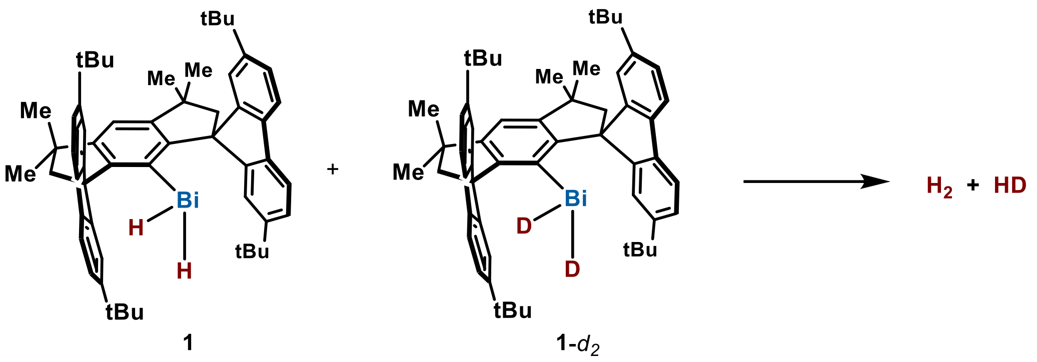

To a mixture of **3** (11.1 mg, 10 μmol) and $LiAlH_4$ (3.8 mg, 100 μmol), precooled Et_2O (–78 °C, 5 mL) was added. After stirring the mixture for 5 min, the yellow color of **3** disappeared, and the mixture was warmed to –40 °C. Then, the solvent was evaporated while keeping the temperature at –40 °C. The resulting mixture was extracted with precooled DCM (–78 °C, 5 mL) to obtain **1** in solution. In the same way, a solution of **1**-d_2 was prepared with $LiAlD_4$ (8.4 mg, 200 μmol) in a separate Schlenk flask. The two solutions of **1** and **1**-d_2 were combined and evaporated to dryness, yielding a white powder. The powder was dissolved in 1 mL of THF-d_8 and ^{13}C NMR spectrum was acquired at 223 K.

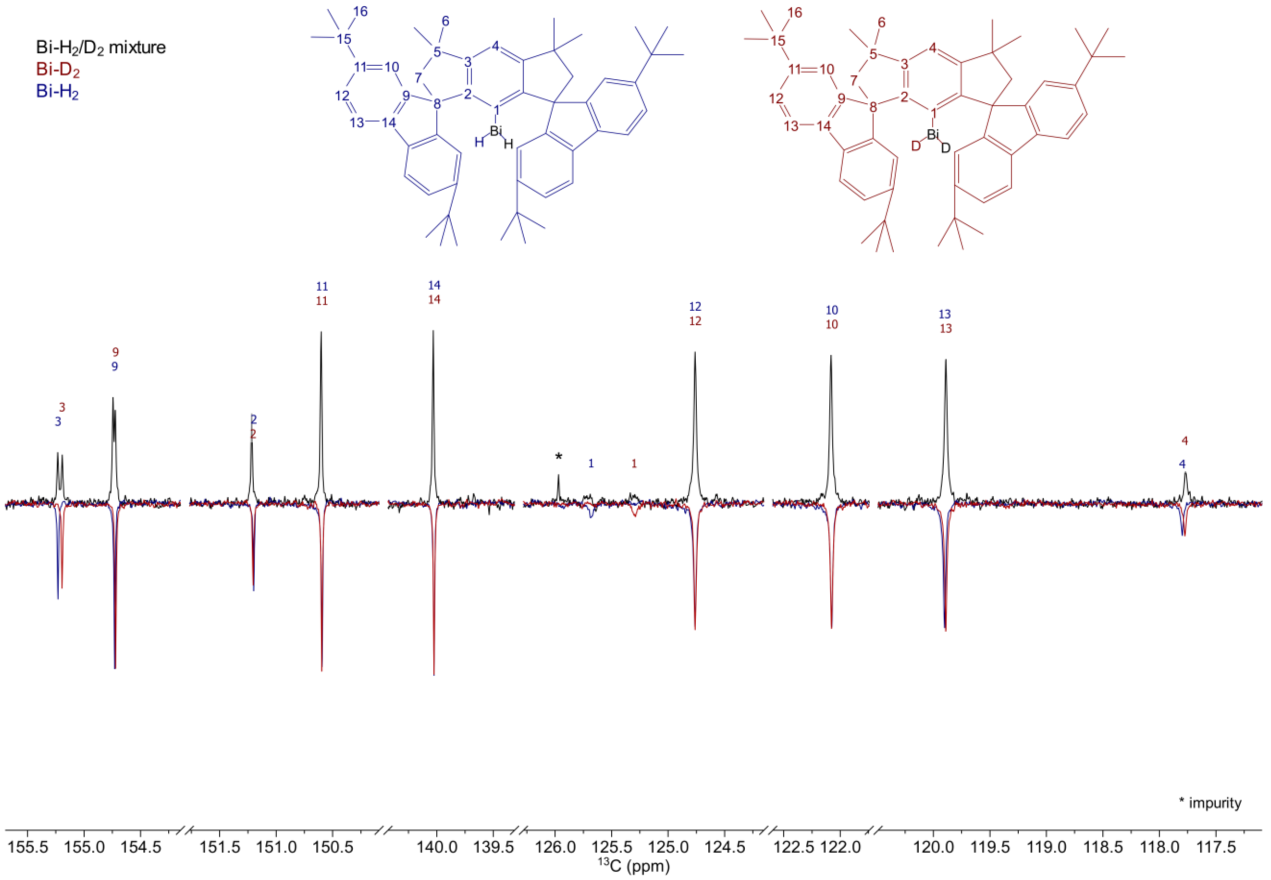

Figure S14. Superimposed ^{13}C NMR spectra of a 1:1 mixture of **1** and **1**-d_2 in comparison to the respective spectra of the pure samples. (125 MHz, 223 K, THF-d_8).

The data showed no indication of rapid formation of a new species. The ^{13}C signals at positions C1 and C3 exhibit characteristic isotope shifts, and no additional signals were observed that would suggest a rapid hydrogen/deuterium exchange.

After the measurement, the sample was kept for approximately 1 h at 298 K (25 °C) and then the ^{1}H NMR was re-acquired at 243 K. After this time a total conversion of 22% was obtained. When considering the additional parahydrogen-content, the overall H_2:HD ratio was ~50:1.

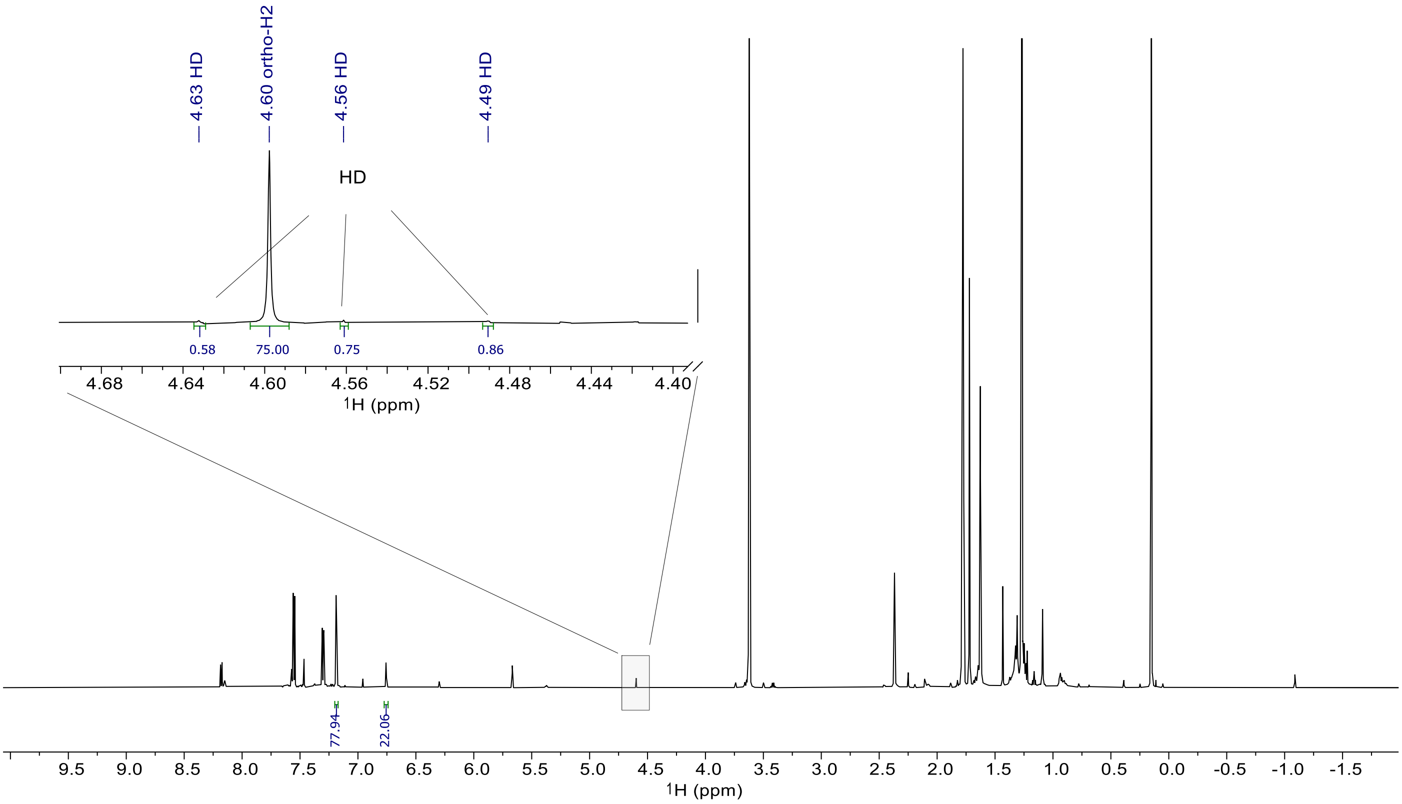

Figure S15. ^{1}H NMR spectrum of the reaction mixture of **1** and **1**-d_2 after 1 h reaction time at 298 K; 600 MHz, 243 K, THF-d_8.

4.2. NMR Reaction Monitoring

4.2.1. General Procedure for Data Acquisition and Processing

The freshly prepared sample was transported in a dry ice bath to the magnet, where it was quickly transferred to the NMR probe preheated or precooled to the specified temperature. Subsequently, single-scan ^{1}H NMR spectra were acquired every 1–5 min until the desired conversion was achieved. The data were then imported into MNOVA 15.0.0 using the Reaction Monitoring Plugin and processed (baseline correction and phasing). Reaction profiles were generated by integrating the ^{1}H NMR signal of H-10 at 7.1 ppm.

An exemplary reaction profile is shown in the following figure:

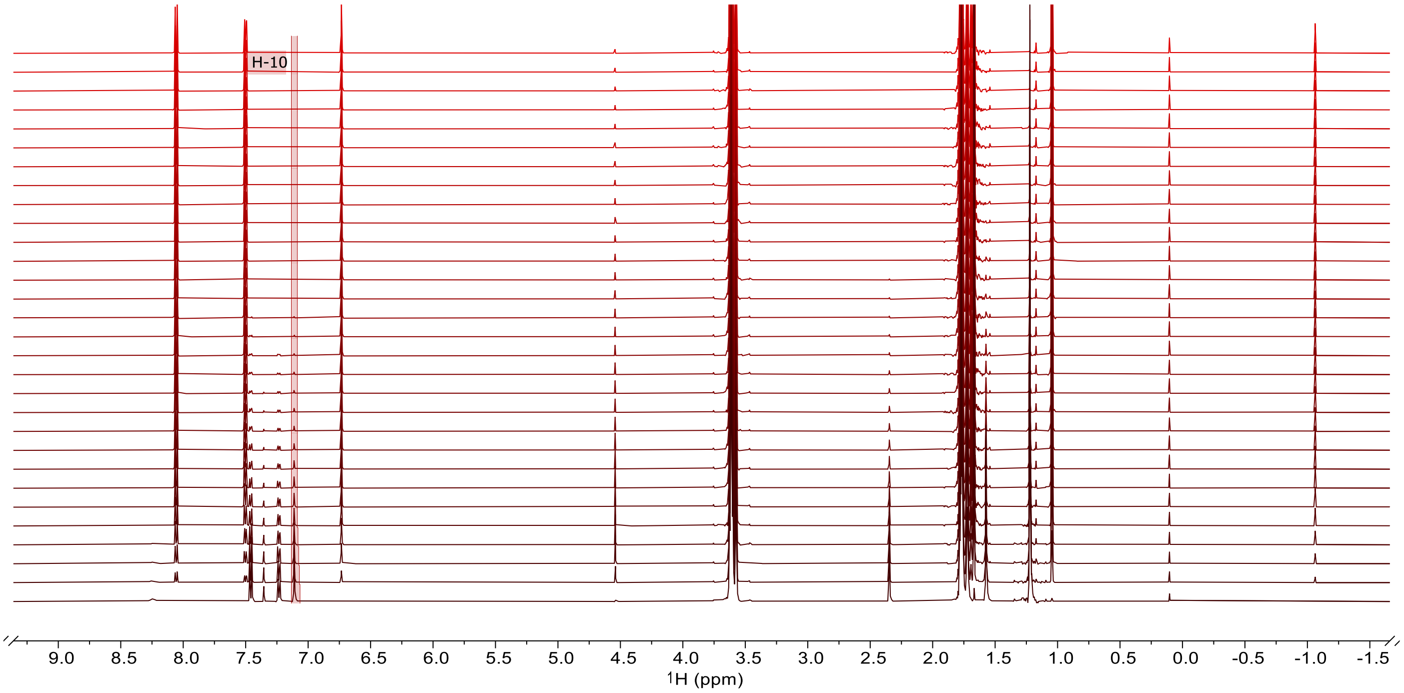

Figure S16. Stacked ^{1}H NMR spectra showing the conversion of **1** to **3** at 298 K (25 °C) taken at different time points, 500 MHz, THF-d_8.

The obtained reaction profiles were then exported to OriginLab Origin 2019b, where they were fitted non-linearly to a first-order exponential decay to determine $k_{obs.}$

4.2.2 Kinetic Isotope Effect

To a mixture of **3** (5.5 mg, 10 µmol) and $LiAlH_4$ (7.6 mg, 200 µmol), precooled Et_2O (–78 °C, 1 mL) was added. After stirring the mixture 5 min, the yellow color of **3** disappeared, and the mixture was allowed to warm to –40 °C. Then, the solvent was evaporated while keeping the temperature at –40 °C. The resulting mixture was extracted with precooled DCM (–78 °C, 2 mL) and evaporated to dryness to afford **1** as a white solid. The resulting powder was dissolved in 1 mL of THF-d_8 and the formation of H_2 was monitored by ^{1}H NMR spectra at 293 K.

In the same way, a sample of **1**-d_2 was prepared with $LiAlD_4$ (8.4 mg, 200 µmol), and the D_2 formation was monitored by ^{1}H NMR spectra at 25 °C.

The following figure shows the decay profiles of **1** and ***1-d*₂** at 293K and the respective fitting results.

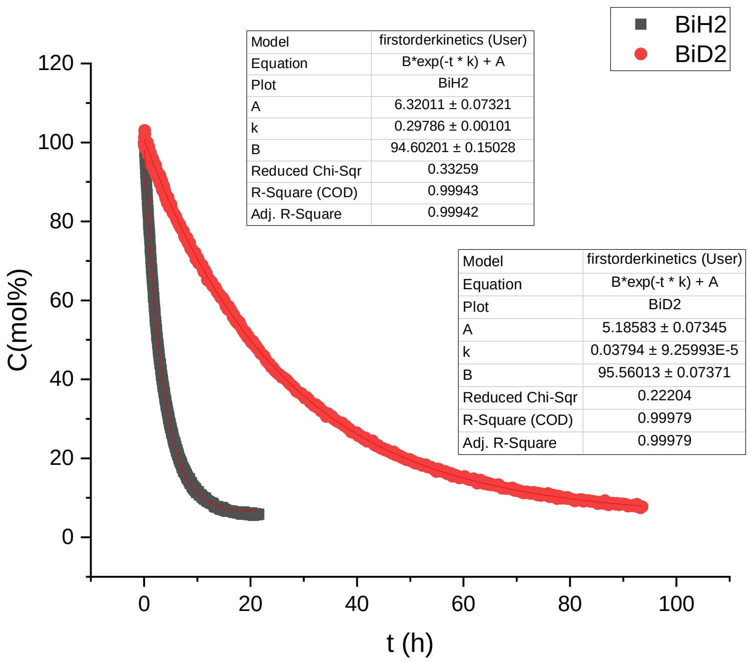

Figure S17. The decay profiles of **1** and **1-d_2** at 293K and the respective fitting results.

4.2.3. Eyring plot

Stock solution of $LiAlH_4$ was prepared: $LiAlH_4$ (72 μL, 1M in THF) diluted with THF-d_8 (0.93 mL).
A J. Young NMR tube was charged with complex **3** (8.0 mg, 7.2 μmol) and THF-d_8 (0.5 mL), and cooled to –78 °C. Under Ar, 100 μL $LiAlH_4$ stock solution was added. The reaction mixture was maintained at –78 °C, and quickly shaked several times before NMR studies at different temperatures.

An overview of the reaction profiles at different temperatures and the fitting results at the individual temperatures are shown in the following figures:

308 K

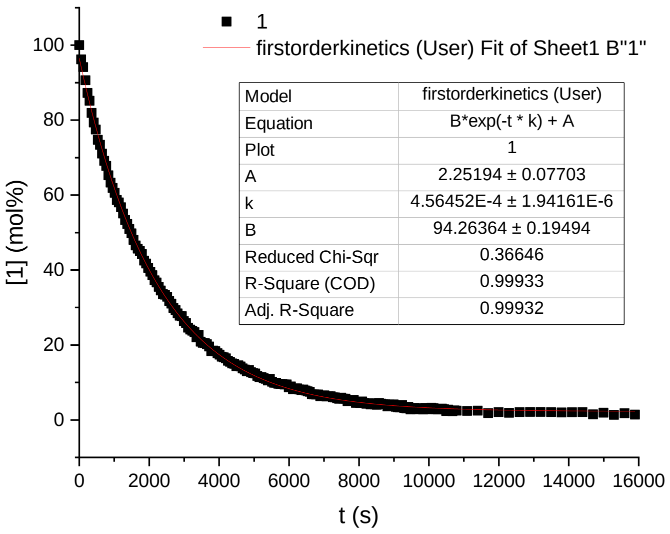

Figure S18. The reaction profiles and the fitting results at 308 K.

298 K

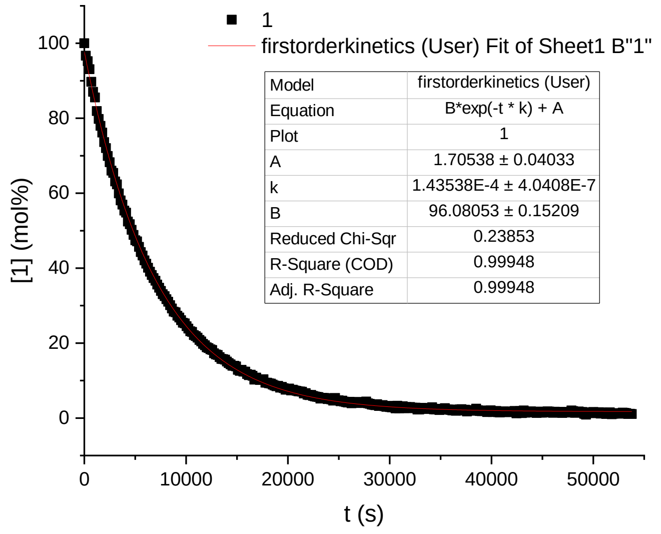

Figure S19. The reaction profiles and the fitting results at 298 K.

293 K

This sample was prepared according to the procedure reported in 4.2.2.

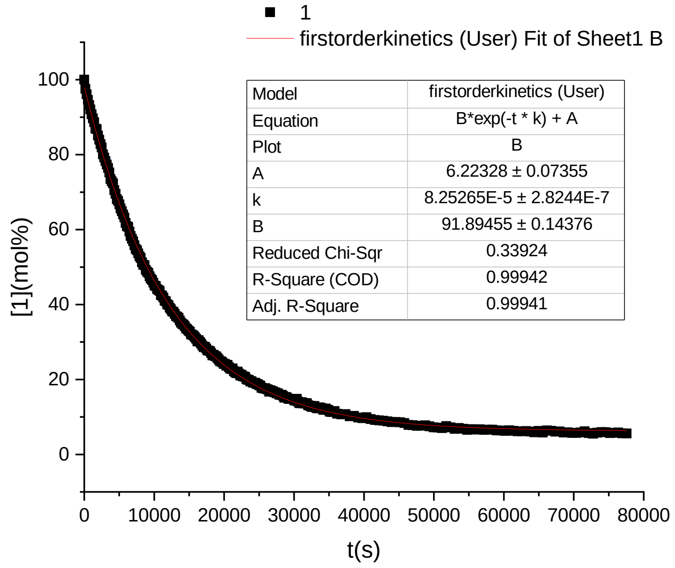

Figure S20. The reaction profiles and the fitting results at 293 K.

283 K

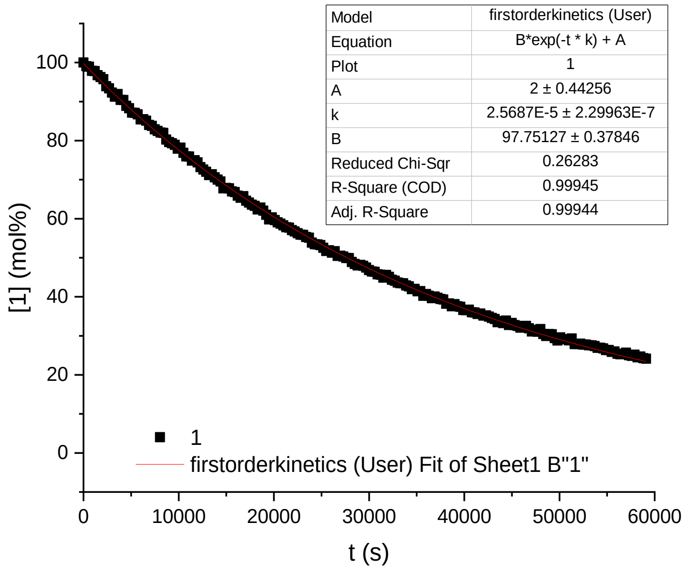

Figure S21. The reaction profiles and the fitting results at 283 K.

273 K

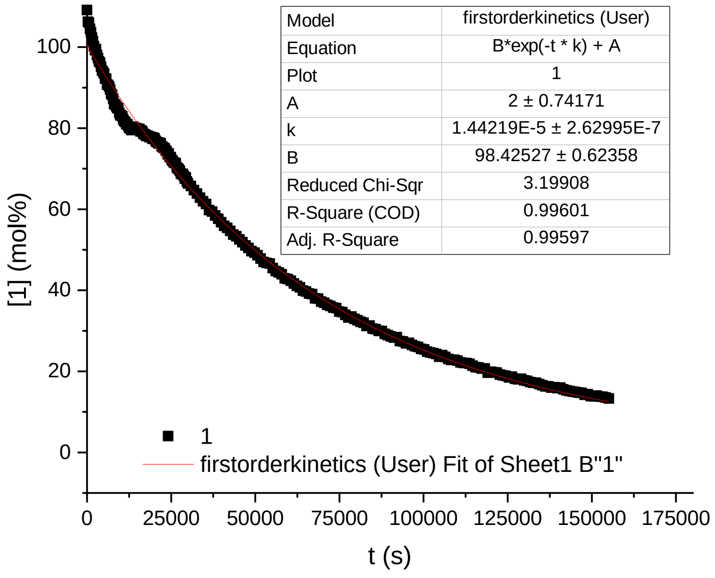

Figure S22. The reaction profiles and the fitting results at 273 K.

Table S1. A summary of all extracted values from the kinetic data used for the Eyring analysis.

#	*T* (K)	k_{obs} (s^{-1})
**1**	298.0	1.435E-04
**2**	283.0	2.569E-05
**3**	308.0	4.565E-04
**4**	278.0	1.442E-05
**5**	293.0	8.253E-05

Table S2. The physical constants used for the calculations.

	value	unit
R	8.3144598	J K^{-1} mol^{-1}
h	6.62607E-34	J s
k_B	1.38064852E-23	J K^{-1}

By plotting $\ln \frac{k \times \mathrm{h}}{\mathrm{k_b} \times T}$ against 1/T , the values of $-\Delta H^{‡}/R$ (slope) and $\Delta S^{‡}/R$ (intercept) can be obtained via linear regression using the LINEST function in Microsoft Excel.
Linear regression results:

Table S3. Linear regression results.

	$-\Delta H^{‡}$ / R	$\Delta S^{‡}$ / R	
Value	-9541.5049	-6.2480	Value
error	158.0215	0.5423	error
R^2	0.9992	0.0441	stdev(A)

These data were then used to derive the corresponding thermodynamic parameters ΔH‡, ΔS‡ and $\Delta G^{\ddagger}_{\mathrm{T}}$ (=ΔH‡-TΔS‡).

Table S4. The corresponding thermodynamic parameters ΔH‡, ΔS‡ and $\Delta G^{\ddagger}_{\mathrm{T}}$ (=ΔH‡-TΔS‡)

ΔH‡	79.3	± 1.3	kJ mol^{-1}
**ΔH‡**	18.9	± 0.3	**kcal mol^{-1}**
ΔS‡	-51.9	± 4.5	J mol^{-1} K^{-1}
**ΔS‡**	-12.4	± 1.1	**cal mol^{-1} K^{-1}**
ΔG‡ (25°C)	94.8	2.7	kJ mol^{-1}
**ΔG‡ (25°C)**	22.6	0.6	**kcal mol^{-1}**

The Eyring plot, including the linear regression results and the extracted values of the activation enthalpy ΔH‡, activation entropy ΔS‡ and Gibbs energy of activation $\Delta G^{\ddagger}_{25°\mathrm{C}}$, is shown in the following figure:

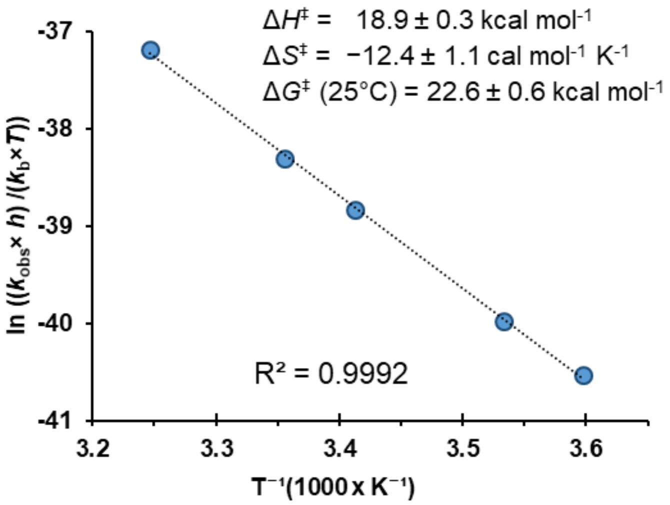

Figure S23. The reaction profiles and the fitting results at 308 K.

5. Reactivity Investigation of Organobismuth Dihydride

5.1. Reaction with N-Bromosuccinimide

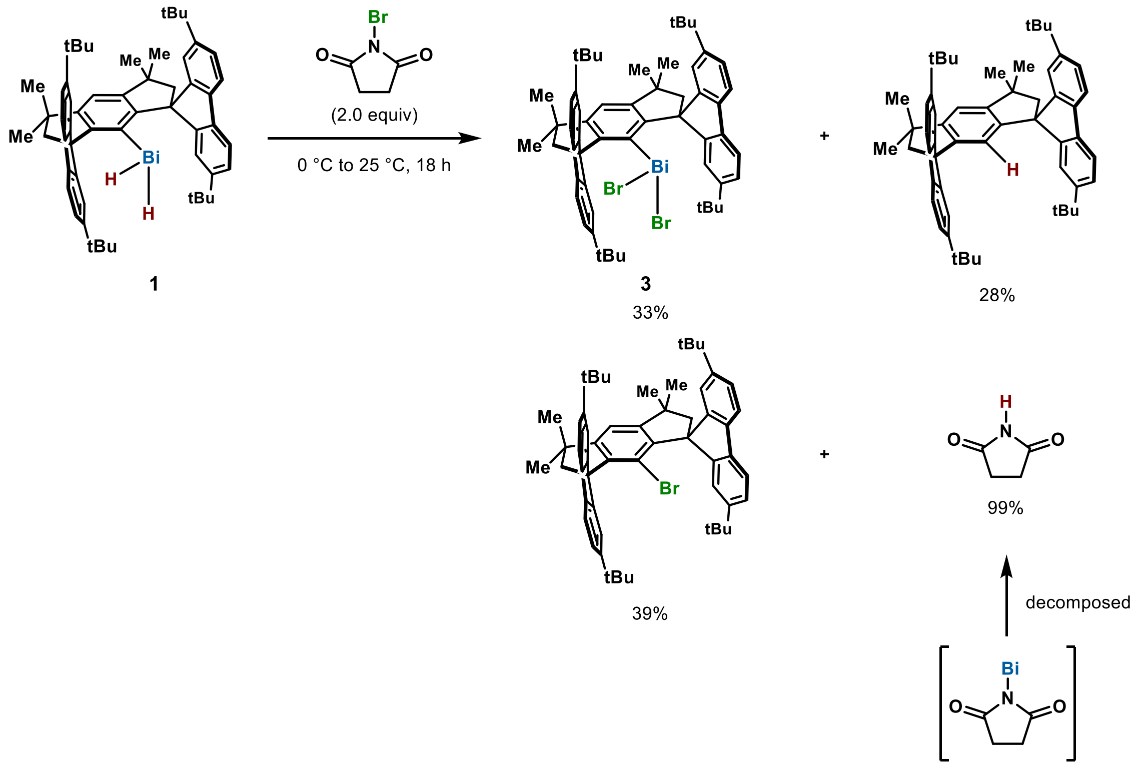

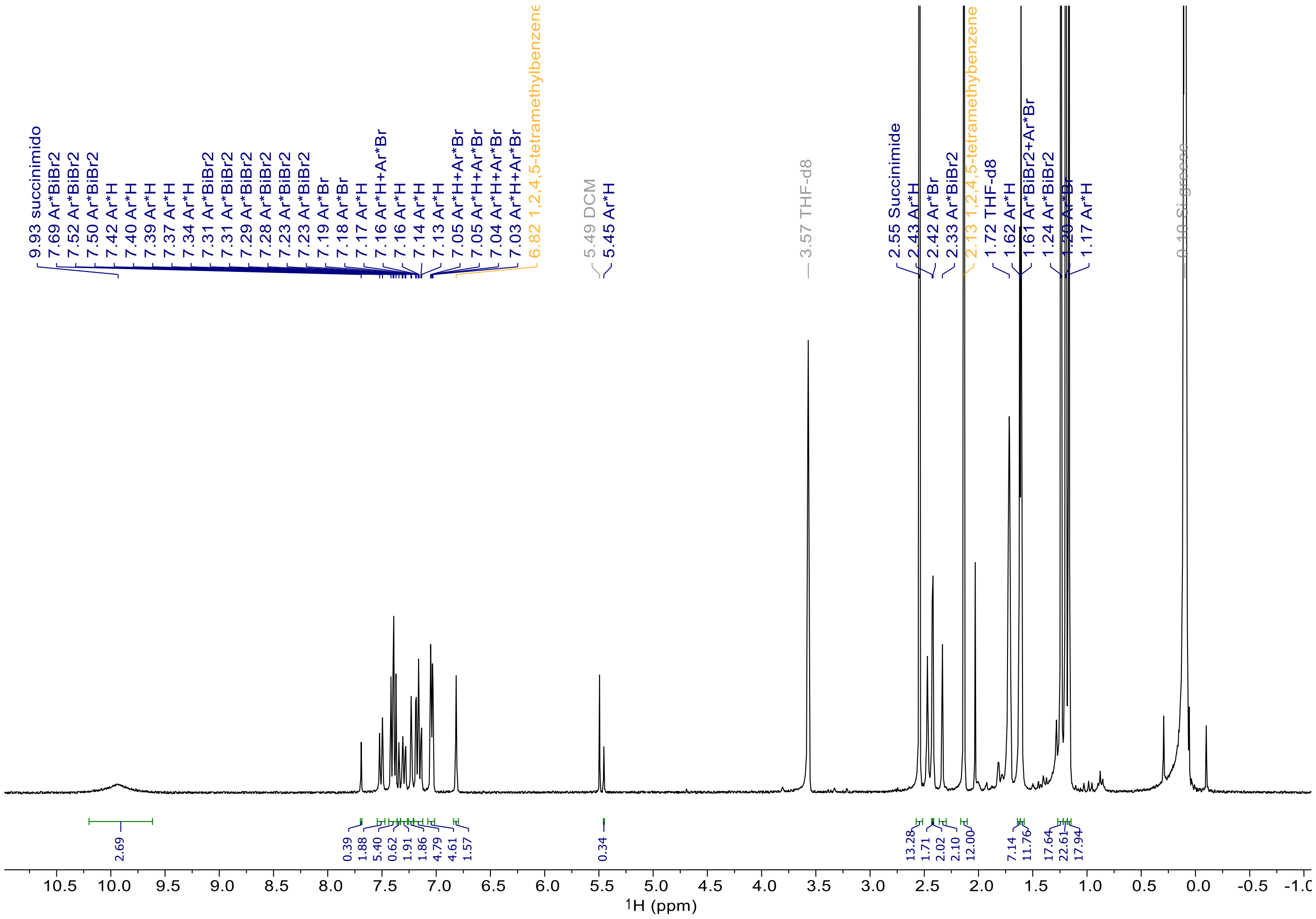

Figure S24. ^{1}H NMR spectrum of the reaction mixture of **1** and N-Bromosuccinimide, 300 MHz, THF-d_8, 223 K.

5.2. Unsuccessful Substrates

The reactivity of compound **1** with various organic substrates was investigated. Unfortunately, due to the facile formation of H_2 at low temperatures, the reactivity of compound **1** is likely restricted to temperatures below this threshold or fast processes to the H_2 formation. Unsuccessful substrates are shown below (5.2.1 to 5.2.3).

5.2.1. Attempt to Hydrobismuthation

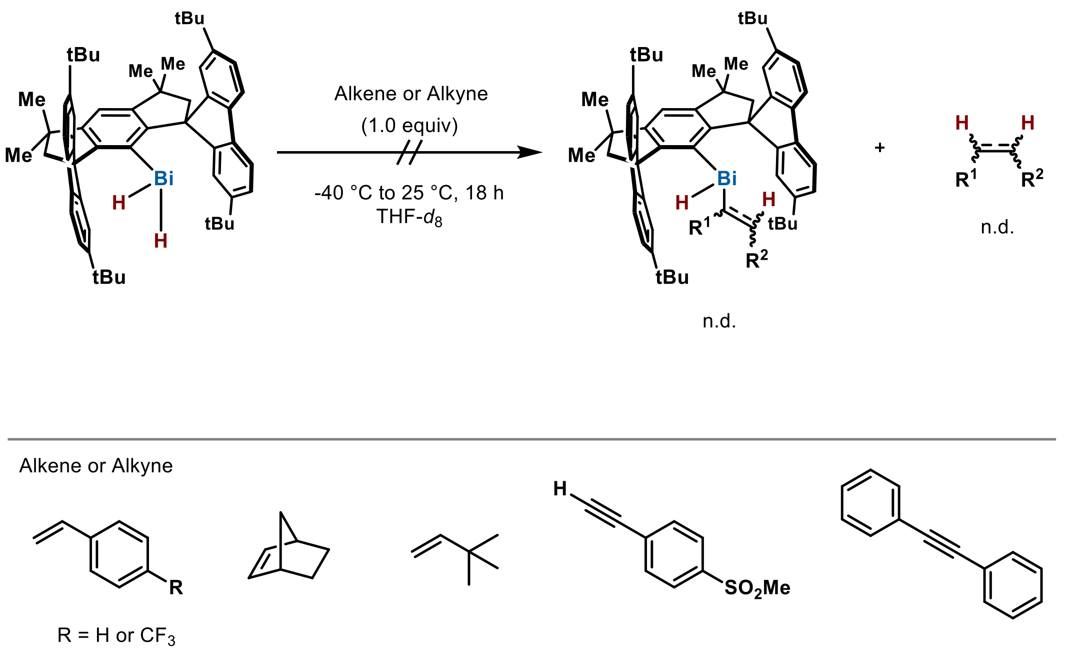

5.2.2. Attempt to Trapping of Hydrogen Radical

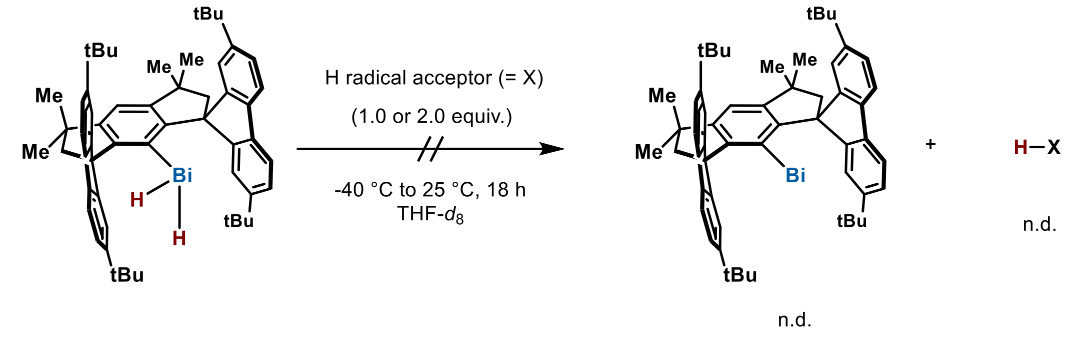

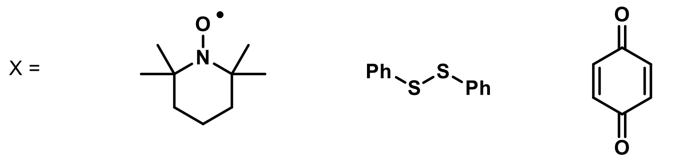

5.2.3. Attempt to the Reaction with Proton

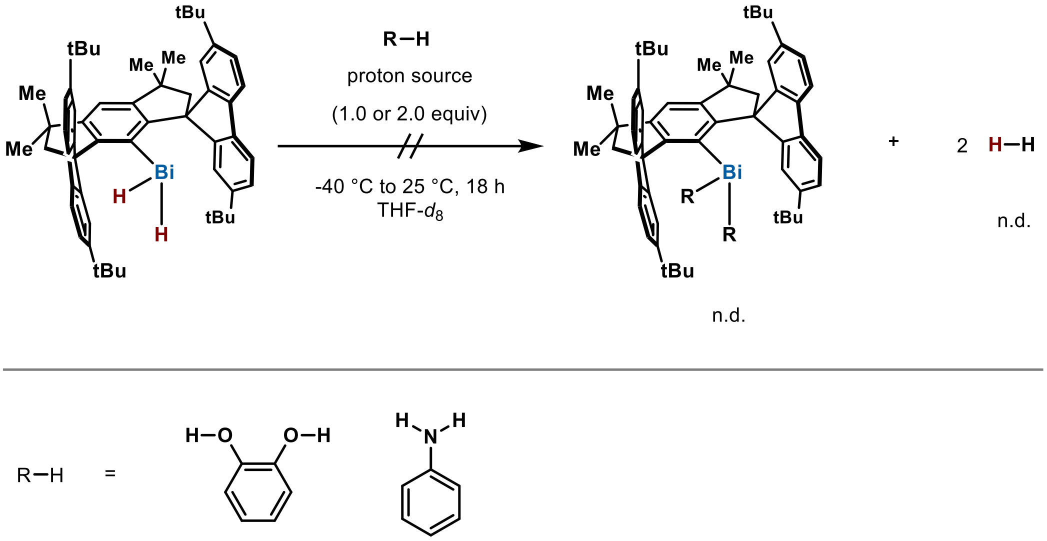

5.2.4. Attempt to the Reaction with Base

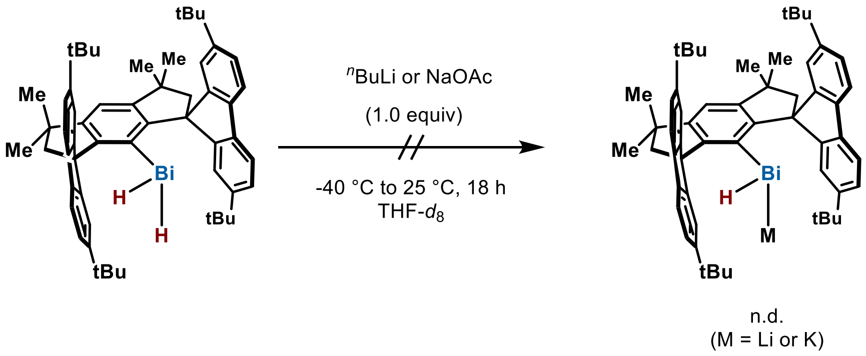

6. X-ray Crystal Structure Analysis of Organobismuth Dihydride

6.1 Single crystal structure analysis of Organobismuth Dihydride

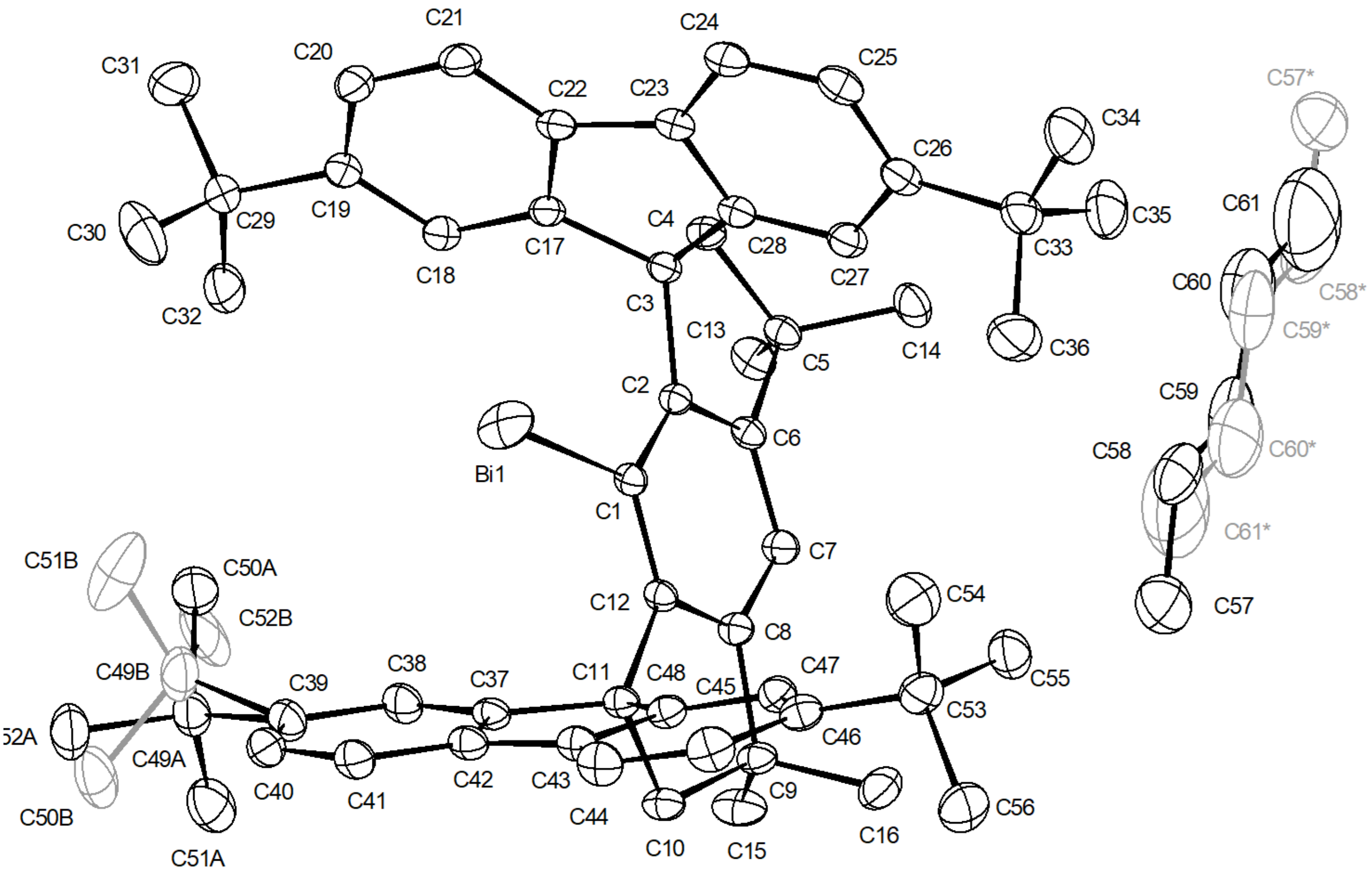

Figure S25. The molecular structure of ^{t}Bu-MsFluind-BiH_2 (1). H atoms have been removed for clarity.

Table S5. Crystal data and structure refinement.

Identification code	16139	
Empirical formula	$C_{117} H_{146} Bi_2$	
Color	colourless	
Formula weight	1970.424 g·mol^{-1}	
Temperature	100(2) K	
Wavelength	0.71073 Å	
Crystal system	Monoclinic	
Space group	$P2_1$/c, (no. 14)	
Unit cell dimensions	a = 14.8179(3) Å	α= 90°.
	b = 16.1857(3) Å	β= 99.094(1)°.
	c = 21.4312(4) Å	γ = 90°.
Volume	5075.41(17) Å^3	
Z	2	
Density (calculated)	1.289 Mg·m^{-3}	
Absorption coefficient	3.508 mm^{-1}	
F(000)	2015.695 e	
Crystal size	0.187 x 0.187 x 0.060 mm^3	
θ range for data collection	2.01 to 36.35°.	
Index ranges	-24 ≤ h ≤ 24, -26 ≤ k ≤ 26, -35 ≤ l ≤ 33	
Reflections collected	215461	
Independent reflections	24642 [R_{int} = 0.0695]	
Reflections with I>2σ(I)	18568	
Completeness to θ = 25.2417°	99.96 %	
Absorption correction	Gaussian	
Max. and min. transmission	0.88641 and 0.62734	
Refinement method	Full-matrix least-squares on F^2	
Data / restraints / parameters	24642 / 53 / 642	
Goodness-of-fit on F^2	1.0207	
Final R indices [I>2σ(I)]	R_1 = 0.0322	wR^2 = 0.0695
R indices (all data)	R_1 = 0.0544	wR^2 = 0.0775
Largest diff. peak and hole	1.4388 and -0.9388 e·Å^{-3}	

Table S6. Bond lengths [Å] and angles [°].

Bi(1)-Hh	1.8832(2)	Bi(1)-Hi	1.93376(18)
Bi(1)-C(1)	2.2648(14)	C(1)-C(2)	1.4042(18)
C(1)-C(12)	1.4039(19)	C(2)-C(3)	1.5263(19)
C(2)-C(6)	1.4031(19)	C(3)-C(4)	1.565(2)
C(3)-C(17)	1.529(2)	C(3)-C(28)	1.531(2)
C(4)-H(00a)	0.9900	C(4)-H(00b)	0.9900
C(4)-C(5)	1.548(2)	C(5)-C(6)	1.5212(19)
C(5)-C(13)	1.532(2)	C(5)-C(14)	1.536(2)
C(6)-C(7)	1.387(2)	C(7)-H(007)	0.9500
C(7)-C(8)	1.3926(19)	C(8)-C(9)	1.514(2)
C(8)-C(12)	1.4066(19)	C(9)-C(10)	1.547(2)
C(9)-C(15)	1.533(2)	C(9)-C(16)	1.536(2)
C(10)-H(00d)	0.9900	C(10)-H(00g)	0.9900
C(10)-C(11)	1.568(2)	C(11)-C(12)	1.5302(19)
C(11)-C(37)	1.526(2)	C(11)-C(48)	1.535(2)
C(13)-H(01a)	0.9800	C(13)-H(01b)	0.9800
C(13)-H(01c)	0.9800	C(14)-H(01g)	0.9800
C(14)-H(01h)	0.9800	C(14)-H(01i)	0.9800
C(15)-H(00h)	0.9800	C(15)-H(00i)	0.9800
C(15)-H(00j)	0.9800	C(16)-H(01d)	0.9800
C(16)-H(01e)	0.9800	C(16)-H(01f)	0.9800
C(17)-C(18)	1.392(2)	C(17)-C(22)	1.397(2)
C(18)-H(00L)	0.9500	C(18)-C(19)	1.403(2)
C(19)-C(20)	1.401(2)	C(19)-C(29)	1.537(2)
C(20)-H(010)	0.9500	C(20)-C(21)	1.388(2)
C(21)-H(00T)	0.9500	C(21)-C(22)	1.391(2)
C(22)-C(23)	1.466(2)	C(23)-C(24)	1.389(2)
C(23)-C(28)	1.398(2)	C(24)-H(00O)	0.9500
C(24)-C(25)	1.397(2)	C(25)-H(00Z)	0.9500
C(25)-C(26)	1.396(2)	C(26)-C(27)	1.403(2)
C(26)-C(33)	1.532(2)	C(27)-H(00Q)	0.9500
C(27)-C(28)	1.381(2)	C(29)-C(30)	1.534(3)
C(29)-C(31)	1.530(3)	C(29)-C(32)	1.529(3)
C(30)-H	0.9800	C(30)-H(01y)	0.9800
C(30)-Ha	0.9800	C(31)-H(01)	0.9800
C(31)-Hd	0.9800	C(31)-He	0.9800
C(32)-H(01j)	0.9800	C(32)-H(01k)	0.9800
C(32)-H(01l)	0.9800	C(33)-C(34)	1.519(3)
C(33)-C(35)	1.542(3)	C(33)-C(36)	1.533(3)
C(34)-H(01z)	0.9800	C(34)-Hb	0.9800

C(34)-Hc	0.9800	C(35)-H(01s)	0.9800
C(35)-H(01t)	0.9800	C(35)-H(01u)	0.9800
C(36)-H(1)	0.9800	C(36)-Hf	0.9800
C(36)-Hg	0.9800	C(37)-C(38)	1.380(2)
C(37)-C(42)	1.401(2)	C(38)-H(00E)	0.9500
C(38)-C(39)	1.406(2)	C(39)-C(40)	1.400(2)
C(39)-C(49A)	1.536(4)	C(39)-C(49B)	1.564(19)
C(40)-H(011)	0.9500	C(40)-C(41)	1.388(2)
C(41)-H(00C)	0.9500	C(41)-C(42)	1.392(2)
C(42)-C(43)	1.462(2)	C(43)-C(44)	1.388(2)
C(43)-C(48)	1.402(2)	C(44)-H(00R)	0.9500
C(44)-C(45)	1.380(2)	C(45)-H(015)	0.9500
C(45)-C(46)	1.403(2)	C(46)-C(47)	1.403(2)
C(46)-C(53)	1.533(2)	C(47)-H(00F)	0.9500
C(47)-C(48)	1.386(2)	C(53)-C(54)	1.540(3)
C(53)-C(55)	1.530(3)	C(53)-C(56)	1.537(2)
C(54)-H(01p)	0.9800	C(54)-H(01q)	0.9800
C(54)-H(01r)	0.9800	C(55)-H(01m)	0.9800
C(55)-H(01n)	0.9800	C(55)-H(01o)	0.9800
C(56)-H(01v)	0.9800	C(56)-H(01w)	0.9800
C(56)-H(01x)	0.9800	C(49A)-C(50A)	1.551(5)
C(49A)-C(51A)	1.539(5)	C(49A)-C(52A)	1.545(5)
C(50A)-H(4a)	0.9800	C(50A)-H(4b)	0.9800
C(50A)-H(4c)	0.9800	C(51A)-H(2a)	0.9800
C(51A)-H(2b)	0.9800	C(51A)-H(2c)	0.9800
C(52A)-H(3a)	0.9800	C(52A)-H(3b)	0.9800
C(52A)-H(3c)	0.9800	C(49B)-C(50B)	1.53(3)
C(49B)-C(51B)	1.56(3)	C(49B)-C(52B)	1.36(3)
C(50B)-H(13a)	0.9800	C(50B)-H(13b)	0.9800
C(50B)-H(13c)	0.9800	C(51B)-H(5a)	0.9800
C(51B)-H(5b)	0.9800	C(51B)-H(5c)	0.9800
C(52B)-H(7a)	0.9800	C(52B)-H(7b)	0.9800
C(52B)-H(7c)	0.9800	C(57)-H(12a)	0.9800
C(57)-H(12b)	0.9800	C(57)-H(12c)	0.9800
C(57)-C(58)	1.522(8)	C(58)-H(11a)	0.9900
C(58)-H(11b)	0.9900	C(58)-C(59)	1.526(8)
C(59)-H(10a)	0.9900	C(59)-H(10b)	0.9900
C(59)-C(60)	1.497(8)	C(60)-H(9a)	0.9900
C(60)-H(9b)	0.9900	C(60)-C(61)	1.494(11)
C(61)-H(8a)	0.9800	C(61)-H(8b)	0.9800
C(61)-H(8c)	0.9800		

Hi-Bi(1)-Hh	83.212(8)	C(1)-Bi(1)-Hh	97.84(4)
C(1)-Bi(1)-Hi	83.32(4)	C(2)-C(1)-Bi(1)	121.13(10)
C(12)-C(1)-Bi(1)	121.82(9)	C(12)-C(1)-C(2)	116.93(12)
C(3)-C(2)-C(1)	128.43(12)	C(6)-C(2)-C(1)	121.62(13)
C(6)-C(2)-C(3)	109.79(11)	C(4)-C(3)-C(2)	102.33(11)
C(17)-C(3)-C(2)	120.25(11)	C(17)-C(3)-C(4)	109.26(12)
C(28)-C(3)-C(2)	112.25(12)	C(28)-C(3)-C(4)	112.48(11)
C(28)-C(3)-C(17)	100.63(12)	H(00a)-C(4)-C(3)	110.08(8)
H(00b)-C(4)-C(3)	110.08(8)	H(00b)-C(4)-H(00a)	108.4
C(5)-C(4)-C(3)	108.12(11)	C(5)-C(4)-H(00a)	110.08(8)
C(5)-C(4)-H(00b)	110.08(8)	C(6)-C(5)-C(4)	102.02(11)
C(13)-C(5)-C(4)	110.58(13)	C(13)-C(5)-C(6)	111.43(12)
C(14)-C(5)-C(4)	112.67(13)	C(14)-C(5)-C(6)	112.22(12)
C(14)-C(5)-C(13)	107.91(12)	C(5)-C(6)-C(2)	112.98(12)
C(7)-C(6)-C(2)	120.68(12)	C(7)-C(6)-C(5)	126.32(13)
H(007)-C(7)-C(6)	120.65(8)	C(8)-C(7)-C(6)	118.70(13)
C(8)-C(7)-H(007)	120.65(8)	C(9)-C(8)-C(7)	126.15(13)
C(12)-C(8)-C(7)	120.68(13)	C(12)-C(8)-C(9)	113.15(12)
C(10)-C(9)-C(8)	101.94(11)	C(15)-C(9)-C(8)	111.84(12)
C(15)-C(9)-C(10)	110.70(13)	C(16)-C(9)-C(8)	111.40(13)
C(16)-C(9)-C(10)	112.32(13)	C(16)-C(9)-C(15)	108.59(14)
H(00d)-C(10)-C(9)	109.95(8)	H(00g)-C(10)-C(9)	109.95(8)
H(00g)-C(10)-H(00d)	108.3	C(11)-C(10)-C(9)	108.69(11)
C(11)-C(10)-H(00d)	109.95(8)	C(11)-C(10)-H(00g)	109.95(8)
C(12)-C(11)-C(10)	102.36(11)	C(37)-C(11)-C(10)	109.32(12)
C(37)-C(11)-C(12)	116.45(12)	C(48)-C(11)-C(10)	112.03(12)
C(48)-C(11)-C(12)	116.04(12)	C(48)-C(11)-C(37)	100.87(11)
C(8)-C(12)-C(1)	121.32(12)	C(11)-C(12)-C(1)	128.44(12)
C(11)-C(12)-C(8)	110.24(12)	H(01a)-C(13)-C(5)	109.5
H(01b)-C(13)-C(5)	109.5	H(01b)-C(13)-H(01a)	109.5
H(01c)-C(13)-C(5)	109.5	H(01c)-C(13)-H(01a)	109.5
H(01c)-C(13)-H(01b)	109.5	H(01g)-C(14)-C(5)	109.5
H(01h)-C(14)-C(5)	109.5	H(01h)-C(14)-H(01g)	109.5
H(01i)-C(14)-C(5)	109.5	H(01i)-C(14)-H(01g)	109.5
H(01i)-C(14)-H(01h)	109.5	H(00h)-C(15)-C(9)	109.5
H(00i)-C(15)-C(9)	109.5	H(00i)-C(15)-H(00h)	109.5
H(00j)-C(15)-C(9)	109.5	H(00j)-C(15)-H(00h)	109.5
H(00j)-C(15)-H(00i)	109.5	H(01d)-C(16)-C(9)	109.5
H(01e)-C(16)-C(9)	109.5	H(01e)-C(16)-H(01d)	109.5
H(01f)-C(16)-C(9)	109.5	H(01f)-C(16)-H(01d)	109.5
H(01f)-C(16)-H(01e)	109.5	C(18)-C(17)-C(3)	128.47(13)
C(22)-C(17)-C(3)	110.84(13)	C(22)-C(17)-C(18)	120.63(14)

H(00L)-C(18)-C(17)	120.08(9)	C(19)-C(18)-C(17)	119.83(14)
C(19)-C(18)-H(00L)	120.08(9)	C(20)-C(19)-C(18)	118.50(14)
C(29)-C(19)-C(18)	122.86(14)	C(29)-C(19)-C(20)	118.62(14)
H(010)-C(20)-C(19)	119.04(9)	C(21)-C(20)-C(19)	121.93(15)
C(21)-C(20)-H(010)	119.04(9)	H(00T)-C(21)-C(20)	120.55(9)
C(22)-C(21)-C(20)	118.91(14)	C(22)-C(21)-H(00T)	120.55(9)
C(21)-C(22)-C(17)	120.17(14)	C(23)-C(22)-C(17)	108.62(13)
C(23)-C(22)-C(21)	131.14(14)	C(24)-C(23)-C(22)	131.35(14)
C(28)-C(23)-C(22)	108.48(13)	C(28)-C(23)-C(24)	120.08(14)
H(00O)-C(24)-C(23)	120.62(10)	C(25)-C(24)-C(23)	118.76(15)
C(25)-C(24)-H(00O)	120.62(9)	H(00Z)-C(25)-C(24)	119.07(9)
C(26)-C(25)-C(24)	121.86(15)	C(26)-C(25)-H(00Z)	119.07(9)
C(27)-C(26)-C(25)	118.14(15)	C(33)-C(26)-C(25)	122.82(15)
C(33)-C(26)-C(27)	119.00(15)	H(00Q)-C(27)-C(26)	119.75(10)
C(28)-C(27)-C(26)	120.50(15)	C(28)-C(27)-H(00Q)	119.75(9)
C(23)-C(28)-C(3)	110.74(13)	C(27)-C(28)-C(3)	128.77(13)
C(27)-C(28)-C(23)	120.46(14)	C(30)-C(29)-C(19)	108.57(15)
C(31)-C(29)-C(19)	109.99(14)	C(31)-C(29)-C(30)	109.42(18)
C(32)-C(29)-C(19)	112.48(14)	C(32)-C(29)-C(30)	108.53(15)
C(32)-C(29)-C(31)	107.81(17)	H-C(30)-C(29)	109.5
H(01y)-C(30)-C(29)	109.5	H(01y)-C(30)-H	109.5
Ha-C(30)-C(29)	109.5	Ha-C(30)-H	109.5
Ha-C(30)-H(01y)	109.5	H(01)-C(31)-C(29)	109.5
Hd-C(31)-C(29)	109.5	Hd-C(31)-H(01)	109.5
He-C(31)-C(29)	109.5	He-C(31)-H(01)	109.5
He-C(31)-Hd	109.5	H(01j)-C(32)-C(29)	109.5
H(01k)-C(32)-C(29)	109.5	H(01k)-C(32)-H(01j)	109.5
H(01l)-C(32)-C(29)	109.5	H(01l)-C(32)-H(01j)	109.5
H(01l)-C(32)-H(01k)	109.5	C(34)-C(33)-C(26)	112.73(16)
C(35)-C(33)-C(26)	109.69(15)	C(35)-C(33)-C(34)	107.92(15)
C(36)-C(33)-C(26)	108.42(15)	C(36)-C(33)-C(34)	108.50(17)
C(36)-C(33)-C(35)	109.54(19)	H(01z)-C(34)-C(33)	109.5
Hb-C(34)-C(33)	109.5	Hb-C(34)-H(01z)	109.5
Hc-C(34)-C(33)	109.5	Hc-C(34)-H(01z)	109.5
Hc-C(34)-Hb	109.5	H(01s)-C(35)-C(33)	109.5
H(01t)-C(35)-C(33)	109.5	H(01t)-C(35)-H(01s)	109.5
H(01u)-C(35)-C(33)	109.5	H(01u)-C(35)-H(01s)	109.5
H(01u)-C(35)-H(01t)	109.5	H(1)-C(36)-C(33)	109.5
Hf-C(36)-C(33)	109.5	Hf-C(36)-H(1)	109.5
Hg-C(36)-C(33)	109.5	Hg-C(36)-H(1)	109.5
Hg-C(36)-Hf	109.5	C(38)-C(37)-C(11)	128.56(13)
C(42)-C(37)-C(11)	110.92(13)	C(42)-C(37)-C(38)	120.48(13)

H(00E)-C(38)-C(37)	119.77(9)	C(39)-C(38)-C(37)	120.46(14)
C(39)-C(38)-H(00E)	119.77(10)	C(40)-C(39)-C(38)	117.92(15)
C(49A)-C(39)-C(38)	118.74(19)	C(49A)-C(39)-C(40)	123.27(19)
C(49B)-C(39)-C(38)	124.6(7)	C(49B)-C(39)-C(40)	114.5(7)
C(49B)-C(39)-C(49A)	20.8(7)	H(011)-C(40)-C(39)	118.83(10)
C(41)-C(40)-C(39)	122.34(14)	C(41)-C(40)-H(011)	118.83(9)
H(00C)-C(41)-C(40)	120.70(9)	C(42)-C(41)-C(40)	118.60(14)
C(42)-C(41)-H(00C)	120.70(9)	C(41)-C(42)-C(37)	120.18(14)
C(43)-C(42)-C(37)	108.35(13)	C(43)-C(42)-C(41)	131.37(14)
C(44)-C(43)-C(42)	130.66(14)	C(48)-C(43)-C(42)	108.99(13)
C(48)-C(43)-C(44)	120.24(15)	H(00R)-C(44)-C(43)	120.42(9)
C(45)-C(44)-C(43)	119.17(14)	C(45)-C(44)-H(00R)	120.42(9)
H(015)-C(45)-C(44)	119.10(9)	C(46)-C(45)-C(44)	121.81(15)
C(46)-C(45)-H(015)	119.10(10)	C(47)-C(46)-C(45)	118.37(15)
C(53)-C(46)-C(45)	118.90(14)	C(53)-C(46)-C(47)	122.73(14)
H(00F)-C(47)-C(46)	119.90(9)	C(48)-C(47)-C(46)	120.20(14)
C(48)-C(47)-H(00F)	119.90(8)	C(43)-C(48)-C(11)	110.10(13)
C(47)-C(48)-C(11)	129.78(13)	C(47)-C(48)-C(43)	120.12(14)
C(54)-C(53)-C(46)	109.24(15)	C(55)-C(53)-C(46)	112.36(14)
C(55)-C(53)-C(54)	108.28(16)	C(56)-C(53)-C(46)	109.04(14)
C(56)-C(53)-C(54)	109.65(16)	C(56)-C(53)-C(55)	108.25(15)
H(01p)-C(54)-C(53)	109.5	H(01q)-C(54)-C(53)	109.5
H(01q)-C(54)-H(01p)	109.5	H(01r)-C(54)-C(53)	109.5
H(01r)-C(54)-H(01p)	109.5	H(01r)-C(54)-H(01q)	109.5
H(01m)-C(55)-C(53)	109.5	H(01n)-C(55)-C(53)	109.5
H(01n)-C(55)-H(01m)	109.5	H(01o)-C(55)-C(53)	109.5
H(01o)-C(55)-H(01m)	109.5	H(01o)-C(55)-H(01n)	109.5
H(01v)-C(56)-C(53)	109.5	H(01w)-C(56)-C(53)	109.5
H(01w)-C(56)-H(01v)	109.5	H(01x)-C(56)-C(53)	109.5
H(01x)-C(56)-H(01v)	109.5	H(01x)-C(56)-H(01w)	109.5
C(50A)-C(49A)-C(39)	107.6(3)	C(51A)-C(49A)-C(39)	110.7(2)
C(51A)-C(49A)-C(50A)	109.9(3)	C(52A)-C(49A)-C(39)	112.5(3)
C(52A)-C(49A)-C(50A)	107.5(3)	C(52A)-C(49A)-C(51A)	108.6(3)
H(4a)-C(50A)-C(49A)	109.5	H(4b)-C(50A)-C(49A)	109.5
H(4b)-C(50A)-H(4a)	109.5	H(4c)-C(50A)-C(49A)	109.5
H(4c)-C(50A)-H(4a)	109.5	H(4c)-C(50A)-H(4b)	109.5
H(2a)-C(51A)-C(49A)	109.5	H(2b)-C(51A)-C(49A)	109.5
H(2b)-C(51A)-H(2a)	109.5	H(2c)-C(51A)-C(49A)	109.5
H(2c)-C(51A)-H(2a)	109.5	H(2c)-C(51A)-H(2b)	109.5
H(3a)-C(52A)-C(49A)	109.5	H(3b)-C(52A)-C(49A)	109.5
H(3b)-C(52A)-H(3a)	109.5	H(3c)-C(52A)-C(49A)	109.5
H(3c)-C(52A)-H(3a)	109.5	H(3c)-C(52A)-H(3b)	109.5

C(50B)-C(49B)-C(39)	103.7(13)	C(51B)-C(49B)-C(39)	113.7(15)
C(51B)-C(49B)-C(50B)	107.4(15)	C(52B)-C(49B)-C(39)	113.5(15)
C(52B)-C(49B)-C(50B)	113(2)	C(52B)-C(49B)-C(51B)	105.5(18)
H(13a)-C(50B)-C(49B)	109.5	H(13b)-C(50B)-C(49B)	109.5
H(13b)-C(50B)-H(13a)	109.5	H(13c)-C(50B)-C(49B)	109.5
H(13c)-C(50B)-H(13a)	109.5	H(13c)-C(50B)-H(13b)	109.5
H(5a)-C(51B)-C(49B)	109.5	H(5b)-C(51B)-C(49B)	109.5
H(5b)-C(51B)-H(5a)	109.5	H(5c)-C(51B)-C(49B)	109.5
H(5c)-C(51B)-H(5a)	109.5	H(5c)-C(51B)-H(5b)	109.5
H(7a)-C(52B)-C(49B)	109.5	H(7b)-C(52B)-C(49B)	109.5
H(7b)-C(52B)-H(7a)	109.5	H(7c)-C(52B)-C(49B)	109.5
H(7c)-C(52B)-H(7a)	109.5	H(7c)-C(52B)-H(7b)	109.5
H(12b)-C(57)-H(12a)	109.5	H(12c)-C(57)-H(12a)	109.5
H(12c)-C(57)-H(12b)	109.5	C(58)-C(57)-H(12a)	109.5
C(58)-C(57)-H(12b)	109.5	C(58)-C(57)-H(12c)	109.5
H(11a)-C(58)-C(57)	108.1(4)	H(11b)-C(58)-C(57)	108.1(3)
H(11b)-C(58)-H(11a)	107.3	C(59)-C(58)-C(57)	116.8(7)
C(59)-C(58)-H(11a)	108.1(6)	C(59)-C(58)-H(11b)	108.1(6)
H(10a)-C(59)-C(58)	108.4(6)	H(10b)-C(59)-C(58)	108.4(6)
H(10b)-C(59)-H(10a)	107.46587372(3)	C(60)-C(59)-C(58)	115.4(6)
C(60)-C(59)-H(10a)	108.4(3)	C(60)-C(59)-H(10b)	108.4(4)
H(9a)-C(60)-C(59)	106.7(3)	H(9b)-C(60)-C(59)	106.7(4)
H(9b)-C(60)-H(9a)	106.6	C(61)-C(60)-C(59)	122.5(12)
C(61)-C(60)-H(9a)	106.7(8)	C(61)-C(60)-H(9b)	106.7(9)
H(8a)-C(61)-C(60)	109.5	H(8b)-C(61)-C(60)	109.5
H(8b)-C(61)-H(8a)	109.5	H(8c)-C(61)-C(60)	109.5
H(8c)-C(61)-H(8a)	109.5	H(8c)-C(61)-H(8b)	109.5

6.2. Refinement details

The structure was solved by the standard method (SHELXT) and refined (OLEX2 refine). One reflection (OMIT 1 1 0) was probably affected by the beam stop and was excluded before the final refinement cycles. The solute was modelled using the "pentane" fragment from the DSR tool as an Olex2 plugin.[2,3] After describing the disordered ^{t}Bu group and the solvent, the residual electron density map shown could be found.

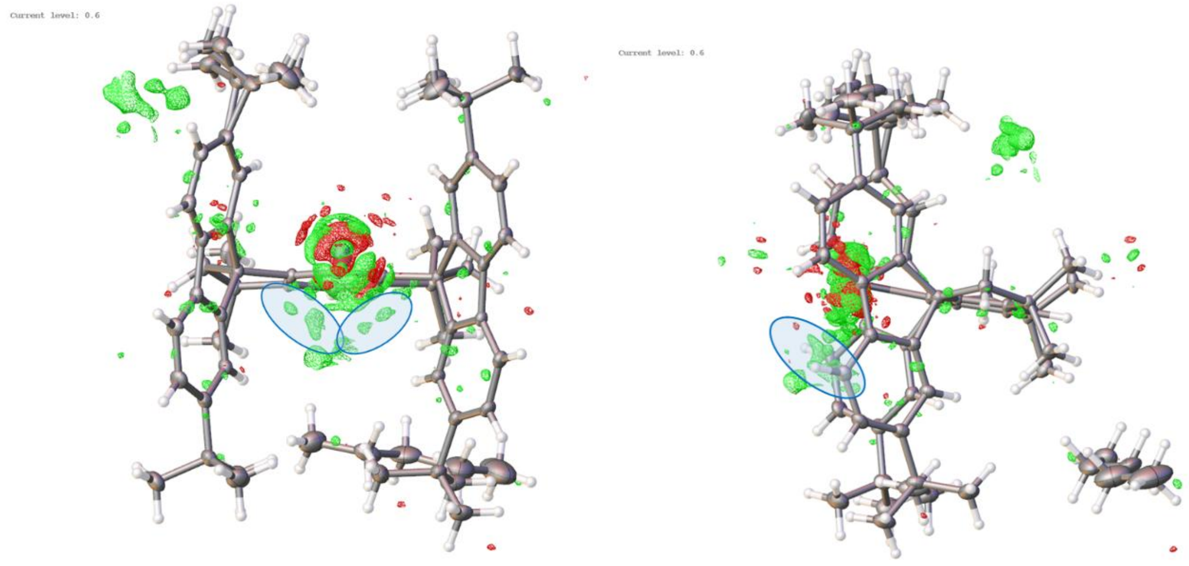

Figure S26. Structure and difference density distribution of compound M^{s}Fluind-BiH_2 · Pentane (source: Olex2, diff-map, level 0.6 e/$Å^3$, 0.1 Å resolution) from different orientations, slightly indicating the presence of H atoms near the central Bi atom (blue cycles) and sights of anharmonic motions.

Two interesting features can be seen in the vicinity of the Bi-atom. The first is the shashlik-like (alternating) arrangement of the positive and negative residual electron densities. This is probably due to the anharmonic motion of the heavy atom.

Secondly, positive residual electron densities are found at a distance of about 1.8 Å to the Bi atom. This possibly indicates the potential position of the H atoms. The dihydride model was used for this purpose. However, direct identification from the SC-XRD data is not possible. The presence of H atoms attached to the central Bi atom was confirmed by IR and ^{1}H NMR. Further evidence for the presence of BiH_2 is derived indirectly from the geometry of the M^{s}Fluind ligand (see geometrical aspects section). In addition, the coordinates of the two H atoms on Bi must be fixed during refinement, otherwise no convergence can be achieved. For this reason, no precise statement can be made about the BiH_2 bonding geometry herein. The feasibility of conducting neutron diffraction studies is currently being evaluated.

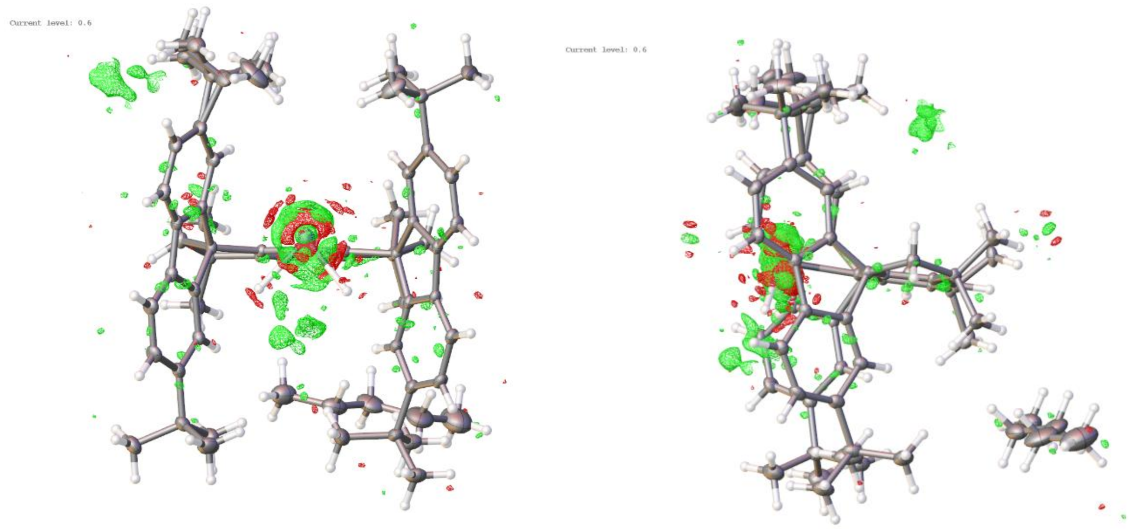

Figure S27. Structure and difference density distribution of compound M^{s}FluindBiH_2 · Pentane (source: Olex2, diff-map, level 0.6 e/$Å^3$, 0.1 Å resolution) after insertion of two fixed H atoms from different orientations.

High residual density peaks (Q1 = 9.03 e/Å^3) are found in the vicinity of the heavy Bi central atom (0.673 Å away from Bi1). It appears as a shashlik-like distribution, suggesting that anharmonic motion could be responsible for this observation. To account for this, higher order *Gram-Charlier* coefficients were applied before the final refinement cycles. For the final refinement, the anis -a (anharmonic motion refinement) command in the Olex2 refinement was applied to the Bi central atom. This applies higher *Gram-Charlier* coefficients to the refinement, taking into account the anharmonic motion of the central atom. This does not significantly affect the reflection to parameter ratio.

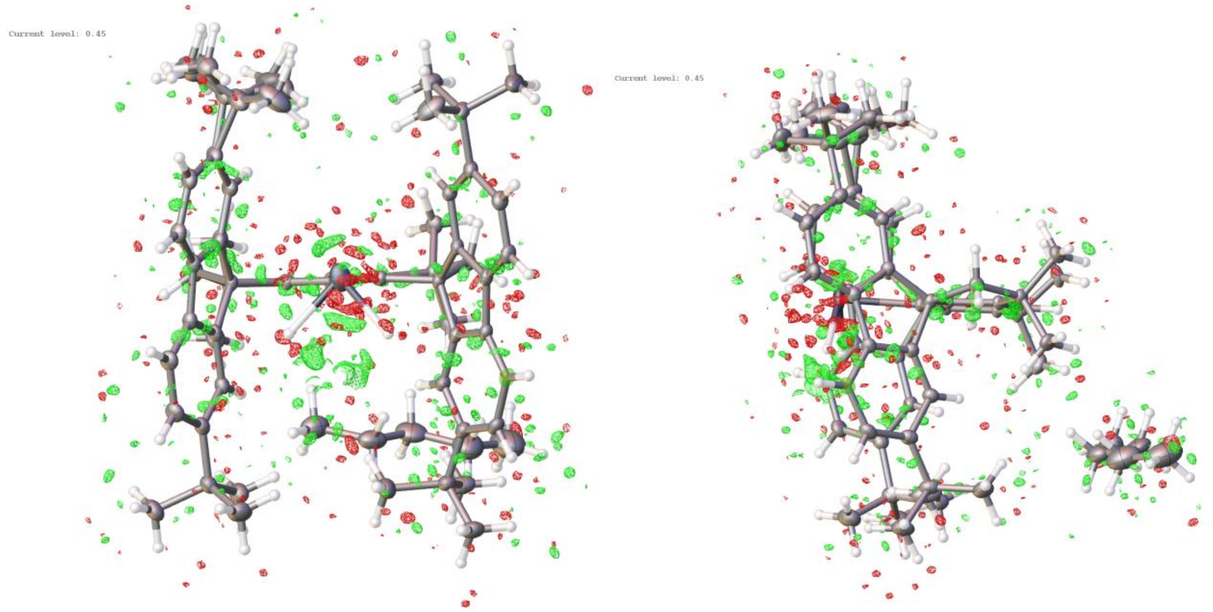

Figure S28. Structure and difference density distribution of compound M^{s}Fluind-BiH_2 · Pentane (source: Olex2, diff-map, level 0.45 e/Å^3, 0.1 Å resolution) from different orientations, after applying the *Gramm-Charlier* anharmonic displacement correction to the Bi atom.

The application of *Gramm-Charlier* coefficients reduce the residual density (Q2 = 1.11 e/Å^3, 2.624 Å, Q3 = 0.77 e/Å^3, 0.923 Å away from Bi1) in the heavy atom regimen to an acceptable value.
According to the VSEPR model (Valence shell electron pair repulsion) the central Bi atom is a AX_3E_1 type with a trigonal pyramidal geometry as many other pnictogen compounds.

6.3. Database search

A database (WebCSD Version 1.9.61, https://www.ccdc.cam.ac.uk/structures/WebCSD/StructureSearch) survey was performed on 25th of March 2025 to search for related structural motives. Several search motive was used.

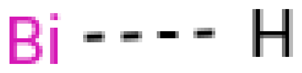

Figure S29. Search motif for data survey on Bi-H complexes.

Surprisingly, only one hit was found during the search. This is a structure with the Ref. code WIYQAW (CCDC No. 143880) and represents a Bi monohydride.

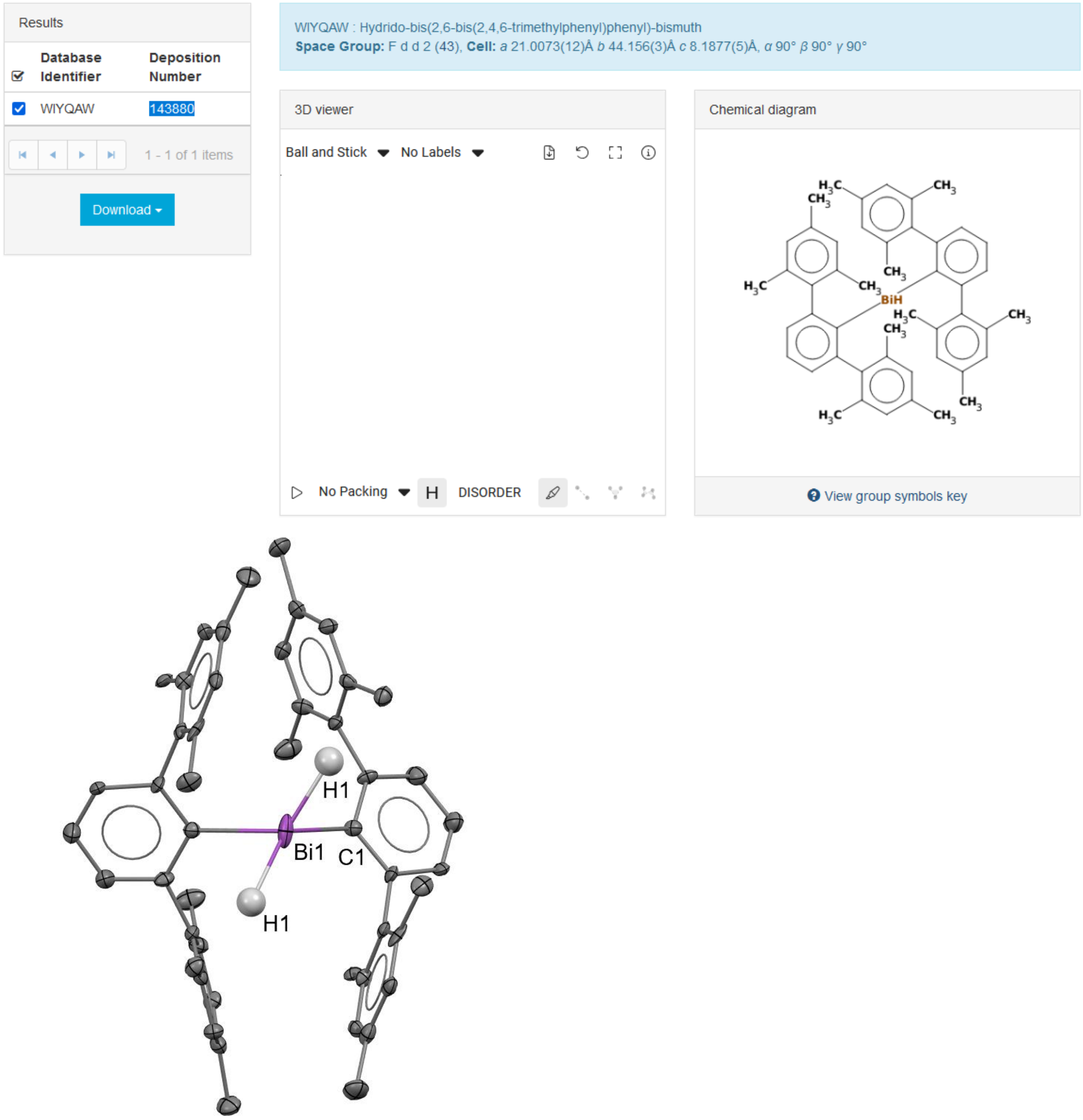

Figure S30. Results from the database search (top) and visualisation of the WIYQAW structure (bottom).

The molecule crystallises in a orthrhombic structure (space group *Fdd*2, a = 21.0073(12) Å, b= 44.156(3) Å, c = 8.1877(5) Å) with 8 molecules in the unit cell. The molecule is located on a special position (twofold axis of rotation). Selected bond lengths (Å) and angles (°) are as follows: Bi(1)-C(1) 2.314(7), Bi(1)-H(1) 1.94(2); C(1)-Bi- C(1A) 114.9(3).

6.4. Geometrical aspects

Since the structure refinement of the SC-XRD data does not provide very clear evidence for the presence of H atoms in the coordination sphere of the Bi atom (unlike the previously studied Sb-H_2), their presence is indirectly derived from geometric comparisons of related structures. For this purpose, the already known structures of the M^{s}Fluind-SbH_2 (Ref. code WIJQOZ, CCDC No. 2239605) and the monovalent M^{s}Fluind-Bi(I) (Ref. code GEZGUR, CCDC No. 2225002) are used.

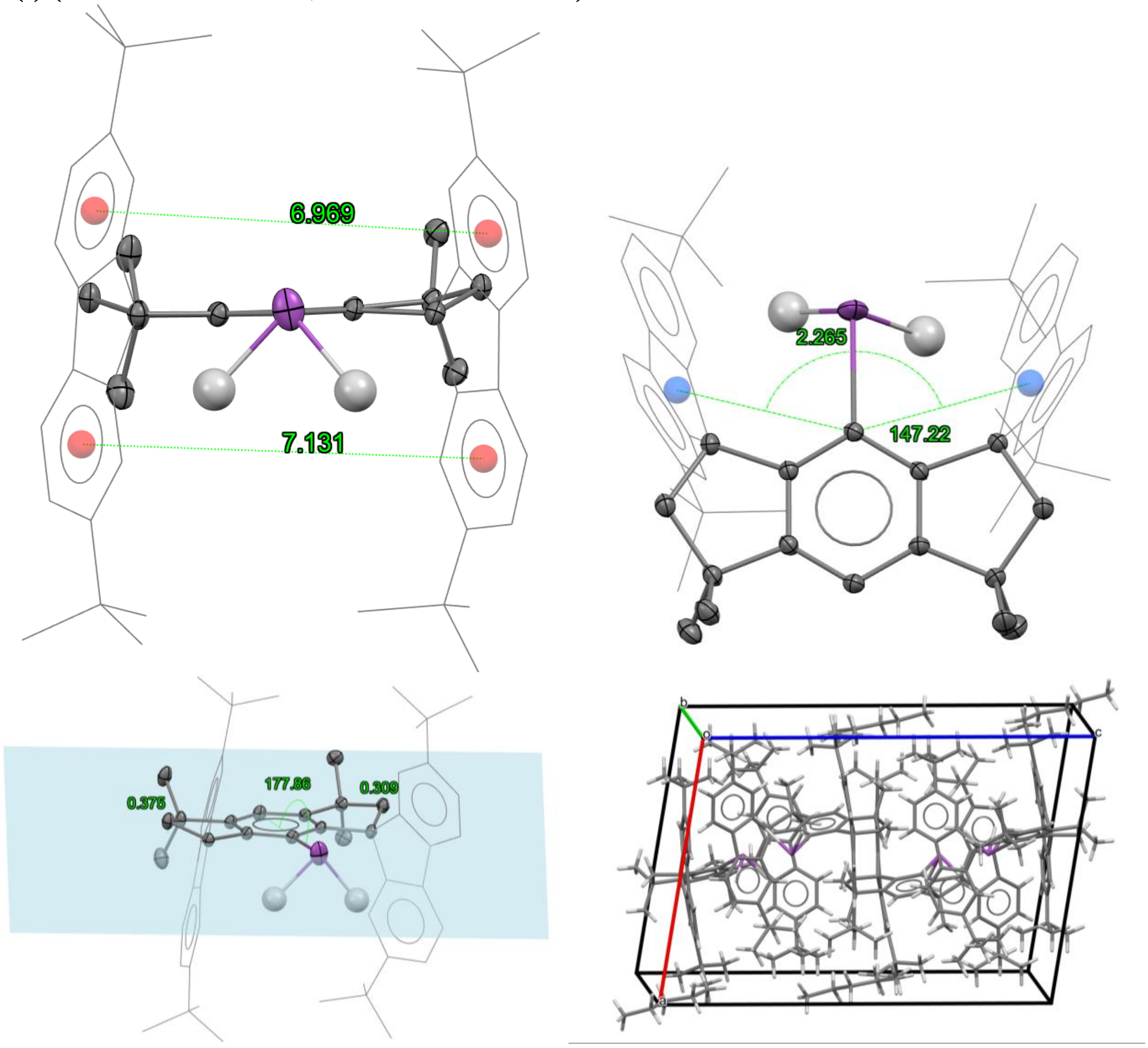

Figure S31. Geometrical aspects of the M^{s}Fluind-BiH_2 structure. Centroid distances of the flanking units of the M^{s}Fluind ligand (top left), centroid-C1-centroid opening angle and Bi1-C1 distance (top right), aryl plane with distortion of the C atoms and C_{ipso}-C1-Bi1 angle (bottom left) and packing approximately along the crystallographic b-axis.

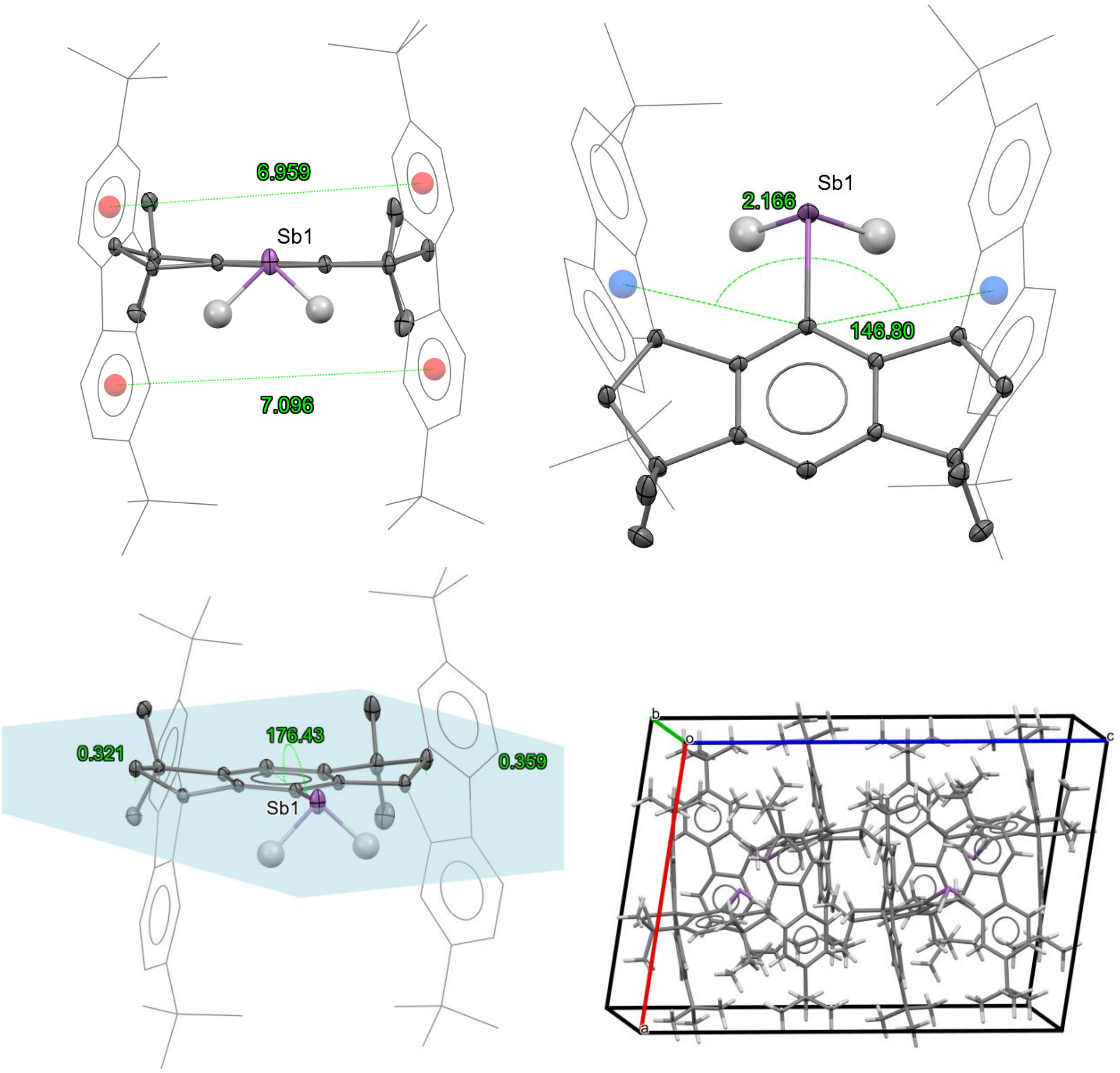

Figure S32. Geometrical aspects of the M^{s}Fluind-SbH_2 structure. Centroid distances of the flanking units of the M^{s}Fluind ligand (top left), centroid-C1-centroid opening angle and Sb1-C1 distance (top right), aryl plane with distortion of the C atoms and C_{ipso}-C1-Sb1 angle (bottom left) and packing approximately along the crystallographic b-axis.

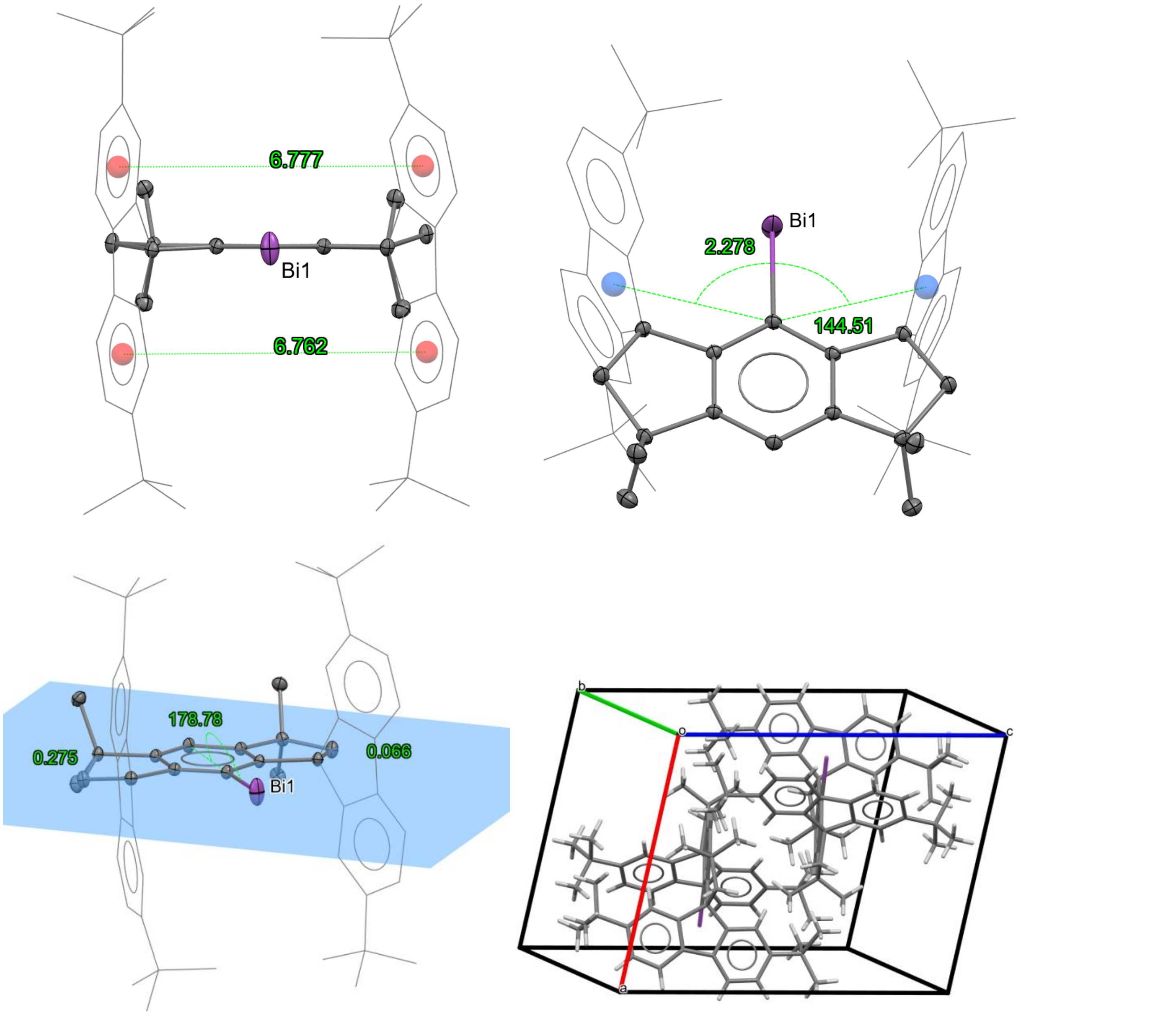

Figure S33. Geometrical aspects of the M^{s}Fluind-Bi(I) structure. Centroid distances of the flanking units of the M^{s}Fluind ligand (top left), centroid-C1-centroid opening angle and Bi1-C1 distance (top right), aryl plane with distortion of the C atoms and C_{ipso}-C1-Bi1 angle (bottom left) and packing approximately along the crystallographic b-axis.

Our previous work with the M^{s}Fluind ligand scaffold has already shown that it exhibits a certain degree of flexibility.
If we look at the distances between the centroids of the flanking aryl rings, we see that they are 6.969 Å and 7.131 Å apart. In particular, the greater distance on the side on which the H atoms are assumed to be present speaks in favour of their presence. A similar behaviour can be seen in the M^{s}Fluind-SbH_2 structure, where the distances are 6.959 Å and 7.096 Å. In M^{s}Fluind-Bi(I), however, the distances are almost the identical (6.777 Å and 6.762 Å), suggesting a parallel arrangement of the flanks.
The opening/stretching angle of the M^{s}Fluind ligand provides a further indication of the presence of the H atoms on Bi. This was determined between the centroids of the five-membered rings of the flanks and C1. In the M^{s}Fluind-BiH_2 structure this is 147.22° and is the largest in comparison.
A further indication of the flexibility of the M^{s}Fluind ligand can be obtained by observing the plane of the back six membered aryl ring. In the M^{s}Fluind-Bi-H_2 structure, the two C atoms of the five-membered at the backside rings are slightly out of plane (plane C distances are 0.375 Å and 0.309 Å). The angle between Bi-C1-C_{ipso} is 177.86° and shows an almost planar entity.

There are also strong similarities in packing and structure between M^{s}Fluind-BiH_2 and M^{s}Fluind-SbH_2. Both structures crystallise in the monoclinic space group *P*2$_1$/*c* with two molecules per unit cell. M^{s}Fluind-Bi(I) on the other hand crystallises in a triclinic form with two molecules per unit cell. The lattice constants and volumes of the two dihydride structures (M^{s}Fluind-BiH_2 and M^{s}Fluind-SbH_2) are also very similar. A slight increase in the lattice constants can be observed in M^{s}Fluind-BiH_2, which is related to the larger atomic radius of Bi compared to Sb.

Table S7. Overview of the lattice parameters of the three M^{s}Fluind structures described.

	**M^{s}Fluind-BiH$_2$**	**M^{s}Fluind-SbH$_2$**	**M^{s}Fluind-Bi(I)**
Space group	*P*2$_1$/*c* (No. 14)	*P*2$_1$/*c* (No. 14)	*P*-1 (No. 2)
Z	2	2	2
a / Å	14.8179(3)	14.8042(12)	12.6987(3)
b / Å	16.1857(3)	16.0940(14)	14.3359(3)
c / Å	21.4312(4)	21.3660(18)	15.3368(4)
α /°	90	90	108.811(1)
β /°	99.094(1)	98.731(4)	102.403(1)
γ /°	90	90	106.274(1)
V /Å^3	5075.41	5031.65	2389.67

The structural similarity of the M^{s}Fluind-BiH_2 and M^{s}Fluind-SbH_2 compound becomes particularly clear when the molecules are superpositioned. This was carried out using the 'automatic molecule overlay' function in the 'Mercury' programme (Mercury 2023.2.0 (Build 382240)). The Bi or Sb atoms of the compared structures were selected.

The smallest RMSD for the overlay of M^{s}Fluind-BiH_2 with M^{s}Fluind-Bi(I) was 0.3430. In comparison, a much smaller value of 0.0315 was obtained for the overlay of M^{s}Fluind-BiH_2 with M^{s}Fluind-SbH_2.

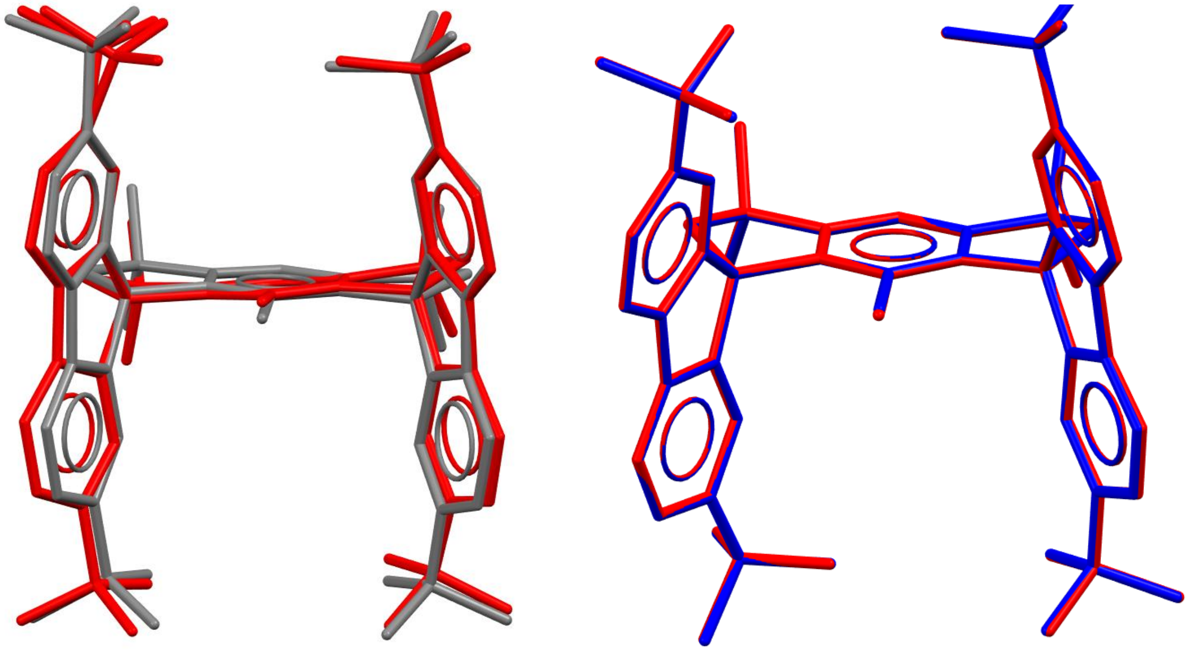

Figure S34. Superposition of M^{s}Fluind-BiH_2 (red) and M^{s}Fluind-Bi(I) (grey) (left) as well as M^{s}Fluind-BiH_2 (red) and M^{s}Fluind-SbH_2 (blue) (right)

All these considerations lead to indirect evidence for the presence of two H atoms. We therefore interpret the structural model as M^{s}Fluind-BiH_2, although a clear localisation of the hydride H atoms from the residual electron density distribution is not possible beyond doubt. Due to the fixation of the H positions during the refinement, an exact discussion of the Bi-H distances and angles is also not possible with this model.

7. Theoretical Study

7.1. General information.

All quantum chemical calculations in the present work were performed using the ORCA 6.0 program[3] suite employing the scalar relativistic zeroth order regular approximation (ZORA).[4,5] Geometry optimizations were carried out using the BP86 density functional[6] in conjunction with the ZORA-Def2-SVP basis set for hydrogen (H) except Bi-H and carbon (C).[7] For bismuth bounded hydrogen (H), the ZORA-Def2-TZVP was used. For bismuth (Bi) the SARC-ZORA-TZVP basis set was used which features a contraction optimized for the ZORA Hamiltonian.[8] The RI approximation with SARC/J fitting basis set was employed to accelerate the calculations.[9-12] Subsequent frequency calculations revealed that all optimized geometries are local minima having no imaginary frequencies.

7.2. Theoretical study for Bi–H stretching

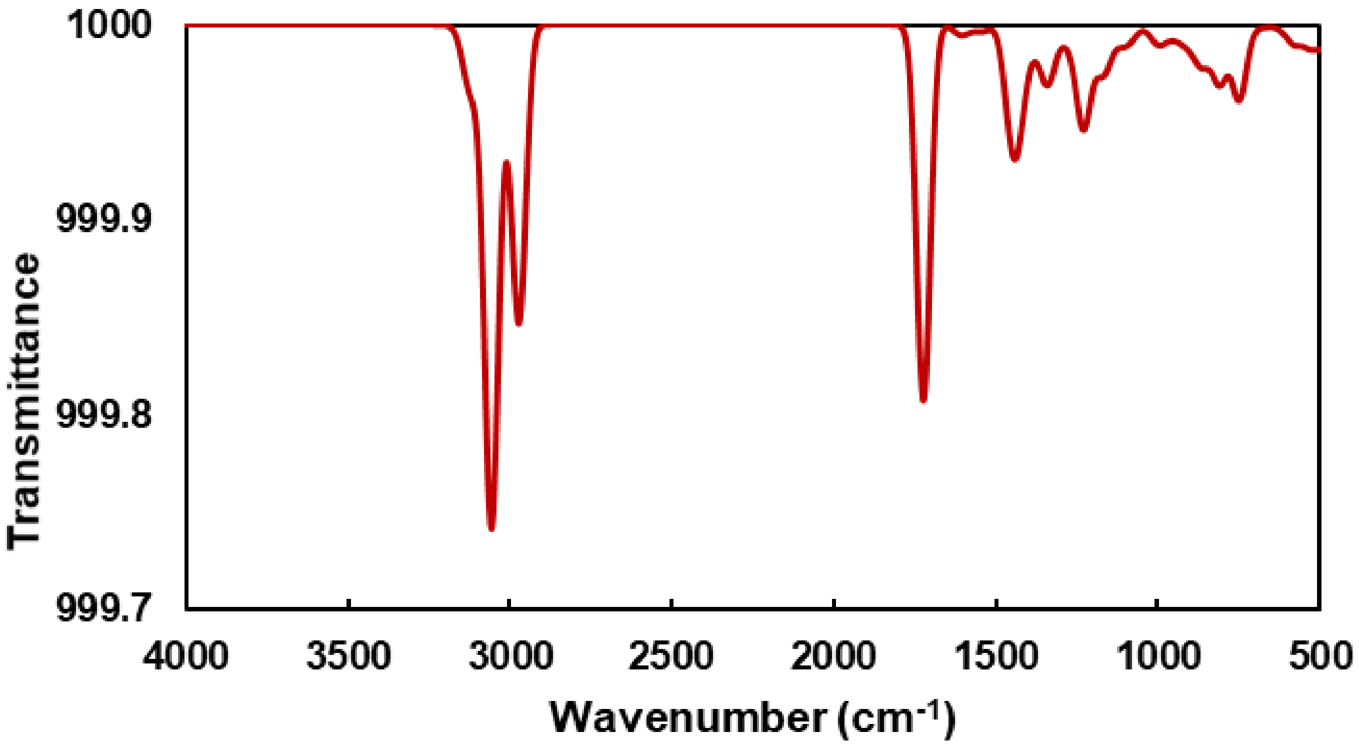

Figure S35. Predicted IR spectrum of **1**.

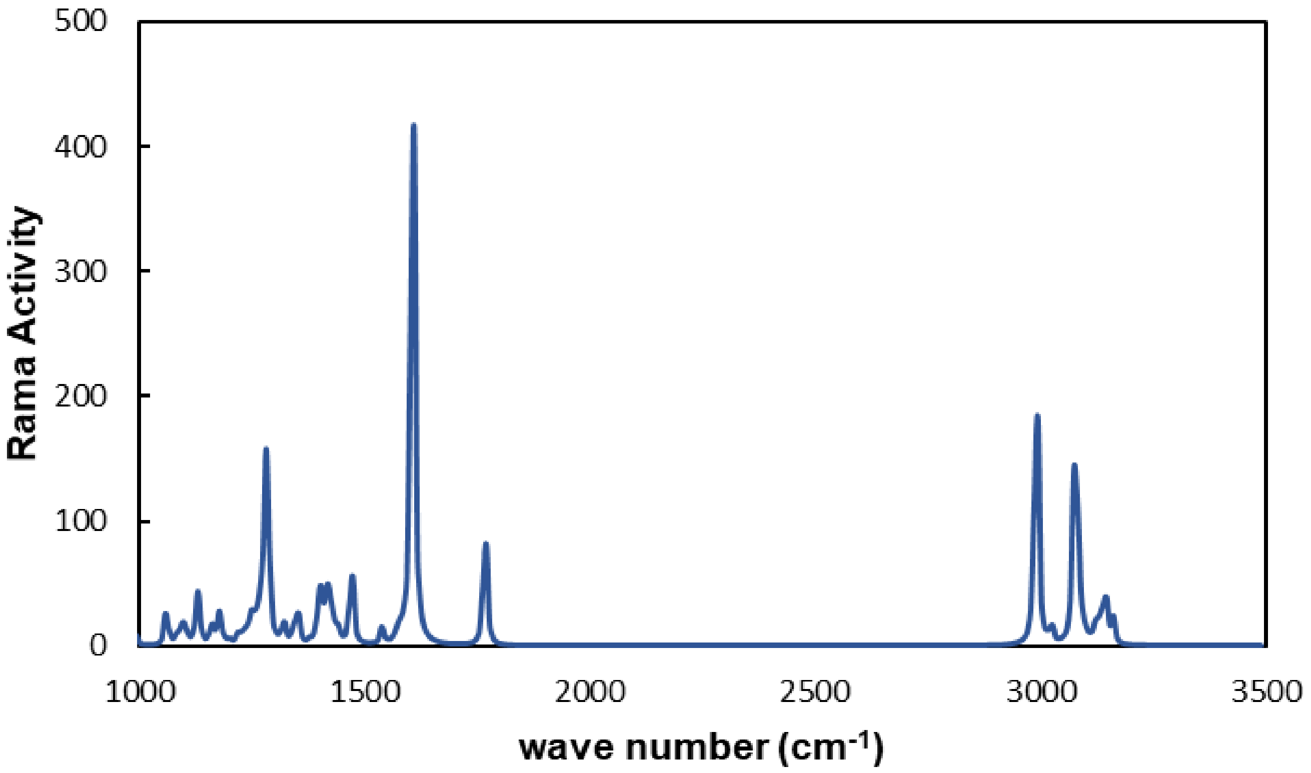

Figure S36. Predicted Raman spectrum of **1**.

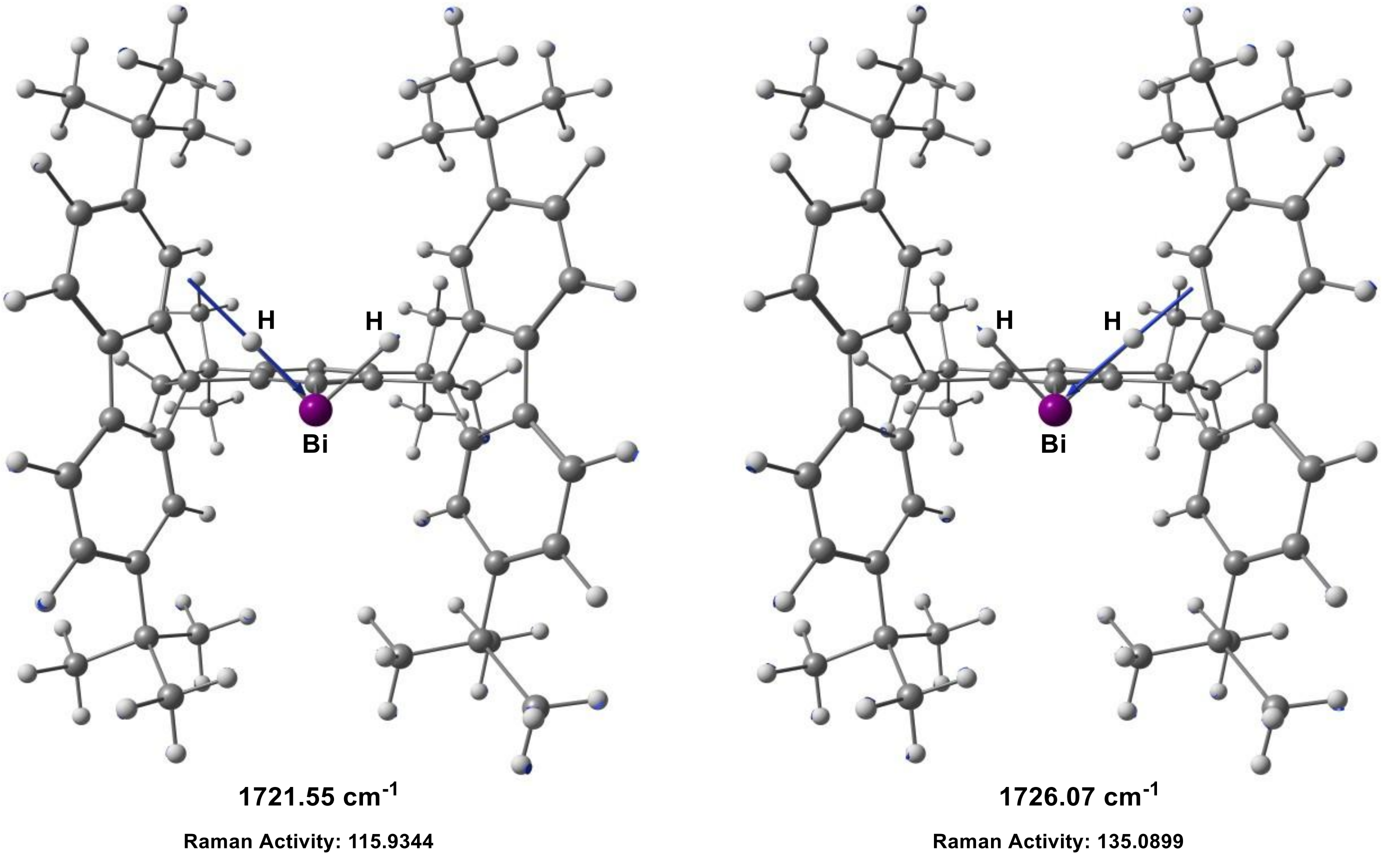

Figure S37. Selected vibrations corresponding to the Bi–H stretching.

7.3. Theoretical study for Bi–D stretching

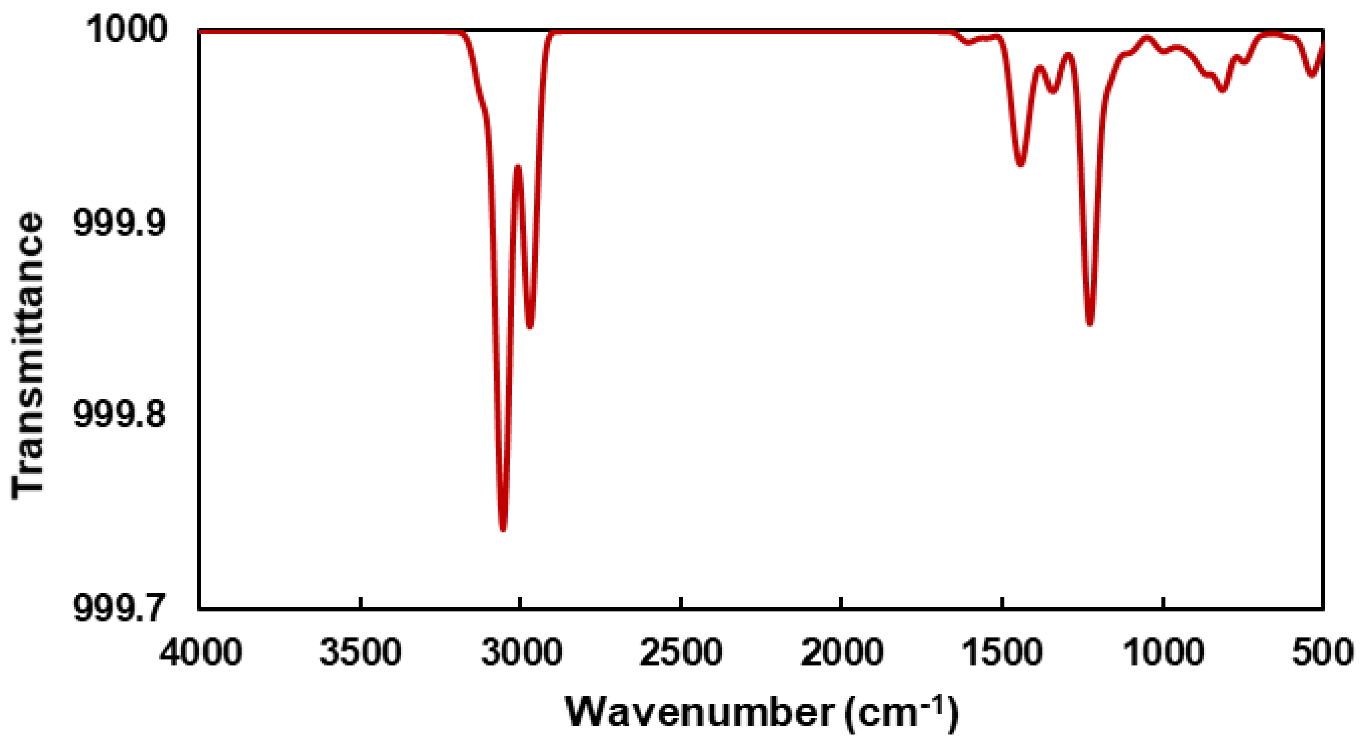

Figure S38. Predicted IR spectrum of **1**-d_2 based on DFT calculations.

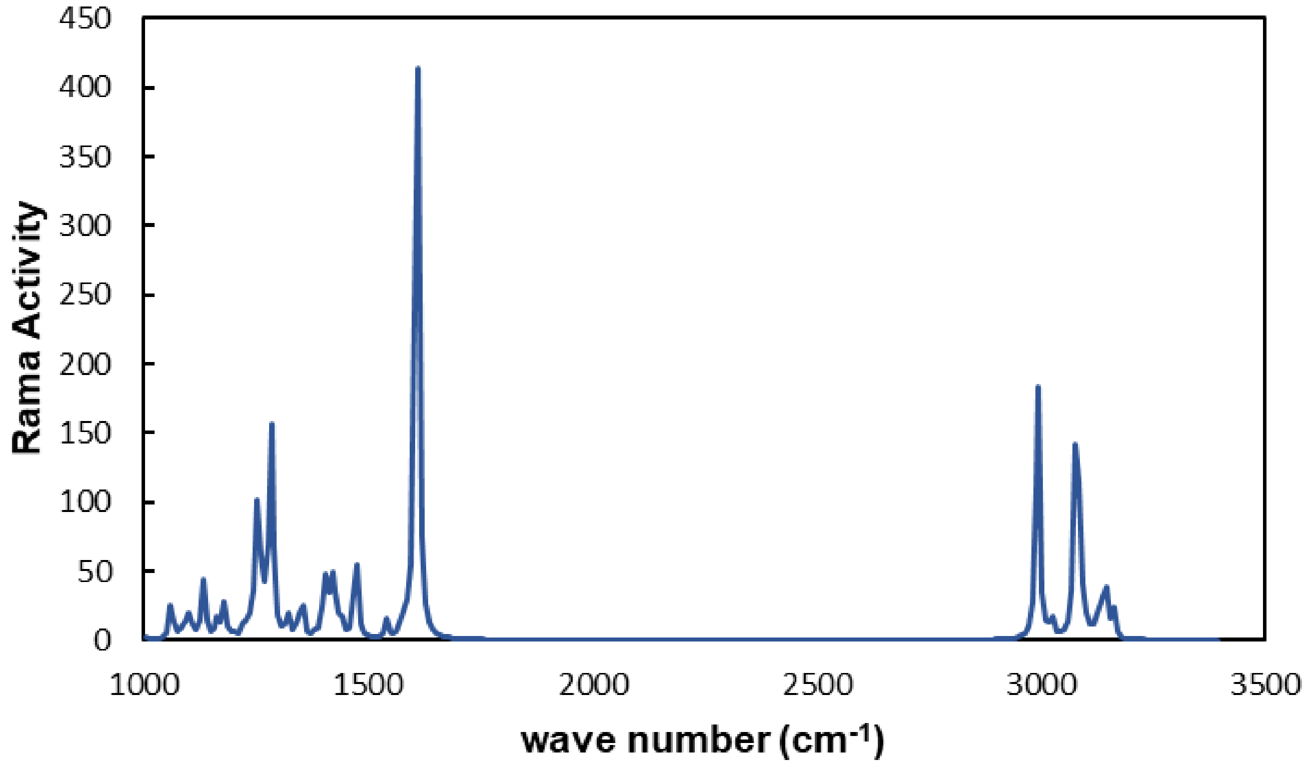

Figure S39. Predicted Raman of **1**-d_2 based on DFT calculations.

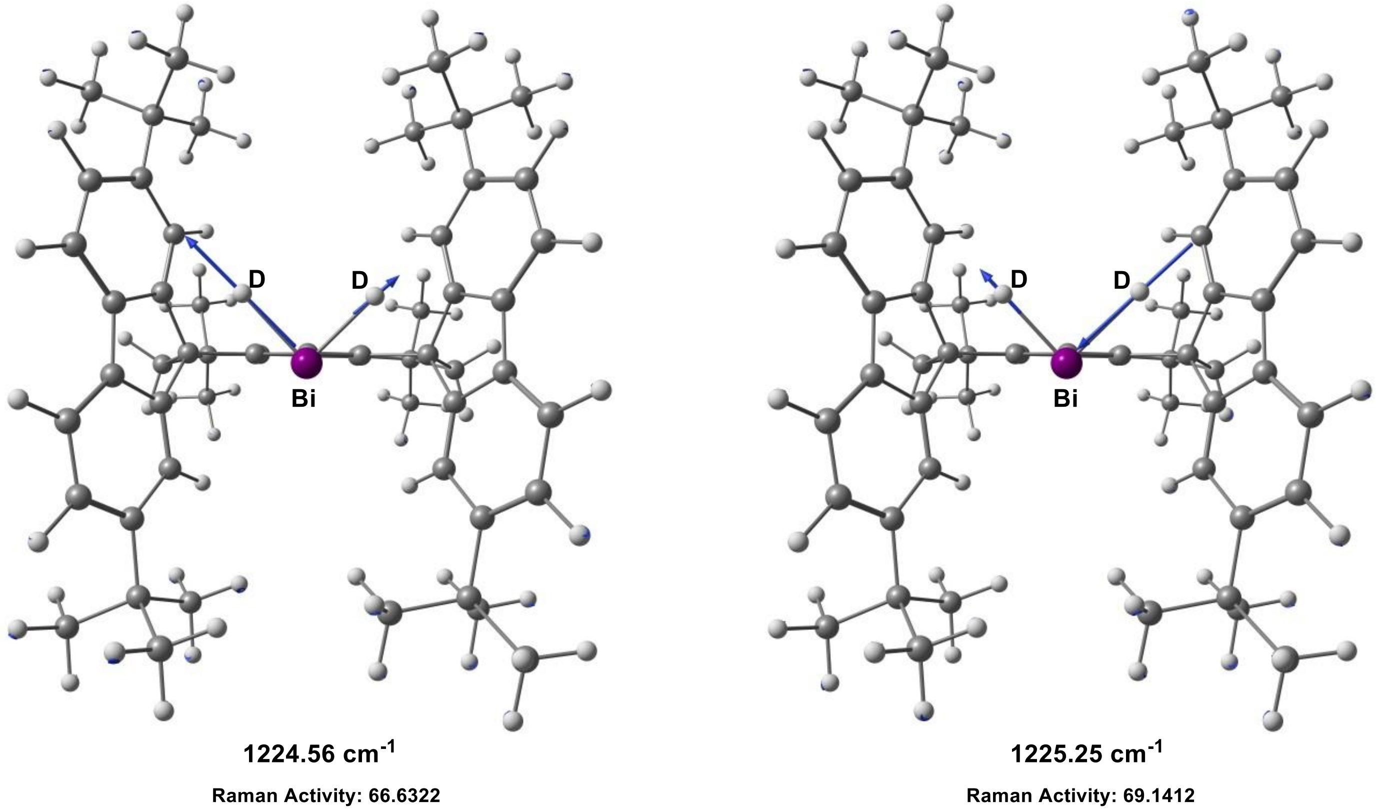

Figure S40. Selected vibrations corresponding to the Bi–D stretching.

7.4. Theoretical study of dynamics

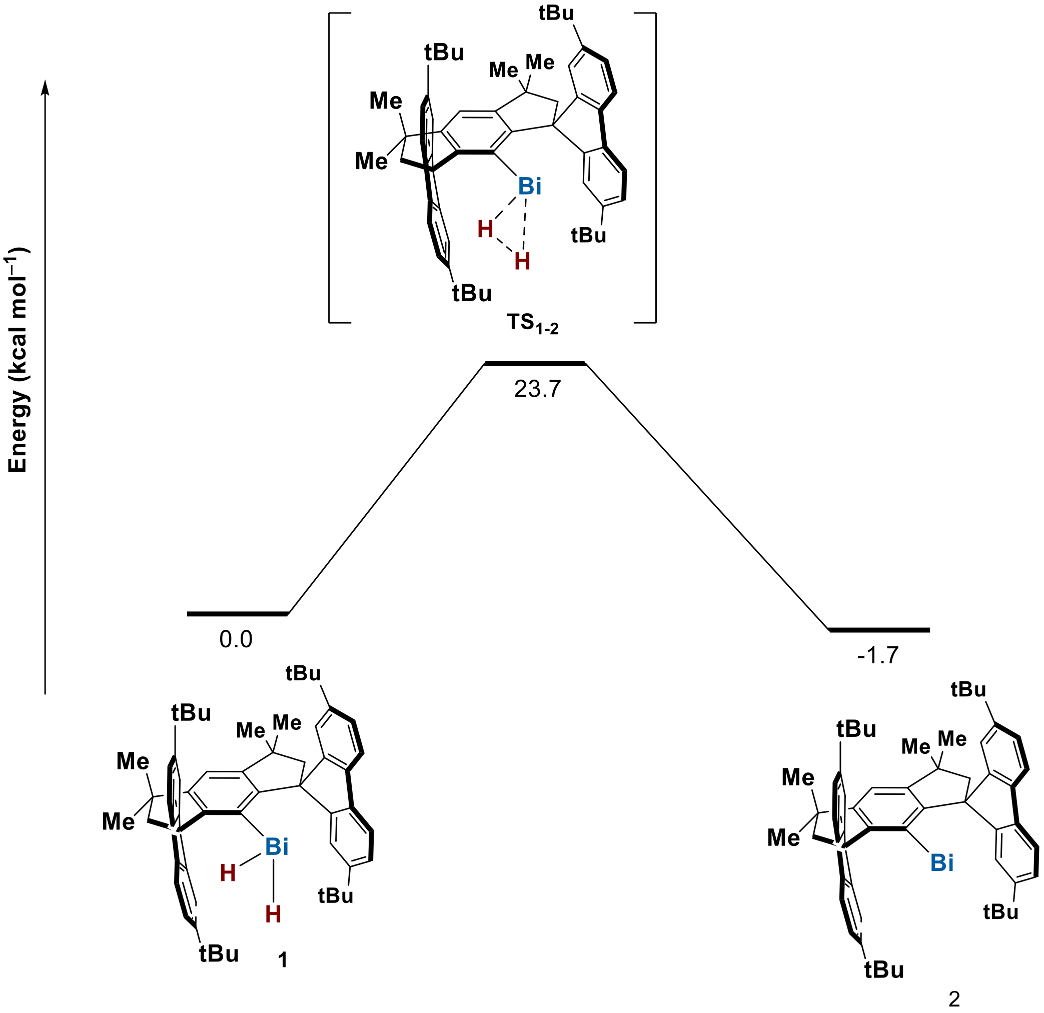

Figure S41. Energy profile for reductive elimination.

7.5. Coordinates

Table S8. Coordinates of the optimized **1.**

Bi	-0.89721033201789	-1.02890165176185	6.85288042591492
C	0.78881868947939	0.18991804962606	7.85498359395700
C	0.57602532455695	1.53888312241870	8.24398584941083
C	-0.73071748169011	2.35799915475982	8.24328686881131
C	-0.20640286570596	3.83397000299518	8.48500949318322
H	-0.17793064482340	4.34971365262586	7.50898532540039
H	-0.89692109106297	4.40541959062906	9.12988214476263
C	1.23932762459372	3.76652225312802	9.06237514284341
C	1.64451629250922	2.33115034613542	8.73785943127454
C	2.93526371184730	1.79808510422499	8.86335095010114
H	3.76079122926391	2.41898636142913	9.23790269822605
C	3.16223530880692	0.46978371498519	8.47508977888114
C	4.50599915739863	-0.25164778123727	8.51847528632652
C	4.19850434895508	-1.56912364836078	7.74325483141565
H	4.61949357980050	-1.49361671789951	6.72553299390592
H	4.65985493565198	-2.45362508800551	8.21617260402925
C	2.62811101738663	-1.73957857617457	7.61797149972249
C	2.10576462826713	-0.33034510420732	7.97046120914335
C	1.28186640756107	4.03924509024402	10.58460971481024
H	0.60986029152243	3.36626932554599	11.14339536067325
H	2.30481243282060	3.90326979340610	10.98281355296514
H	0.97219938128260	5.07975490304158	10.79875999336422
C	2.15660480694530	4.78861654960491	8.35233590108333
H	3.18875979363220	4.74814603198131	8.74789547226714
H	2.20149599440978	4.60273966896564	7.26329834563081
H	1.78264664298008	5.81870392245581	8.50713248314347
C	4.94780509532860	-0.51554346074716	9.97762412902288
H	5.08638605533553	0.43454804198777	10.52702637277428
H	4.20669343223453	-1.11851940918695	10.52991397210002
H	5.91083700981209	-1.06033923654701	9.99964076836659
C	5.61335186455408	0.55546868153970	7.80398629123163
H	5.32190499996610	0.81337480861313	6.76903995634292
H	5.83807305427052	1.49703200616505	8.33920512628009
H	6.55114589358427	-0.03031095800063	7.75707431875076
C	-1.76763003626834	1.95558961901404	9.31118577691842
C	-1.57772535108441	1.64421450230635	10.66484881410086
H	-0.56067799931638	1.60523868769880	11.07007705196991
C	-2.68493465538054	1.36356263601766	11.50417303811210
C	-3.98226944606602	1.40391962150157	10.93085306613508
H	-4.85722316825575	1.19301977650325	11.55913738263557
C	-4.18620060880845	1.69179346034075	9.57205128224978
H	-5.20244296406478	1.69253831399469	9.15626159622691
C	-3.07338005406945	1.96075135521477	8.75472645336285
C	-2.97063991401467	2.21273198908826	7.31278846511842

C	-3.94658464106944	2.29361162020484	6.30660677123376
H	-5.01287941882133	2.18770501573609	6.54622498436930
C	-3.54566457429184	2.51898033865156	4.97559899611151
H	-4.32173750244301	2.58404002521278	4.20504557854587
C	-2.18397400686641	2.66422166335601	4.61686498955129
C	-1.21138462020161	2.57738480065355	5.64958785092583
H	-0.14580691515882	2.69075998963002	5.40770501695943
C	-1.59743015496952	2.35611080279746	6.97640183773404
C	-2.52741003923972	1.02027418788119	13.00244515622010
C	-3.11327672358893	-0.39034288967667	13.27180682401228
H	-4.18579406419518	-0.45045121633400	13.01233561654379
H	-2.58058783885233	-1.16225789462030	12.68573141727650
H	-3.01412793325156	-0.65098221550417	14.34287613341003
C	-3.29898336165915	2.06420984279435	13.85142751115649
H	-2.90197425851884	3.08374213808808	13.68904142333887
H	-4.37683851163566	2.07890244063710	13.60875551299741
H	-3.20282811635413	1.83059282557509	14.92889906919837
C	-1.05262669448824	1.02462193300112	13.45435862178602
H	-0.44819146016042	0.27817804661033	12.90739244266122
H	-0.57707869103314	2.01375701013878	13.32032344268413
H	-0.99211052269355	0.77371576888641	14.52961242910505
C	-1.73266736770144	2.90936657415444	3.15968819496177
C	-0.86525156945836	1.71158918072089	2.69391481386304
H	0.03134011604573	1.59096516495284	3.32786887389276
H	-1.43854990568335	0.76700268867645	2.73367825932933
H	-0.52358454515431	1.86014150853079	1.65210523552478
C	-2.92502624302397	3.05553060380291	2.19097742719857
H	-3.54809477114778	2.14289353026852	2.15268405366375
H	-3.57854590551972	3.90500200412494	2.46329382391791
H	-2.55150133596620	3.24095929514757	1.16683909954970
C	-0.88864615390218	4.20811000531113	3.08940543245788
H	-1.47413648891535	5.08494153711444	3.42378070888246
H	0.01805724333206	4.14730283259193	3.71788214939722
H	-0.56023119556756	4.39678577600091	2.04969062907893
C	2.11544897296030	-2.88648513918866	8.50959868140841
C	2.04014071231654	-2.95856944242989	9.90729690682136
H	2.32355330659123	-2.08615498281517	10.50617585798070
C	1.59192680880847	-4.14124489307427	10.54725751465838
C	1.21818498378324	-5.23913643928140	9.72964810310057
H	0.86836924240453	-6.16792989693735	10.19870462815169
C	1.27375804942880	-5.17614507816414	8.32803136114361
H	0.96331136880316	-6.04019907957349	7.72576803975592
C	1.72016529155699	-3.99248255167864	7.71322899182634
C	1.83362229530244	-3.61878173070700	6.29817452942267
C	1.56435958828696	-4.33265801931938	5.11505300453562
H	1.20245085878159	-5.36867508764117	5.15170628273691
C	1.77931009095967	-3.70742975090382	3.87641462967204
H	1.57717669573306	-4.27884945885223	2.96121267974547

C	2.26058634863853	-2.37479368676516	3.77457143968441
C	2.50825344616676	-1.66754997439797	4.97644553122594
H	2.88480018576484	-0.63810198590458	4.94560451616006
C	2.29705627688709	-2.28176662931454	6.22063196776330
C	1.50520621729881	-4.27439067990098	12.08442447958346
C	0.04008693583676	-4.57358961069996	12.49443640212901
H	-0.63647840951536	-3.75550433169950	12.18436351497718
H	-0.33324764024022	-5.50999030798943	12.04192879211264
H	-0.03846897268787	-4.67955791165017	13.59326350764845
C	2.41399223253376	-5.44315076985872	12.54717684791785
H	2.11096048511957	-6.40554706666380	12.09645877130224
H	3.47015887601365	-5.26017793812373	12.27416476216229
H	2.36356670514720	-5.55869754572501	13.64666238503004
C	1.95945597122825	-2.99181284624376	12.81132248682324
H	3.01061239453236	-2.73485240831981	12.58455164530194
H	1.33060789993485	-2.12135349751302	12.54956760300310
H	1.88361324960057	-3.13626913240394	13.90498934847767
C	2.53258465273044	-1.76793934820514	2.37965706417432
C	3.61958098558545	-2.61495443233017	1.66620040005678
H	4.56326710354643	-2.62371512188859	2.24306596602735
H	3.29934131967668	-3.66340809444843	1.52672956582161
H	3.83756558231099	-2.19603893205750	0.66535005022264
C	1.23453711438767	-1.79293540850088	1.53337058985804
H	0.84653714707140	-2.81910649686795	1.40186110086957
H	0.43789798506061	-1.18736446891758	2.00385476794645
H	1.42514126738581	-1.37694910577997	0.52584750937816
C	3.03341763437789	-0.31101545767196	2.46349209686359
H	3.21062307579777	0.08161270585977	1.44518022391538
H	2.29605038159645	0.35323623552455	2.95030413365969
H	3.98606906655392	-0.23093656650755	3.01864653646332
H	-2.09456502381468	-0.51991733997912	8.11440255225227
H	-0.56034298021616	-2.48324373566620	7.88267708974112

Table S9. Coordinates of the optimized **1-d_2.**

Bi	-0.89788615777146	-1.02205639165157	6.84517889713434
C	0.78865193134296	0.19100107879823	7.85318866259378
C	0.57745804185642	1.53945691902005	8.24459218896369
C	-0.72888688655636	2.35913724992223	8.24611684430887
C	-0.20428742957678	3.83450796864785	8.48980083648770
H	-0.17960129227352	4.35329623746086	7.51531507793396
H	-0.89276790598471	4.40366188447450	9.13884652264677
C	1.24351604818795	3.76623949640695	9.06259714889040
C	1.64667184624063	2.33015729483346	8.73893152691230
C	2.93662521141394	1.79508392527169	8.86475267419880
H	3.76290153220560	2.41442447289274	9.24023573109535
C	3.16182959701146	0.46675228766502	8.47562826752385
C	4.50429498787384	-0.25722561468472	8.52029361434401

C	4.19624895799054	-1.57009989737274	7.73795763127760
H	4.61413022223082	-1.48684330558504	6.71953560702520
H	4.65964029673058	-2.45755847334125	8.20334666253871
C	2.62545628759773	-1.74035619394664	7.61436714446783
C	2.10472647601224	-0.33120379069211	7.96850126782502
C	1.29171489572210	4.04170258221529	10.58411350016283
H	0.62132105337861	3.37014124414619	11.14657120121236
H	2.31602098006896	3.90562882813131	10.97875574902670
H	0.98366926042134	5.08280900819672	10.79766742399516
C	2.15971668736403	4.78589147069152	8.34747590406379
H	3.19329599673261	4.74435026147371	8.73917992250534
H	2.20016907334456	4.59821044219546	7.25858220910835
H	1.78796919488368	5.81681318369048	8.50205965675680
C	4.94084474683139	-0.52878749045865	9.97963028658816
H	5.07814939548173	0.41841895820755	10.53431268232666
H	4.19771136620132	-1.13417835522811	10.52640519135022
H	5.90348169922768	-1.07427576004085	10.00207972735719
C	5.61595271817912	0.55038808079854	7.81322529890584
H	5.32804278044119	0.81537452517426	6.77908490281481
H	5.84265680569128	1.48800912534011	8.35451156306253
H	6.55189942176983	-0.03825207620611	7.76521807210401
C	-1.76448088082010	1.95403204280349	9.31428195668838
C	-1.57335947113156	1.64685401913122	10.66875689161954
H	-0.55619216426248	1.61402064270205	11.07422406865240
C	-2.67944629295915	1.36372073528326	11.50869564431426
C	-3.97683300611993	1.39640720283856	10.93493000847532
H	-4.85092085217603	1.18315322421331	11.56362142899612
C	-4.18185569466103	1.68024144073767	9.57544256835933
H	-5.19805043116385	1.67571602701794	9.15957613034130
C	-3.07011753907630	1.95265288478113	8.75777576338162
C	-2.96852455185156	2.20508280108272	7.31582123875214
C	-3.94488142586703	2.28220010708327	6.30969418384687
H	-5.01061247216286	2.17033879470340	6.54908865771521
C	-3.54509546299548	2.51204625979523	4.97912615333606
H	-4.32146745694542	2.57440682664316	4.20866329396397
C	-2.18413506226967	2.66521739963382	4.62066725054259
C	-1.21120388125006	2.58145597243510	5.65318127386650
H	-0.14619898042787	2.70071394321773	5.41159644613345
C	-1.59615188185681	2.35600847757003	6.97964933667295
C	-2.52084605193015	1.02668205552673	13.00827530878131
C	-3.10223636109474	-0.38465111368565	13.28334255212878
H	-4.17455783375808	-0.44922331253338	13.02411593986695
H	-2.56714589360125	-1.15736934832246	12.70053100084503
H	-3.00240706802654	-0.64061383172304	14.35547813807469
C	-3.29576158895438	2.07160961850611	13.85302883038362
H	-2.90218477370161	3.09176897686580	13.68631830725349
H	-4.37370101741006	2.08168444562303	13.61048618320472
H	-3.19862031842004	1.84284660100340	14.93145544424605

C	-1.04608261754642	1.03759988715957	13.46015045632267
H	-0.43914018065928	0.29139569293527	12.91564650844941
H	-0.57391335701448	2.02788640128275	13.32272109630130
H	-0.98461212460794	0.79057475745904	14.53624808605877
C	-1.73436288578545	2.91638952134956	3.16403609435100
C	-0.86425830674218	1.72226627848304	2.69365693022206
H	0.03220088094905	1.60022128056035	3.32755505063490
H	-1.43584251155907	0.77646671930951	2.72865133121107
H	-0.52209624810693	1.87618347801053	1.65278167307010
C	-2.92770592890713	3.06238386978113	2.19652420662530
H	-3.54798010540898	2.14797975732437	2.15540196679523
H	-3.58382928410040	3.90882883998937	2.47202258950078
H	-2.55542075268833	3.25253817758810	1.17279954588846
C	-0.89369772150778	4.21741651477430	3.09734668712941
H	-1.48108616318738	5.09178822830963	3.43479417189477
H	0.01361610583718	4.15678520188212	3.72496545378606
H	-0.56642711524382	4.41015507787762	2.05802437946575
C	2.11420617528510	-2.88823456082791	8.50565710251957
C	2.03248884968984	-2.95904910090826	9.90306195061586
H	2.30704527781827	-2.08404748864983	10.50224812968284
C	1.58840526874751	-4.14367723350174	10.54235063410338
C	1.22633513821993	-5.24522866929179	9.72448379692282
H	0.88138311380790	-6.17604137844612	10.19312502466104
C	1.28644907098394	-5.18301516732168	8.32301554635720
H	0.98341771332006	-6.04943821987022	7.72037951580601
C	1.72736658669414	-3.99699712443465	7.70880188328481
C	1.84080240196874	-3.62283831036813	6.29389371004202
C	1.57645328400017	-4.33824403956458	5.11064411788761
H	1.22077815946061	-5.37642339388789	5.14708911712280
C	1.78777409346562	-3.71149467128739	3.87214381340177
H	1.58932920605586	-4.28393267826157	2.95677078645398
C	2.26070679343770	-2.37587912655923	3.77068949335090
C	2.50411123333665	-1.66741447854668	4.97273847012370
H	2.87444658131398	-0.63572125467276	4.94201318347738
C	2.29620515631380	-2.28304244489179	6.21679770766499
C	1.49568891087446	-4.27563742281254	12.07929774566434
C	0.03472426609214	-4.60321150659366	12.48264876513305
H	-0.65672718506916	-3.79981275213807	12.16704262424128
H	-0.31717416727179	-5.54799690422679	12.03061564843090
H	-0.04725398150063	-4.70820079956409	13.58129299277511
C	2.42500076808468	-5.42563762000336	12.54833718108359
H	2.14430477330558	-6.39392976429251	12.09567549329749
H	3.47892863566165	-5.22136395527492	12.28192881083430
H	2.36996206671727	-5.54207957473672	13.64752129254774
C	1.92064501694169	-2.98301070265097	12.80601863176548
H	2.96841205459784	-2.70687560003307	12.58625892639704
H	1.27749511649592	-2.12536902829181	12.53674480670764
H	1.83960089775143	-3.12579578965492	13.89952084526194

C	2.52764800423576	-1.76660771016627	2.37583552496671
C	3.61352001212451	-2.61048700567319	1.65718228772930
H	4.55904751816943	-2.61922827429342	2.23102941673155
H	3.29449605460204	-3.65906522540053	1.51598859497676
H	3.82788883438454	-2.18891783979018	0.65666482046652
C	1.22653232035330	-1.79232577631159	1.53421524982215
H	0.83966777778380	-2.81895419690040	1.40292938120207
H	0.43079751928897	-1.18859941334072	2.00861148860743
H	1.41248054707114	-1.37458965980271	0.52652557360116
C	3.02648187472342	-0.30905033865693	2.46038979990266
H	3.20158328000187	0.08486714351428	1.44220704744159
H	2.28903046950696	0.35388194376288	2.94889634727983
H	3.97989720026467	-0.22831088732146	3.01416403170381
D	-2.09283221296033	-0.53803430891584	8.12000941045019
D	-0.54830158524923	-2.48716944259507	7.85497345183661

Table S10. Coordinates of the optimized $\mathbf{TS_{1\text{-}2}}$.

Bi	-0.67764714	-0.06961238	-1.50481658
C	-0.00434178	0.00442750	0.70280767
C	0.25018729	-1.19191330	1.42412207
C	0.29572534	-2.64626244	0.91292397
C	0.31214543	-3.47963494	2.26090268
H	-0.71335592	-3.84333203	2.44930894
H	0.95799538	-4.37107942	2.17467306
C	0.73664513	-2.53962565	3.42890090
C	0.53515452	-1.15735009	2.81367984
C	0.58228957	0.06049850	3.50690344
H	0.79453041	0.08048753	4.58479838
C	0.32465326	1.24948934	2.80935629
C	0.30632150	2.64952751	3.41566015
C	-0.27807052	3.50594906	2.25106111
H	-1.33671768	3.73193566	2.46759609
H	0.24004890	4.47439559	2.13924810
C	-0.21584559	2.66421607	0.90997946
C	0.02867272	1.22880193	1.42265003
C	2.20515744	-2.76069323	3.86247494
H	2.90455243	-2.66535451	3.01483860
H	2.50529164	-2.02631936	4.63324650
H	2.33207389	-3.77216639	4.29285745
C	-0.17765003	-2.75189376	4.65741091
H	0.10810688	-2.08642448	5.49343897
H	-1.23729440	-2.55405643	4.41127557
H	-0.10136628	-3.79391331	5.02246282
C	1.73072805	3.09821246	3.82025490
H	2.15146239	2.42903734	4.59414689
H	2.42313680	3.09591595	2.96094378
H	1.71176016	4.12349810	4.23636473
C	-0.60826985	2.72386157	4.65910655

H	-1.62889020	2.36394488	4.43246807
H	-0.20973039	2.11531466	5.49237569
H	-0.68802429	3.76703781	5.01996512
C	1.50626383	-3.00122396	0.02628514
C	2.85856885	-2.66988699	0.19032507
H	3.15531282	-2.01521172	1.01704016
C	3.83700732	-3.15871114	-0.71125824
C	3.40380794	-3.98521773	-1.78019579
H	4.14121885	-4.37922614	-2.49135445
C	2.05057004	-4.30679189	-1.97029864
H	1.74762499	-4.93082618	-2.82143659
C	1.09459005	-3.80582224	-1.06815686
C	-0.36928663	-3.90649048	-1.05526493
C	-1.26881654	-4.55482578	-1.91661201
H	-0.90691602	-5.14135622	-2.77137975
C	-2.65175831	-4.45150109	-1.67185499
H	-3.33794348	-4.96830555	-2.35180923
C	-3.16714200	-3.70911545	-0.58225423
C	-2.24036264	-3.05880722	0.27691210
H	-2.60526784	-2.47851386	1.13535316
C	-0.86360580	-3.15597795	0.04555217
C	5.33921595	-2.82582967	-0.56969604
C	5.82685564	-2.09339974	-1.84695494
H	5.69118787	-2.70839704	-2.75503231
H	5.27849254	-1.14476453	-1.99690140
H	6.90388877	-1.85190054	-1.76552591
C	6.14194746	-4.14189051	-0.39792773
H	5.82421541	-4.68844300	0.50968367
H	6.01499387	-4.81909462	-1.26173028
H	7.22257122	-3.92353473	-0.30127880
C	5.62858599	-1.92106128	0.64557764
H	5.10836453	-0.94855213	0.57285768
H	5.33395635	-2.39557107	1.59979646
H	6.71275561	-1.71132519	0.70366359
C	-4.68231124	-3.58704464	-0.30566936
C	-5.09926632	-2.09959871	-0.43863209
H	-4.55454968	-1.46121561	0.27975189
H	-4.89242161	-1.71867973	-1.45579084
H	-6.18153819	-1.97959549	-0.24252411
C	-5.52921864	-4.42204416	-1.28903509
H	-5.39781134	-4.09534957	-2.33717160
H	-5.28675203	-5.49952096	-1.23335955
H	-6.60158062	-4.31144347	-1.04292260
C	-4.98805286	-4.07570144	1.13369958
H	-4.69459110	-5.13361264	1.26839762
H	-4.45657726	-3.47768398	1.89573666
H	-6.07138010	-3.99537959	1.34444214
C	0.84897865	3.22197056	-0.05349117

C	2.24591856	3.20001102	0.05833359
H	2.70892716	2.68066289	0.90449339
C	3.05931563	3.83153536	-0.91569361
C	2.41527916	4.47591267	-2.00359487
H	3.02071467	4.97398174	-2.77198123
C	1.01759059	4.49278341	-2.13689189
H	0.55316465	4.98883469	-2.99940530
C	0.22912796	3.85879601	-1.15980166
C	-1.22307628	3.66899362	-1.05989113
C	-2.28267395	4.08629771	-1.88754365
H	-2.09101968	4.67042916	-2.79733044
C	-3.59978093	3.75899493	-1.52776388
H	-4.41791581	4.10065070	-2.17498860
C	-3.90201414	3.01866080	-0.35367764
C	-2.82084848	2.59267501	0.45604529
H	-3.00757299	2.02266573	1.37404601
C	-1.49990277	2.91357748	0.10677383
C	4.60214688	3.84709047	-0.82874146
C	5.19655984	3.15956382	-2.08499978
H	4.86966943	2.10538793	-2.15574883
H	4.89492807	3.66967153	-3.01762870
H	6.30241358	3.16880824	-2.04229652
C	5.09482286	5.31671130	-0.76943539
H	4.79043057	5.89088809	-1.66321847
H	4.69230296	5.83740949	0.11958314
H	6.19942214	5.35094923	-0.71069310
C	5.12716019	3.11043453	0.42083000
H	4.76249767	3.56910362	1.35839471
H	4.83702155	2.04392072	0.42657732
H	6.23197517	3.15158766	0.44126062
C	-5.37748987	2.74476824	0.01509870
C	-6.10206699	4.10017135	0.22931058
H	-5.63138399	4.68167102	1.04399453
H	-6.08742740	4.72393951	-0.68280770
H	-7.16127976	3.93077419	0.50149063
C	-6.07289868	1.97507458	-1.13630547
H	-6.04571612	2.53904165	-2.08607821
H	-5.59191541	0.99478511	-1.31064191
H	-7.13546549	1.79106903	-0.88816994
C	-5.51240414	1.91522358	1.30898561
H	-6.58156836	1.74138688	1.53076600
H	-5.02821167	0.92557812	1.21906098
H	-5.07415609	2.43330907	2.18172847
H	0.30119467	-1.68287291	-1.78649295
H	1.05360406	-0.36711117	-2.11626055

8. References

1) Pang, Y.; Nöthling, N.; Leutzsch, M.; Kang, L.; Bill, E.; van Gastel, M.; Reijerse, E.; Goddard, R.; Wagner, L.; SantaLucia, D.; DeBeer, S.; Neese, F.; Cornella, J. *Science* **2023**, *380*, 1043–1048.

2) Kratzert, D.; Krossing, I. *J. Appl. Cryst.* **2018**, *51*, 928–934.

3) Kratzert, D.; Holstein, J.J.; Krossing, I. *J. Appl. Cryst.* **2015**, *48*, 933–938.

4) Neese, F. WIREs *Comput. Mol. Sci.*, **2012**, *2*, 73–78.

5) Neese, F. *Wiley Interdiscip. Rev. Comput. Mol. Sci.* **2012**, *2*, 73–78.

6) Neese, F. *Wiley Interdiscip. Rev. Comput. Mol. Sci.* **2022**, *12*.

7) Becke, A. D. *Phys. Rev. A Gen. Phys.* **1988**, *38*, 3098–3100.

8) Weigend, F.; Ahlrichs, R. *Phys. Chem. Chem. Phys.* **2005**, *7*, 3297–3305.

9) Pantazis, D. A.; Neese, F. *Theor. Chem. Acc.* **2012**, *131*, 1292.

10) Pantazis, D. A.; Chen, X.-Y.; Landis, C. R.; Neese, F. *J. Chem. Theory Comput.* **2008**, *4*, 908–919.

11) Pantazis, D. A.; Neese, F. *J. Chem. Theory Comput.* **2009**, *5*, 2229–2238.

12) Pantazis, D. A.; Neese, F. *J. Chem. Theory Comput.* **2011**, *7*, 677–684.
